# New halimane and clerodane diterpenoids from *Croton cnidophyllus*

**DOI:** 10.1007/s13659-023-00386-z

**Published:** 2023-07-06

**Authors:** Xun Wei, Jia-Luo Huang, Hua-Hua Gao, Fang-Yu Yuan, Gui-Hua Tang, Sheng Yin

**Affiliations:** School of Pharmaceutical Sciences, Sun Yat-Sen University, Guangzhou, 510006 People’s Republic of China

**Keywords:** Euphorbiaceae, *Croton cnidophyllus*, Furanoditerpenoids, Anti-inflammatory activity

## Abstract

Three new halimane furanoditerpenoids (**1**–**3**) and three new clerodane furanoditerpenoids (**4**–**6**), along with seven known terpenoids including four pimarane diterpenoids (**7**–**10**) and three norisoprenoids (**11**–**13**) were isolated from the 95% EtOH extracts of the plants of *Croton cnidophyllus*. The 2D structures including absolute configuration of new furanoditerpenoids (**1**–**6**) were elucidated by analysis of their HRMS and NMR data as well as comparison of experimental and calculated ECD curves. Bioassay revealed that two compounds (**8** and **9**) possessed certain inhibitory effects against NO production stimulated by LPS, with IC_50_ values of 19.00 ± 1.76 and 21.61 ± 1.11 μM, respectively.

**Graphical Abstract:**

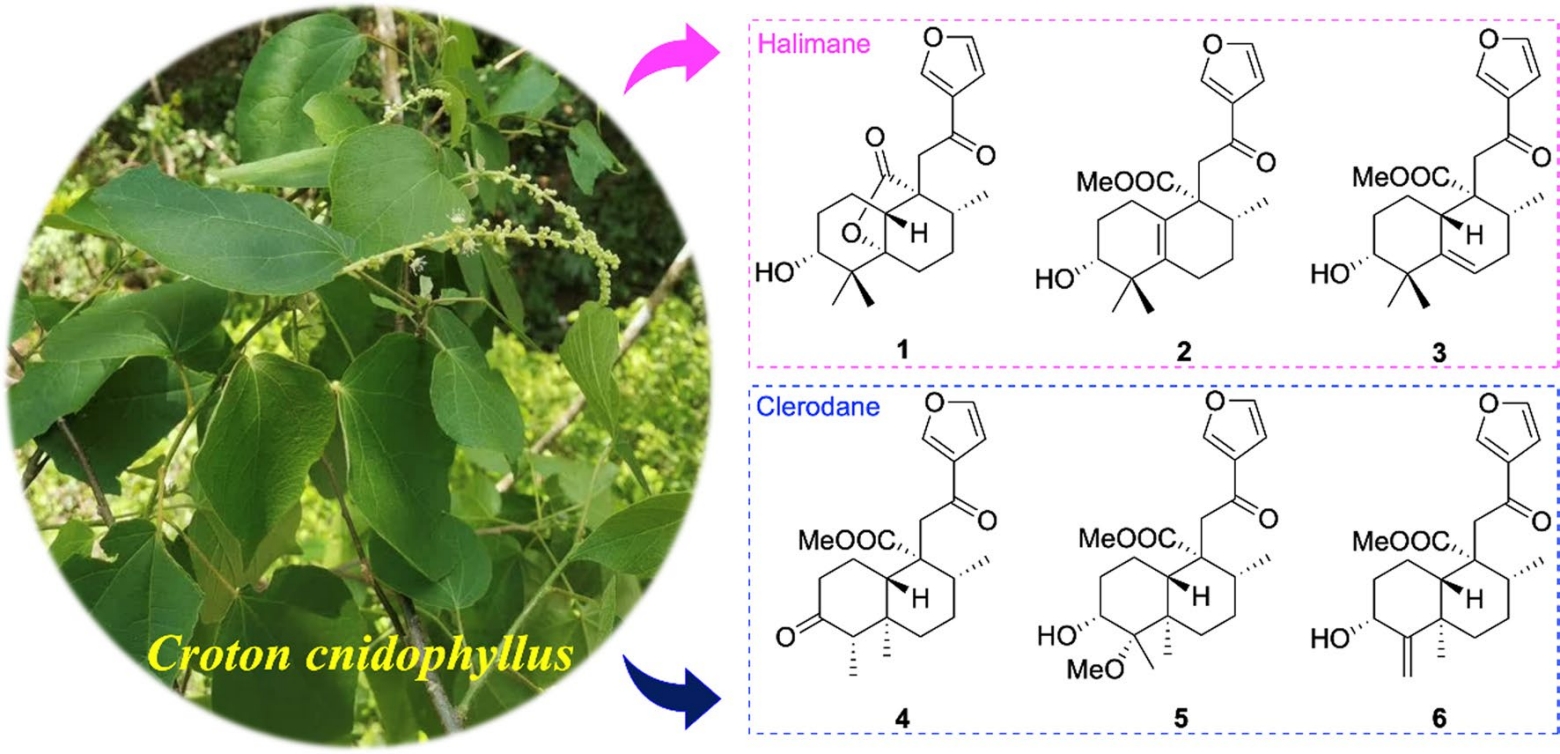

**Supplementary Information:**

The online version contains supplementary material available at 10.1007/s13659-023-00386-z.

## Introduction

Many plants of the genus *Croton* (Euphorbiaceae) such as *C. tiglium*, *C. crassifolius*, and *C. kongensis* are well-known traditional Chinese medicines, which have been used to treat stomachache, sore throat, rheumatism, and headache [1, 2]. Previous chemical investigation on some species of *Croton* revealed various types of diterpenoids including tiglianes [3], clerodanes [4], halimanes [1], labdanes [5], kauranes [6], casbanes [7], abietanes [6], isopimaranes [8], cembranes [9], and phytanes [10]. Some of these diterpenoids from the genus *Croton* exhibited diverse biological functions, such as cytotoxity [11], antiviral [12, 13], anti-plasmodial [14, 15], anti-microbial [15, 16], anti-inflammatory [1], and hypoglycemic activities [17]. The interesting structures of diterpenoids as well as their important bioactivities have made natural product chemists increasingly interested in studying plants of this genus.

**Fig. 1:**
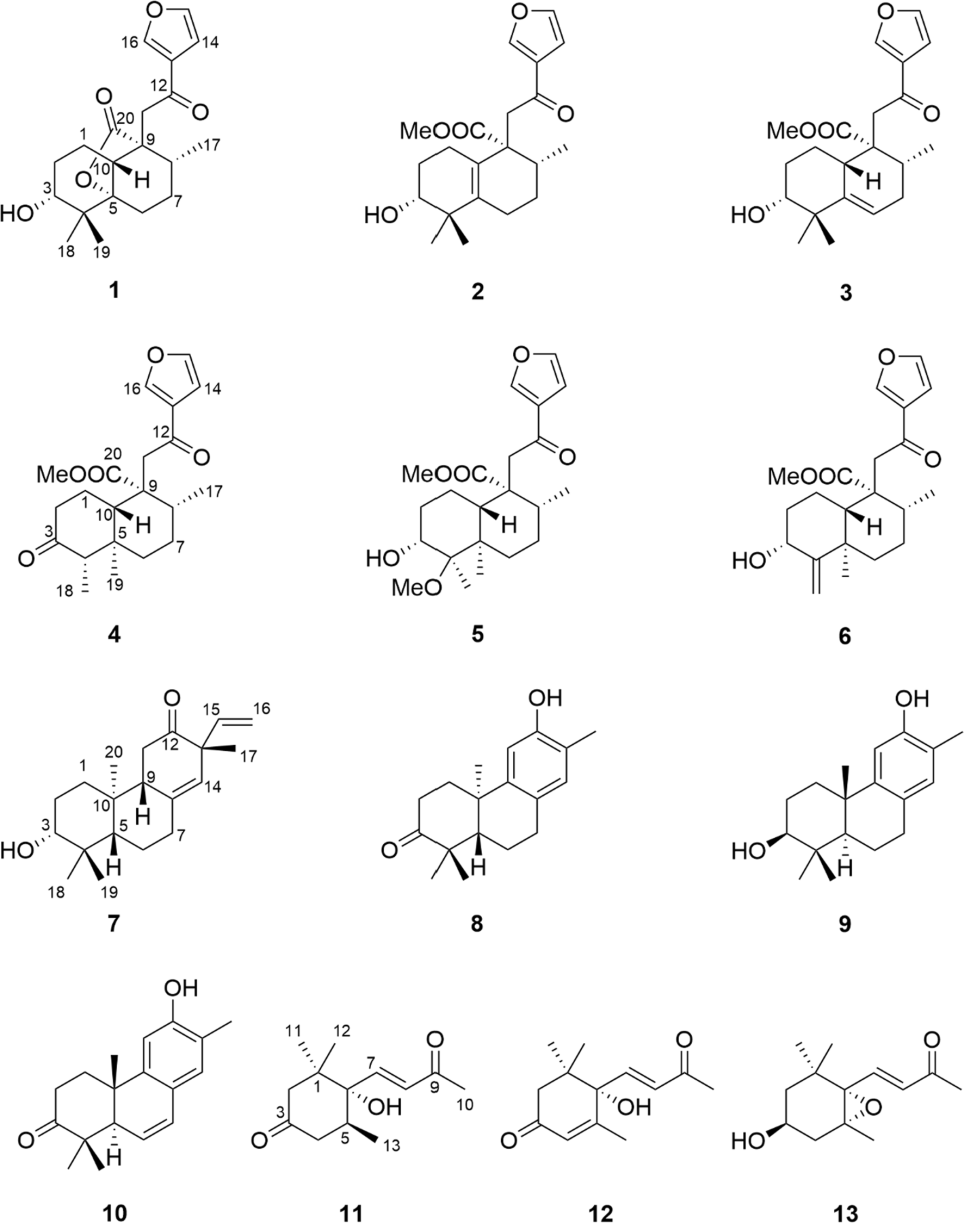
Chemical structures of compounds **1**–**13**

*Croton cnidophyllus* Radcliffe-Smith and Govaerts (synonym: *Croton urticifolius*) are shrubs, ranging from 1 to 2 m tall, mainly distributed in Guangxi, South Guizhou, and South Yunnan of China [18]. To our knowledge, there are currently no reports about the chemical constituents and bioactivities of *C. cnidophyllus*. In our continuous research aiming at the discovery of novel structures with biologically active diterpenoids from Euphorbiaceae [1, 19–21], the EtOAc fraction of the alcohol extracts of *C. cnidophyllus* was subjected to repeated column chromatography. A total of 13 compounds (**1**–**13**) were isolated and identified from *C. cnidophyllus* for the first time (Fig. 1). Of them, compounds **1**–**6** are new natural products*.* In addition, their inhibitory effects against the production of nitric oxide (NO) were evaluated in the LPS-induced RAW 264.7 cell model. Hence, the purification, structural elucidation of all terpenoids together with their bioactive assay are described.

## Results and discussion

Crodophylloid A (**1**) (Fig. 1) was acquired as colorless oil, and its molecular formula of C_20_H_26_O_5_ was determined by an HR-ESI-MS ion at *m/z* 369.1676 [M + Na]^+^ (calcd for 369.1672), corresponding to eight DOUs (degrees of unsaturation). The ^1^H NMR data of **1** (Table 1) exhibited resonances for one *β*-substituted furan [*δ*_H_ 6.76 (1H, dd, *J* = 1.9 and 0.8 Hz), 7.46 (1H, t, *J* = 1.6 Hz), and 8.11 (1H, br s)], three methyls [*δ*_H_ 0.83 (3H, d, *J* = 6.7 Hz), 0.96 (3H, s), and 1.24 (3H, s)], one isolated methylene [*δ*_H_ 2.89 (1H, d, *J* = 19.0 Hz) and 3.41 (1H, d, *J* = 19.0 Hz)], one oxygenated methine [*δ*_H_ 3.47 (1H, br s)], and other aliphatic multiplets. According to the DEPT and HSQC spectra, 20 carbon signals observed in ^13^C NMR spectrum were assigned as one conjugated ketocarbonyl (*δ*_C_ 192.6), one ester carbonyl (*δ*_C_ 177.8), a substituted furan moiety [*δ*_C_ 108.5 (CH), 127.6 (C), 144.6 (CH), and 147.6 (CH)], three quaternary carbons including an oxygenated one, three methines including an oxygenated one, five methylenes, and three methyls. The collective information suggested that compound **1** was a furanoditerpenoid.

**Table 1:** ^1^H and ^13^C NMR Data for Compounds **1**–**3** in CDCl_3_ (*δ* in ppm and *J* in Hz)

Position	**1**^*a*^		**2**^*b*^		**3**^*c*^	
	*δ*_H_, multi. (*J*)	*δ*_C_, type	*δ*_H_, multi. (*J*)	*δ*_C_, type	*δ*_H_, multi. (*J*)	*δ*_C_, type
1	1.45, m	18.6, CH_2_	*α* 1.62, m; *β* 2.06, m	27.1, CH_2_	1.60, m	22.5, CH_2_
2	1.74, m	26.2, CH_2_	1.64, m	27.3, CH_2_	*α* 1.67, m;	28.4, CH_2_
					*β* 1.87, m	
3	3.47, br s	76.2, CH	3.42, dd (10.6, 3.7)	75.9, CH	3.46, br s	76.6, CH
4		38.4, C		40.2, C		41.9, C
5		89.5, C		139.3, C		140.6, C
6	*α* 2.06, ddd (13.5, 5.6, 1.5);	29.9, CH_2_	a 2.05, m; b 2.11, m	25.2, CH_2_	5.57, br s	120.6, CH
	*β* 1.61, td (13.5, 5.8)					
7	*α* 1.28, m;	28.9, CH_2_	*α* 1.66, m	28.1, CH_2_	*α* 1.96, m;	31.9, CH_2_
	*β* 1.82, m		*β* 1.53, m		*β* 2.11, m	
8	2.35, m	33.8, CH	2.16, m	34.6, CH	2.41, q (6.8)	30.9, CH
9		55.4, C		54.6, C		52.6, C
10	2.61, dd (10.5, 7.8)	47.0, CH		124.9, C	2.84, br s	38.7, CH
11	a 3.41, d (19.0); b 2.89, d (19.0)	37.2, CH_2_	a 3.28, d (17.2); b 3.24, d (17.2)	41.3, CH_2_	a 3.26, d (16.9); b 3.16, d (16.9)	43.4, CH_2_
12		192.6, C		192.4, C		193.4, C
13		127.6, C		129.3, C		128.9, C
14	6.76, dd (1.9, 0.8)	108.5, CH	6.74, dd (1.9, 0.8)	108.7, CH	6.76, dd (1.7, 0.8)	108.8, C
15	7.46, t (1.6)	144.6, CH	7.42, t (1.7)	144.3, CH	7.43, m	144.5, CH
16	8.11, br s	147.6, CH	8.05, br s	147.0, CH	8.04, br s	147.3, CH
17	0.83, d (6.7)	16.9, CH_3_	0.86, d (7.0)	17.6, CH_3_	1.02, d (6.8)	17.9, CH_3_
18	1.24, s	21.0, CH_3_	1.00, s	19.7, CH_3_	1.17, s	25.5, CH_3_
19	0.96, s	24.4, CH_3_	1.08, s	24.5, CH_3_	1.08, s	27.1, CH_3_
20		177.8, C		175.4, C		174.6, C
20-OMe			3.67, s	51.9, CH_3_	3.66, s	51.5, CH_3_

^a^^1^H NMR measured at 500 MHz and ^13^C NMR at 125 MHz

^b1^H NMR measured at 500 MHz and ^13^C NMR at 100 MHz

^c1^H NMR measured at 400 MHz and ^13^C NMR at 100 MHz

**Fig. 2:**
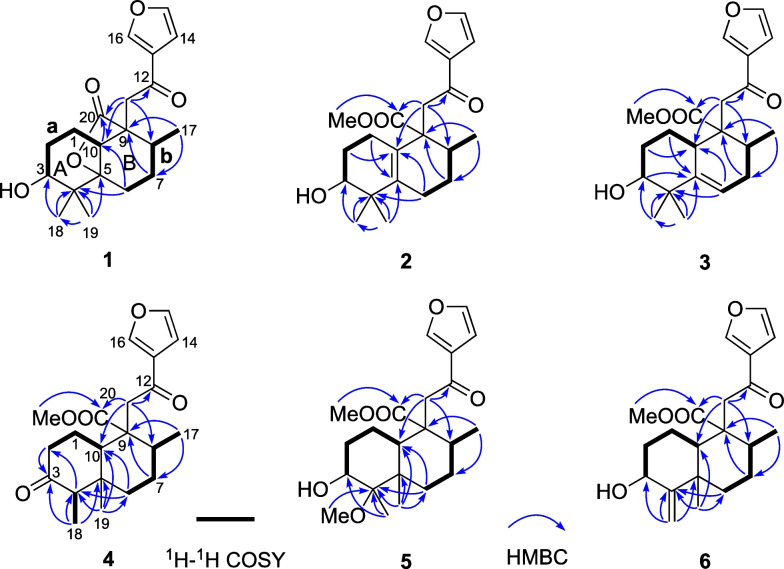
Key ^1^H–^1^H COSY and HMBC correlations of **1**–**6**

The 2D structure of **1** was determined on the basis of its 2D NMR spectra involving ^1^H–^1^H COSY and HMBC. The presence of fragments **a** and **b** as marked in Fig. 2 could be deduced by the ^1^H–^1^H COSY cross-peaks, which were linked to quaternary carbons C-4, C-5, and C-9 to constructed rings A and B, a 6/6 bicyclic skeleton with two methyls at C-4, by the HMBC correlations of H_3_-19/18 to C-5, C-4, and C-3, H_2_-6 to C-10, C-5, and C-4, H-8 to C-10 and C-9, and H_3_-17 to C-9. The 2-(furan-3-yl)-2-oxoethyl moiety could be confirmed by analyzing its 1D NMR data with the reported furanoditerpenoids crohalifuranes C–E [1], and its location at C-9 of ring B was proved by the HMBC correlations of H_2_-11 to C-10, C-9, and C-8. Besides, the ester carbonyl group (C-20) was also linked to C-9 as supported by the HMBC correlations from H_2_-11 and H-10 to C-20. The remaining one DOU (seven of the eight DOUs were represented by the ketocarbonyl, the ester carbonyl, the *β*-substituted furan moiety, and rings A and B) and the obvious downfield-shifted carbon chemical shift of C-5 (*δ*_C_ 89.5) manifested that there was a five-membered ring lactone formed between C-5 and C-20. Thus, the planar structure of **1** featured with a 5,20-*γ*-lactone moiety was elucidated.

**Fig. 3:**
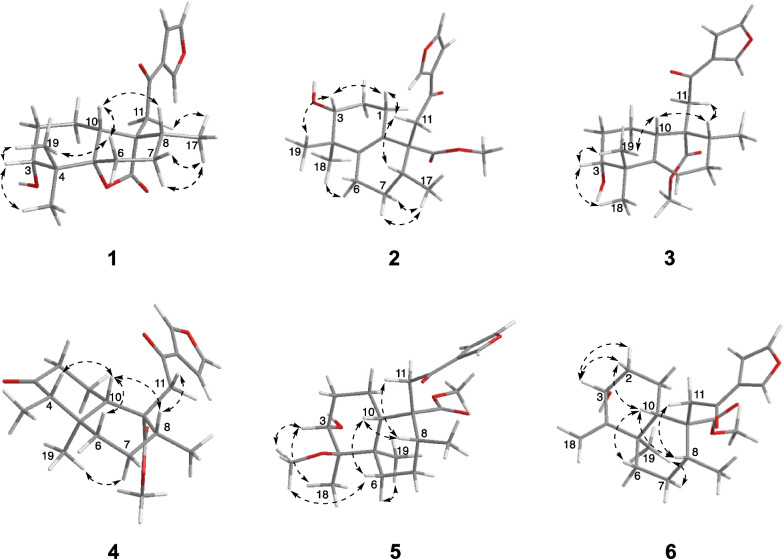
Key NOESY correlations of **1**–**6**

The relative stereochemistry of compound **1** was assigned through its NOESY experiment. The NOE cross-peaks of H-10/H-8 and H-6 and H_3_-19/H-6 suggested that H-10, H-8, Me-19, and H-6 occupied the axial position of the chair conformer of the A- and B-rings, and these groups were arbitrarily assigned to be *β*-oriented (Fig. 3), while the lactone bridge was occupied the *α*-orientation. The NOE cross-peaks of Me-18 (19)/H-3 assigned H-3 as equatorial *β*-orientation. Furthermore, the experimental ECD data of **1** displayed a negative Cotton effect at approximately 217 nm, which was consistent with other halimane derivatives isolated from *Croton* [1]. Therefore, the absolute configuration 3*R*,5*S*,8*R*,9*R*,10*R* was assigned to compound **1**, and it was the first example of halimane diterpenoid with a 5,20-*γ*-lactone moiety.

Crodophylloid B (**2**) (Fig. 1) had an HR-ESI-MS positive ion peak at *m*/*z* 383.1826 [M + Na]^+^ (calcd 383.1829), suggesting a molecular formula C_21_H_28_O_5_ for **2**. The ^1^H and ^13^C NMR data (Table 1) showed signals for one isolated methylene, a *β*-substituted furan, and a conjugated ketocarbonyl, which were undoubtedly determined to be a 2-(furan-3-yl)-2-oxoethyl group with reference to 1D NMR data of **1**. In addition, signals for an ester carbonyl, a tetrasubstituted double bond, a methyl doublet, a methoxy group, four methylenes, two methines including an oxygenated one, and two sp^3^ quaternary carbons were observed in its 1D NMR spectra. Comparison of these data with those of **1** suggested that **2** was similar to **1**, and the main differences was that compound **2** had additional tetrasubstituted double bond and methoxy group. The locations of the double bond (Δ^5,10^) and methoxy group (20-OMe) were confirmed by the HMBC correlations of H_2_-6 to C-5, H_2_-1 to C-10, and 20-OMe to C-20. Its relative stereochemistry was identical to **1** by analysis of the NOE correlations of **2** (Fig. 3). The similar tendency shown in the ECD spectrum of **2** with that of crohalifurane D [1] indicated compound **2** had the (3*R*,8*R*,9*R*) absolute configuration. Therefore, compound **2** was elucidated as shown in Fig. 1.

Crodophylloid C (**3**) (Fig. 1) was deduced to possess a molecular formula of C_21_H_28_O_5_ based on the HR-ESI-MS data. The ^1^H and ^13^C NMR data of **3** (Table 1) had similar signals to **2**, except for the resonances for a double bond. The appearance of a trisubstituted double bond signals in **3** instead of a tetrasubstituted one in **2** suggested the double bonds were in different positions. The double bond was switched to Δ^5^ in **3** confirmed through the observed ^1^H–^1^H COSY cross-peak of H-6/H_2_-7 together with the HMBC correlations of H-6 and H_3_-19 to C-5 (Fig. 2). The relative configuration of **3** was confirmed by analyzing its NOE correlations (Fig. 3). The ECD curve of **3** matched well with that of crodophylloid C (**2**), indicating that a 3*R*,8*R*,9*R*,10*S* absolute configuration was proposed for **3**.

The molecular formula of crodophylloid D (**4**) (Fig. 1) was deduced to be C_21_H_28_O_5_ based on its HR-ESI-MS data together with its ^13^C NMR spectrum. By comparison of the 1D NMR data of **4** (Table 2) with those of the known clerodane diterpenoid 3,12-dioxo-15,16-epoxy-cleroda-13(16),14-dien-9-al [4], it was found that a methoxyl group [*δ*_C_ 51.3 (CH_3_) and 174.2 (C); *δ*_H_ 3.67 (3H, s)] had replaced the aldehyde group (C-20) in the known compound. This was further proven by the HMBC correlations of H_2_-11 and 20-OMe to C-20 (Fig. 2).

**Table 2:** ^1^H and ^13^C NMR Data for Compounds **4**–**6** in CDCl_3_ (*δ* in ppm and *J* in Hz)

Position	**4**^a^		**5**^b^		**6**^a^	
	*δ*_H_, multi. (*J*)	*δ*_C_, type	*δ*_H_, multi. (*J*)	*δ*_C_, type	*δ*_H_, multi. (*J*)	*δ*_C_, type
1	*α* 1.78, m;	25.4, CH_2_	a 1.83, m; b 1.71, m	19.0, CH_2_	a 1.85, m; b 1.72, m	19.2, CH_2_
	*β* 2.20, m					
2	a 2.38, ddd (13.4, 5.2, 2.3); b 2.32, dd (13.4, 6.8)	41.8, CH_2_	*α* 1.63, m;	30.6, CH_2_	a 1.98, m; b 1.52, m	34.6, CH_2_
			*β* 1.83, m			
3		212.4, C	3.82, t (2.9)	71.1, CH	4.32, t (3.0)	74.6, CH
4	2.27, d (6.8)	57.9, CH		79.5, C		159.7, C
5		42.1, C		43.0, C		40.1, C
6	*α* 1.74, dt (13.2, 3.2);	39.2, CH_2_	*α* 1.32, m;	32.3, CH_2_	*α* 1.78, m;	38.3, CH_2_
	*β* 1.35, td (13.2, 4.2)		*β* 1.87, m		*β* 1.55, m	
7	*α* 2.11, qd (13.2, 3.4);	27.8, CH_2_	*α* 2.18, m;	27.2, CH_2_	*α* 2.23, m;	27.6, CH_2_
	*β* 1.43, dq (13.2, 3.6)		*β* 1.31, m		*β* 1.44, m	
8	1.96, m	38.3, CH	1.68, m	41.7, CH	1.78, m	39.6, CH
9		51.9, C		50.2, C		51.4, C
10	2.60, dd (12.7, 2.8)	49.5, CH	2.23, dd (11.9, 2.6)	45.5, CH	1.86, dd (12.3, 2.1)	50.7, CH
11	a 3.27, d (17.0); b 3.22, d (17.0)	43.6, CH_2_	a 3.36, d (16.9); b 2.93, d (16.9)	47.6, CH_2_	a 3.24, d (16.9); b 3.12, d (16.9)	44.5, CH_2_
12		193.4, C		193.6, C		193.3, C
13		129.0, C		128.7, C		128.9, C
14	6.76, dd (1.8, 0.9)	108.8, CH	6.75, dd (1.6, 0.5)	108.9, CH	6.73, dd (1.9, 0.8)	108.8, CH
15	7.45, t (1.8)	144.6, CH	7.42, t (1.6)	144.3, CH	7.42, dd (1.9, 1.3)	144.4, CH
16	8.05, br s	147.2, CH	8.01, br s	146.8, CH	8.01, dd (1.3, 0.8)	147.0, CH
17	1.05, d (6.8)	17.9, CH_3_	1.02, d (6.6)	19.1, CH_3_	1.06, d (6.9)	18.3, CH_3_
18	0.90, d (6.7)	7.2, CH_3_	1.18, s	14.2, CH_3_	a 4.89, s; b 4.86, s	111.0, CH_2_
19	0.64, s	12.5, CH_3_	1.024, s	15.8, CH_3_	1.14, s	20.7, CH_3_
20		174.2, C		175.2, C		174.6, C
4-OMe			3.18, s	50.0, CH_3_		
20-OMe	3.67, s	51.3, CH_3_	3.67, s	51.0, CH_3_	3.69, s	51.2, CH_3_

^a^^1^H NMR measured at 400 MHz and ^13^C NMR at 100 MHz

^b1^H NMR measured at 500 MHz and ^13^C NMR at 125 MHz

**Fig. 4:**
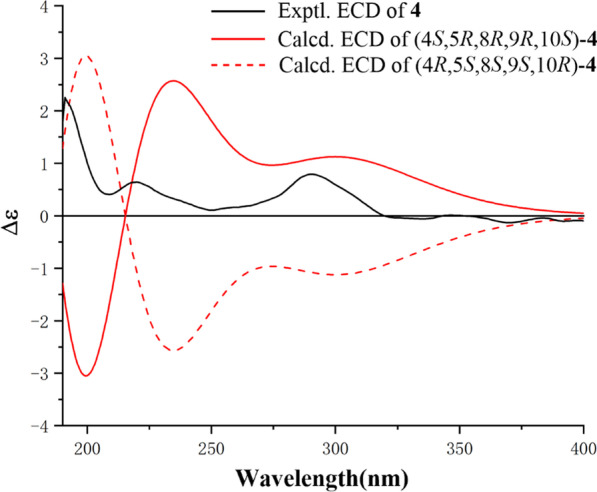
Experimental and calculated ECD spectra of **4**

By comparing their ^1^H and ^13^C NMR data and NOESY correlations, the relative stereochemistry of C-5, C-8, C-9, and C-10 in **4** were determined to be the same as those of 3,12-dioxo-15,16-epoxy-cleroda-13(16),14-dien-9-al. While H-4 was determined as *β*-orientation by the NOESY signals of H-4/H-10 (Fig. 3). The absolute configuration of **4** was assigned via ECD calculation. The ECD curves of this pair of enantiomers, (4*S*,5*R*,8*R*,9*R*,10*S*)-**4** and (4*R*,5*S*,8*S*,9*S*,10*R*)-**4** were simulated by using the TDDFT method. By analysis of the experimental and calculated ECD curves (Fig. 4), the absolute configuration of **4** was determined as 4*S*,5*R*,8*R*,9*R*,10*S*.

Crodophylloid E (**5**) (Fig. 1) possessed the molecular formula C_22_H_32_O_6_ based on the analysis of the HR-ESI-MS data and ^13^C NMR spectrum of **5**. By comparing its ^1^H and ^13^C NMR spectra with those of the known compound 3*α*,4*β*-dihydroxy-15,16-epoxy-12-oxo-cleroda-13(16),14-diene [22], **5** showed an ester carbonyl (*δ*_C_ 175.2) instead of the aldehyde group (C-20) of the known one together with the presence of two extra methoxyls (*δ*_C_ 50.0 and 51.0). These were supported by the HMBC correlations from 4-OMe (*δ*_H_ 3.18) to C-4 (*δ*_C_ 79.5) and from 20-OMe (*δ*_H_ 3.67) to C-20 (*δ*_C_ 175.2) (Fig. 2). The relative stereochemistry of **5** was ascertained the same as the known one [22] on the basis of its NOE correlations (Fig. 3). The 3*R*,4*R*,5*R*,8*R*,9*R,*10*S* absolute configuration of **5** was confirmed by comparing its ECD curve with that of **4**, which showed similar tendency.

Compound **6** (Fig. 1) displayed an HR-ESI-MS ion at *m*/*z* 383.1829 [M + Na]^+^ (calcd 383.1829), suggesting the molecular formula C_21_H_28_O_5_. Comparison of the ^1^H and ^13^C NMR data (Table 1) of **6** with those of crodophylloid E (**5**) suggested that **6** was similar with **5**, with the differences being a Δ^5,18^ exocyclic double bond in **6** instead of the oxygenated quaternary carbon with a methoxy group and the methyl in **5**. These changes were further confirmed via the apparent downfield-shifted carbon chemical shifts of C-4 and C-18 and the HMBC correlations from H_2_-18 to C-4, C-3, and C-5. The relative configurations of C-3, C-5, C-8, C-9, and C-10 in **6** were identical to **5** by the NOESY correlations as shown in Fig. 3. Therefore, compound **6** was determined as illustrated in Fig. 1 and named as crodophylloid F.

Seven known compounds were determined as *ent*-3*α*-hydroxypimara-8(14),15-dien-12-one (**7**) [23], 12-hydroxy-13-methylpodocarpa-8,11,13-trien-3-one (**8**) [24], *epi*-isojatrogrossidione (**9**) [25], 12-hydroxy-13-methyl-*ent*-podocarp-6,8,11,13-tetraen-3-one (**10**) [26], 6-hydroxy-megastigm-7-en-3,9-dione (**11**) [27], (6*S*,7*E*)-6-hydroxy-4,7-megastigmadien-3,9-dione (**12**) [28], and (3*S*,5*R*,6*S*,7*E*)-5,6-epoxy-3-hydroxy-7-megastigmen-9-one (**13**) [27] based on their identical NMR data with the reported.

In the LPS-induced RAW 264.7 inflammatory cell model, the inhibitory effects of all isolates on NO production were tested by the Griess assay. Initially, all the tested compounds at a concentration of 50 μM had no cytotoxicity to RAW 264.7 cells. In comparison to the positive control (Quercetin, IC_50_ = 14.55 ± 0.8 μM), compounds **8** and **9** had certain inhibitory activities (IC_50_ = 19.0 ± 1.8 and 21.6 ± 1.1 μM, respectively), and the remaining terpenoids were inactive (IC_50_ > 50 μM).

## Experimental section

### General experimental procedures

For details see Additional file 1: S1.1.

### Plant material

The plant material was obtained in June 2020 from Xishuangbanna of Yunnan Province, P. R. China, and it was authenticated to be *Croton cnidophyllus* Radcliffe-Smith and Govaerts by Dr. G.H. Tang. A voucher specimen (Accession No.: KCC201907) has been deposited in the School of Pharmaceutical Sciences, SYSU (Sun Yat-sen University).

### Extraction and isolation

The air-dried powder of plant material (15 kg) was soaked in 95% EtOH (50 L × 3) at room temperature for a month. After removing the solvents under vacuum, 800 g of black crude extract was obtained, which was then suspended in water (3 L) and followed by partitioned with ethyl acetate (EtOAc, 3 L × 5). The obtained EtOAc fraction (315 g) was firstly separated over a silica gel column eluted with a gradient of petroleum ether (PE)/EtOAc (50:1 → 1:1) to obtained Frs. Ι–V.

Compounds **1** (1.7 mg) and **2** (2.4 mg) were obtained from Fr. Ι by various column chromatography including silica gel column, Sephadex LH-20 column, and HPLC. **8** (25 mg) and **4** (5 mg, *t*_R_ = 21.5 min) were purified from Fr. ΙΙ, **3** (3 mg), **5** (1 mg), **6** (5 mg),** 7** (11 mg), and **10** (30 mg) from Fr. III, **9** (2 mg) and **11** (8 mg) from Fr. IV, and **12** (8 mg) and **13** (3 mg) from Fr. V by similar separation methods. The detailed separation process can be found in Additional file 1: S1.2.

### Spectroscopic data of compounds

#### Crodophylloid A (1)

Colorless oil; [*α*]_D_^25^ + 12.9 (*c* 0.09, MeCN); UV (MeCN) *λ*_max_ (log *ε*) 190 (3.70) nm; ECD (*c* 5.5 × 10^–4^ M, MeCN) *λ*_max_ (Δ*ε*) 190 (1.27), 217 (1.42) nm; IR (KBr) *ν*_max_ 3444, 2954, 2924, 2854, 1751, 1678, 1156 cm^–1^; 1D NMR data see Table 1; positive ion HR-ESI-MS *m/z* 369.1676 [M + Na]^+^ (calcd for C_20_H_26_O_5_ Na^+^, 369.1672).

#### Crodophylloid B (2)

Colorless oil; [*α*]_D_^25^ + 65.6 (*c* 0.09, MeCN); UV (MeCN) *λ*_max_ (log *ε*) 190 (3.71) nm; ECD (*c* 5.6 × 10^–4^ M, MeCN) *λ*_max_ (Δ*ε*) 190 (1.17), 200 (1.68), 225 (5.80); IR (KBr) *ν*_max_ 3423, 2954, 2924, 2854, 1718 cm^−1^; 1D NMR data see Table 1; positive ion HR-ESI-MS *m/z* 383.1830 [M + Na]^+^ (calcd for C_21_H_28_O_5_Na^+^, 383.1829).

#### Crodophylloid C (3)

Colorless oil; [*α*]_D_^25^ + 11.8 (*c* 0.12, MeCN); UV (MeCN) *λ*_max_ (log *ε*) 190 (4.05) nm; ECD (*c* 5.0 × 10^−4^ M, MeCN) *λ*_max_ (Δ*ε*) 192 (3.16), 208 (1.94), 226 (0.25); IR (KBr) *ν*_max_ 3443, 2925, 1714, 1671, 1156 cm^−1^; 1D NMR data see Table 1; positive ion HR-ESI-MS *m/z* 383.1829 [M + Na]^+^ (calcd for C_21_H_28_O_5_Na^+^, 383.1829).

#### Crodophylloid D (4)

Colorless oil; [*α*]_D_^25^ + 40.3 (*c* 0.07, MeCN); UV (MeCN) *λ*_max_ (log *ε*) 190 (3.83), 250 (3.25) nm; ECD (*c* 4.4 × 10^−4^ M, MeCN) *λ*_max_ (Δ*ε*) 191 (2.25), 220 (0.64), 291 (0.79); IR (KBr) *ν*_max_ 3441, 2953, 2925, 2854, 1712, 1156 cm^−1^; 1D NMR data see Table 2; positive ion HR-ESI-MS *m/z* 383.1830 [M + Na]^+^ (calcd for C_21_H_28_O_5_ Na^+^, 383.1829).

#### Crodophylloid E (5)

Colorless oil; [*α*]_D_^25^ + 7.6 (*c* 0.12, MeCN); UV (MeCN) *λ*_max_ (log *ε*) 190 (3.32) nm; ECD (*c* 8.9 × 10^−4^ M, MeCN) *λ*_max_ (Δ*ε*) 194 (+ 0.54), 220 (+ 0.42); IR (KBr) *ν*_max_ 3445, 2917, 2849, 1708, 1462, 1155 cm^−1^; 1D NMR data see Table 2; positive ion HR-ESI-MS *m/z* 415.2092 [M + Na]^+^ (calcd for C_22_H_32_O_6_Na^+^, 415.2091).

#### Crodophylloid F (6)

Colorless oil; [*α*]_D_^25^ + 16.8 (*c* 0.11, MeCN); UV (MeCN) *λ*_max_ (log *ε*) 190 (3.95); ECD (*c* 5.5 × 10^−4^ M, MeCN) *λ*_max_ (Δ*ε*) 191 (0.91), 200 (2.87), 225 (0.05); IR (KBr) *ν*_max_ 3443, 2923, 1723, 1155 cm^−1^; 1D NMR data see Table 2; positive ion HR-ESI-MS *m/z* 383.1829 [M + Na]^+^ (calcd for C_21_H_28_O_5_Na^+^, 383.1829).

### ECD calculations

For details of the quantum chemical ECD calculation of **4**, see Additional file 1: S1.5.

### Anti-inflammatory activity assay

RAW 264.7 cells were obtained from Southern Medical University Cell Bank (Guangzhou, China). The cell viability and the NO concentration were evaluated by the MTT assay and the Griess reaction, respectively (for details see Additional file 1: S1.3 and S1.4).

## Supplementary Information

**Additional file 1.** The NMR, ECD, and HSMS spectra of **1**–**6**, ^13^C NMR spectroscopic data for **7**–**13**, general experimental procedures, extraction and isolation, and ECD calculation for **4**.

## Data Availability

All data generated and analyzed during this study are included in this published article and its Additional file 1.
