# Supplementary material for: New halimane and clerodane diterpenoids from Croton cnidophyllus
Source: Nat Prod Bioprospect. 2023 Jul 6;13(1):21. doi: 10.1007/s13659-023-00386-z (PMC10326188; doi:10.1007/s13659-023-00386-z)
Supplement: Supplementary file 1 — Additional file 1. The NMR, ECD, and HSMS spectra of 1–6, 13C NMR spectroscopic data for 7–13, general experimental procedures, extraction and isolation, and ECD calculation for 4. [file 13659_2023_386_MOESM1_ESM.pdf]

Supplementary Information

New halimane and clerodane diterpenoids from *Croton cnidophyllus*

Xun Wei, Jia-Luo Huang, Hua-Hua Gao, Fang-Yu Yuan, Gui-Hua Tang* and Sheng Yin*

School of Pharmaceutical Sciences, Sun Yat-sen University, Guangzhou 510006, People's Republic of China

*Correspondence: tanggh5@mail.sysu.edu.cn (G-H Tang); yinsh2@mail.sysu.edu.cn (S Yin)

Contents

S1. Experimental Section······P4–P9

S1.1 General experimental procedures······P4

S1.2. Extraction and isolation······P4–P5

S1.3. MTT assay······P5

S1.4. Griess assay······P5–P6

S1.5. ECD calculations······P6–P11

S1.5.1. ECD calculations of compound **4** ······P6–P7

S1.5.2. Conformers and energy analysis······P7

S1.5.3. ECD data of compound **4**······P7–P9

S2. Spectroscopic Data Listed in Tables or Shown in Figures······P10–P14

**Table S2.1.** The ^{13}C NMR (100 Hz) data of compounds **7–10** in $CDCl_3$ (δ in ppm)	P10
**Table S2.2.** The ^{13}C NMR (100 Hz) data of compounds **11–13** in $CDCl_3$ (δ in ppm)	P11
**Figure S2.1.** ECD spectrum of compound **1**	P11
**Figure S2.2.** ECD spectrum of compound **2**	P12
**Figure S2.3.** ECD spectrum of compound **3**	P12
**Figure S2.4.** ECD spectrum of compound **5**	P13
**Figure S2.5.** ECD spectrum of compound **6**	P13

S3. 1D and 2D NMR Spectra of 1–6 ······ P14–P31

S3.1. ^{1}H NMR spectrum of compound **1** ······ P14

S3.2. ^{13}C NMR and DEPT spectra of compound **1** ······ P14

S3.3. HSQC spectrum of compound **1** ······ P15

S3.4. ^{1}H–^{1}H COSY spectrum of compound **1** ······ P15

S3.5. HMBC spectrum of compound **1** ······ P16

S3.6. NOESY spectrum of compound **1** ······ P16

S3.7. ^{1}H NMR spectrum of compound **2** ······ P17

S3.8. ^{13}C NMR and DEPT spectra of compound **2** ······ P17

S3.9. HSQC spectrum of compound **2** ······ P18

S3.10. ^{1}H–^{1}H COSY spectrum of compound **2** ······ P18

S3.11. HMBC spectrum of compound **2** ······ P19

S3.12. NOESY spectrum of compound **2** ······ P19

S3.13. ^{1}H NMR spectrum of compound **3** ······ P20

S3.14. ^{13}C NMR and DEPT spectra of compound **3** ······ P20

S3.15. HSQC spectrum of compound **3** ······ P21

S3.16. ^{1}H–^{1}H COSY spectrum of compound **3** ······ P21

S3.17. HMBC spectrum of compound **3** ······ P22

S3.18. NOESY spectrum of compound **3** ······ P22

S3.19. ^{1}H NMR spectrum of compound **4** ······ P23

S3.20. ^{13}C NMR and DEPT spectra of compound **4** ······ P23

S3.21. HSQC spectrum of compound **4** ······ P24

S3.22. ^{1}H–^{1}H COSY spectrum of compound **4** ······ P24

S3.23. HMBC spectrum of compound **4** ······ P25

S3.24. NOESY spectrum of compound **4** ······ P25

S3.25. ^{1}H NMR spectrum of compound **5** ······ P26

S3.26. ^{13}C NMR and DEPT spectra of compound **5** ······ P26

S3.27. HSQC spectrum of compound **5** ······ P27

S3.28. ^{1}H–^{1}H COSY spectrum of compound **5** ······ P27

S3.29. HMBC spectrum of compound **5** ······ P28

S3.30. NOESY spectrum of compound **5** ······ P28

S3.31. ^{1}H NMR spectrum of compound **6**······P29

S3.32. ^{13}C NMR and DEPT spectra of compound **6**······P29

S3.33. HSQC spectrum of compound **6**······P30

S3.34. ^{1}H–^{1}H COSY spectrum of compound **6**······P30

S3.35. HMBC spectrum of compound **6**······P31

S3.36. NOESY spectrum of compound **6**······P31

S4. HRMS Spectra of New Compounds 1–6 ······P32−P34

S4.1. HRESIMS data of compound **1**······P32

S4.2. HRESIMS data of compound **2**······P32

S4.3. HRESIMS data of compound **3**······P33

S4.4. HRESIMS data of compound **4**······P33

S4.5. HRESIMS data of compound **5**······P34

S4.6. HRESIMS data of compound **6**······P34

S1. Experimental Section

S1.1 General experimental procedures

Optical rotations were measured on an AntonPaar MCP200 modular circular polarimeter. UV and ECD spectra were recorded on an Applied Photophysics Chirascan spectrometer. IR spectra were obtained from a Bruker Tensor 37 infrared spectrometer. 1D and 2D NMR spectra were collected from Bruker Ascend TM 500 and Bruker Avance III 400spectrometers at 25°C. HR-ESI-MS data were acquired via a Waters Micromass Q-TOF spectrometer. Semipreparative HPLC was carried out on a Shimadzu LC-20 AT equipped with an SPD-M20A PDA detector. A NanoChrom ChromCoreTM 5-120 C_{18} column (250 × 10 mm, 5 μm), a Phenomenex Lux cellulose-2 chiral-phase column (250 × 10 mm, 5 μm), and a YMC-pack ODS-A column (250 × 10 mm, S-5 μm, 12 nm) were utilized for HPLC purification. Solvents MeCN for HPLC were purchased from BCR International Trading Co. Ltd. Reversed-phase C_{18} (Rp-C_{18}) silica gel from YMC Co. Ltd. (12 nm, S-50 μm), MCI gel from Mitsubishi Chemical Industries Ltd. (CHP20P, 75–150 μm), Sephadex LH-20 gel from Amersham Biosciences, and silica gel from Qingdao Haiyang Chemical Co, Ltd. (100–200, 300–400 mesh,) were utilized for general column chromatographic separation, and fractions were monitored via silica gel TLC (GF254 plates, 0.25 mm thickness) visualized with 15% sulfuric acid in EtOH.

S1.2 Extraction and isolation

The air-dried powder of plant material (15 kg) was soaked in 95% EtOH (50 L × 3) at room temperature for a month. After removing the solvents under vacuum, 800 g of black crude extract was obtained, which was then suspended in water (3 L) and followed by partitioned with ethyl acetate (EtOAc, 3 L × 5). The obtained EtOAc fraction (315 g) was firstly separated over a silica gel column eluted with a gradient of petroleum ether (PE)/EtOAc (50:1 → 1:1) to obtained Frs. I–V.

Fr. I was subjected to a silica gel column (PE/EtOAc, 200:1 → 50:1) to give Frs. Ia–Ic. Fr. Ic (300 mg) was chromatographed over a Sephadex LH-20 column (MeOH) and subsequently subjected to semipreparative HPLC (YMC-pack ODS-A column, MeCN/H_2O, 6:4, 3 mL/min) to give compounds **1** (1.7 mg, t_R = 11.5 min) and **2** (2.4 mg, t_R = 13.5 min).

Separation of Fr. II by a Rp-C_{18} silica gel column (MeOH/H_2O, 4:6 → 100:0), followed by a silica gel column (CH_2Cl_2/MeOH, 500:1 → 15:1) to give **8** (25 mg) and Fr. IIa (36 mg). Then compound **4** (5 mg, t_R = 21.5 min) was obtained from Fr. IIa by semipreparative chiral-phase HPLC (MeCN/H_2O, 60:40, 3 mL/min).

Fr. III was subjected to an MCI gel column (MeOH/H_2O, 4:6 → 100:0) to give Frs. IIIa–IIIc. Fr. IIIa was chromatographed over a Sephadex LH-20 column (MeOH) and subsequently subjected to semipreparative chiral-phase HPLC to obtain **3** (3 mg, t_R = 17.5 min) and **6** (5 mg, t_R = 23 min). Fr. IIIb1 (16 mg) and Fr. IIIb2 (50 mg) were obtained from Fr. IIIb by a Sephadex LH-20 column (MeOH). Compound **5** (1 mg, t_R = 13.5 min) was purified from Fr. IIIb1 by semipreparative HPLC with a chiral column (MeCN/H_2O, 6:4, 3 mL/min), and compound **10** (30 mg) was purified from Fr. IIIb2 by a Sephadex LH-20 column (MeOH). Fr. IIIc (242 mg) was separated by a Sephadex LH-20 column followed by semipreparative chiral-phase HPLC (MeCN/H_2O, 6.5:3.5, 3 mL/min) to afford **7** (11 mg, t_R = 23 min).

Fr. IV was subjected to a silica gel column (CH_2Cl_2/MeOH, 1:0 → 50:1) and successively separated via an MCI gel column (MeOH/H_2O, 50%–100%) to give Frs. IVa and IVb. Compound **11** (8 mg, t_R = 10 min) was purified from Fr. IVa (60 mg) by semipreparative HPLC with a chiral column (MeCN/H_2O, 3.5:6.5, 3 mL/min), and **9** (2 mg, t_R = 10 min) was from Fr. IVb (40 mg) by semipreparative HPLC with a NanoChrom ChromCoreTM 5-120 C_{18} column (MeCN/H_2O, 5.5:4.5, 3 mL/min).

Fr. V was subjected to a silica gel column (CH_2Cl_2/MeOH, 500:1 → 50:1) to yield Frs. Va and Vb. Further purification of Fr. Va (1.37 g) by an MCI gel column (MeOH/H_2O, 4:6 → 100:0), a Sephadex LH-20 column (MeOH), and chiral-phase HPLC (MeCN/H_2O, 2.5:7.5, 3 mL/min) led to compounds **12** (8 mg, t_R = 12 min) and **13** (3 mg, t_R = 14 min).

S1.3 MTT assay

Briefly, RAW 264.7 cells were incubated with 20 μL MTT solution (0.5 mg/mL in medium) for 4 h at 37 °C, and then the supernatants were removed and residues were dissolved in 100 μL DMSO. The absorbance was detected at 570 nm using a microplate reader (Molecular Devices, USA) and analyzed using a Softmax Pro 5 software (Molecular Devices, USA).

S1.4 Griess assay

RAW 264.7 cells were plated into a 96-well plate (4×10^4 cells/well). After 24 h, they were pretreated with compounds for 30 min and then stimulated with 1 μg/mL LPS treated for 24 h. After that, 50 μL of culture supernatant was allowed to react with 100 μL of Griess reagent (1% sulfanilamide, 0.1% *N*-1-naphthylethylenediamine dihydrochloride in 5% phosphoric acid) for 10 min at rt in the dark. Then, the optical density (100 μL per well) was measured at 540 nm using a microplate reader (Molecular Devices, USA). Sodium nitrite was used as a standard to calculate the nitrite concentration. Inhibition (%) = $(1 - (A_{LPS+sample} - A_{untreated}) / (A_{LPS} - A_{untreated})) \times 100$. The experiments were performed in

triplicates, and the data were expressed as the mean ± standard deviation (SD) values. Quercetin was used as a positive control.

S1.5. ECD calculations

S1.5.1 ECD calculations of compound 4

The absolute configurations of **4** were determined by quantum chemical calculations of their theoretical ECD spectra. One of the two enantiomers for each compound, (4*S*,5*R*,8*R*,9*R*,10*S*)-**4** was arbitrary chosen for theoretical studies. Conformational analyses were first carried out via Monte Carlo searching using molecular mechanism with MMFF force field in the *Spartan 18* program.[1] The results showed eight lowest energy conformers for **4** within an energy window of 2.5 Kcal/mol. These conformers were reoptimized using DFT at the B3LYP/6-31G(d) level in gas phase using the Gaussian 09 program.[2] Four conformers of **4** (Fig. S1.1) whose relative Gibbs free energies in the range of 0−1.5 Kcal/mol were refined and considered for next step. All the reoptimized conformers mentioned above for **4** were applied for theoretical ECD calculation. The energies, oscillator strengths, and rotational strengths of the first 30 electronic excitations were calculated using the TD-DFT methodology at the M062X/TZVP level in PCM (acetonitrile). The ECD spectra were simulated by the overlapping Gaussian function (σ = 0.50 eV)[3]. To get the final ECD spectrum of each compound, the simulated spectra of the lowest energy conformers were averaged according to the Boltzmann distribution theory and their relative Gibbs free energy (ΔG). The theoretical ECD curve of (4*R*,5*S*,8*S*,9*S*,10*R*)-**4** was obtained by directly reverse that of (4*S*,5*R*,8*R*,9*R*,10*S*)-**4**.

References

[1] *Spartan 18*; Wavefunction Inc.:Irvine, CA.

[2] *Gaussian 09*, Revision A.1, Frisch, M. J.; Trucks, G. W.; Schlegel, H. B.; Scuseria, G. E.; Robb, M. A.; Cheeseman, J. R.; Scalmani, G.; Barone, V.; Mennucci, B.; Petersson, G. A.; Nakatsuji, H.; Caricato, M.; Li, X.; Hratchian, H. P.; Izmaylov, A. F.; Bloino, J.; Zheng, G.; Sonnenberg, J. L.; Hada, M.; Ehara, M.; Toyota, K.; Fukuda, R.; Hasegawa, J.; Ishida, M.; Nakajima, T.; Honda, Y.; Kitao, O.; Nakai, H.; Vreven, T.; Montgomery, Jr., J. A.; Peralta, J. E.; Ogliaro, F.; Bearpark, M.; Heyd, J. J.; Brothers, E.; Kudin, K. N.; Staroverov, V. N.; Kobayashi, R.; Normand, J.; Raghavachari, K.; Rendell, A.; Burant, J. C.; Iyengar, S. S.; Tomasi, J.; Cossi, M.; Rega, N.; Millam, J. M.; Klene, M.; Knox, J. E.; Cross, J. B.; Bakken, V.; Adamo, C.; Jaramillo, J.; Gomperts, R.; Stratmann, R. E.; Yazyev, O.; Austin, A. J.; Cammi, R.; Pomelli, C.; Ochterski, J. W.; Martin, R. L.; Morokuma, K.; Zakrzewski, V. G.; Voth,

G. A.; Salvador, P.; Dannenberg, J. J.; Dapprich, S.; Daniels, A. D.; Farkas, Ö.; Foresman, J. B.; Ortiz, J. V.; Cioslowski, J.; Fox, D. J. Gaussian, Inc., Wallingford CT, 2009.

[3] Grimblat, N.; Zanardi, M. M.; Sarotti, A. M. Beyond DP4: an Improved Probability for the Stereochemical Assignment of Isomeric Compounds using Quantum Chemical Calculations of NMR Shifts. *J. Org. Chem.* **2015**, *80*, 12526−12534.

S1.5.2. Conformers and energy analysis

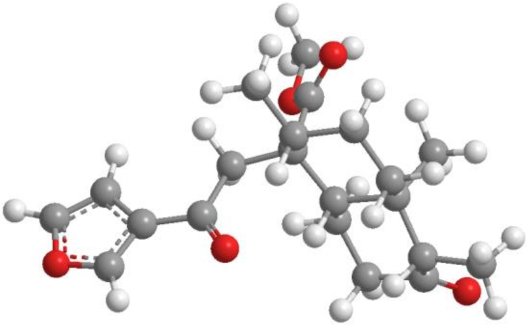

4C1

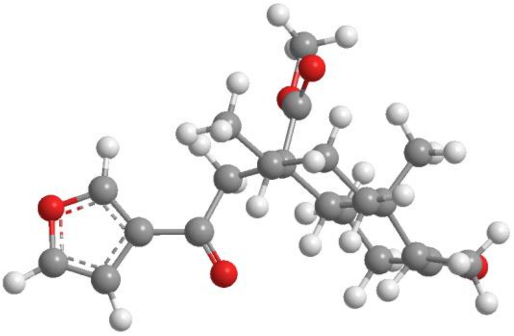

4C2

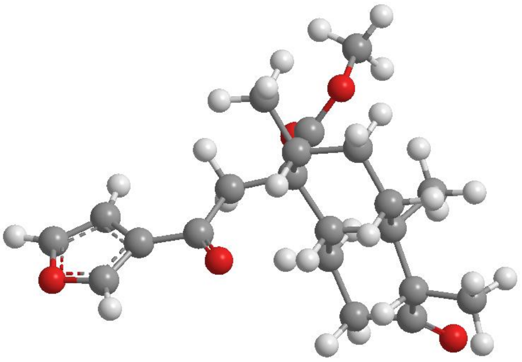

4C3

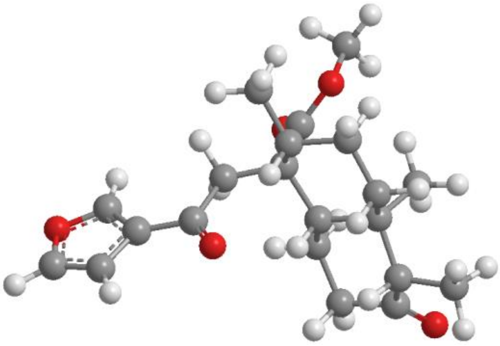

4C4

Fig. S1.1 B3LYP/6-31G(d) optimized lowest energy conformers for **4**

Table S1.1 Energy (298.15 K) analysis for **4**

Conf.	G (Hartree)	ΔG (Kcal/mol)	Boltzmann Distribution
**4C1**	-1192.792607	0.58609434	0.202571253
**4C2**	-1192.793541	0	0.545013372
**4C3**	-1192.79173	1.13642061	0.079979747
**4C4**	-1192.792455	0.68147586	0.172435627

S1.5.3 ECD data of compound 4

ECD spectrum of each conformation is simulated according to the overlapping Gaussian functions expressed as:

$$\Delta\varepsilon(E)=\frac{1}{2.296\times10^{-39}\sqrt{\pi}\sigma}\sum_{i}^{A}\Delta E_i R_i e^{[-(E-\Delta E_i)^2/\sigma^2]}$$

Where σ is half the bandwidth at 1/e peak height and expressed in energy units. The parameters ΔE_i and R_i are the excitation energies and rotational strengths for the transition *i*, respectively.

The above function is converted to Δε, λ (wavelength) correlations as:

$$\Delta\varepsilon(\lambda)=\frac{1}{2.296\times10^{-39}\sqrt{\pi}\sigma}\sum_{i}^{A}\Delta E_i R_i e^{[-(1240/\lambda-\Delta E_i)^2/\sigma^2]}$$

and then simulation was accomplished by using the Excel 2003 and the Origin 9.1 software.

To get the final spectra, all the simulated spectra of conformations of each compound were averaged according to their energy and the Boltzmann distribution theory expressed as:

$$\frac{N_i^*}{N}=\frac{g_i e^{-\varepsilon_i/k_B T}}{\sum g_i e^{-\varepsilon_i/k_B T}}$$

Table S1.2 Calculated ECD data for **4** in PCM (acetonitrile).

State	**4C1**		**4C2**		**4C3**		**4C4**	
	Excitation energies(eV)	Rotatory Strengths*	Excitation energies(eV)	Rotatory Strengths*	Excitation energies(eV)	Rotatory Strengths*	Excitation energies(eV)	Rotatory Strengths*
1	3.9703	0.102	3.9444	2.7174	3.9827	-0.7245	4.0038	-0.0165
2	4.1456	4.8184	4.1451	4.4623	4.1468	4.925	4.1466	5.1739
3	5.1653	6.9601	5.258	7.0484	5.1683	7.265	5.2507	-6.0809
4	5.4311	4.2005	5.4363	11.4502	5.5565	2.8868	5.5568	0.0872
5	6.2817	-7.4825	6.2139	-16.8074	6.2833	-7.5002	6.255	1.1608
6	6.5314	-7.1644	6.4579	-10.1543	6.5398	-6.3394	6.5224	-2.6902
7	6.6606	2.5014	6.6616	17.6253	6.658	1.33	6.7122	6.3559
8	6.893	-3.319	6.7798	-2.9714	6.915	0.8937	6.8951	0.4775

9	7.1187	-4.6981	7.0316	-11.2803	7.1102	-4.8587	7.0777	-4.3738
10	7.1721	1.5832	7.0746	9.7002	7.2209	1.2435	7.1959	-0.2511
11	7.259	16.5122	7.2757	10.745	7.2685	9.9033	7.2654	3.4342
12	7.3039	9.4741	7.3162	9.9576	7.3112	14.7486	7.3102	11.7772
13	7.4992	-0.4759	7.5163	10.007	7.4766	0.663	7.5002	9.3308
14	7.5101	4.9746	7.5314	1.7671	7.5113	14.0192	7.5206	-0.0521
15	7.5519	18.3457	7.6086	-0.1261	7.5417	1.6921	7.5534	-6.0472
16	7.6086	-6.5998	7.6531	-62.6058	7.6364	-25.3013	7.6408	-10.149
17	7.674	-15.134	7.707	-19.8534	7.6476	-2.0779	7.6865	-6.2807
18	7.7004	-37.3177	7.7146	21.4513	7.6942	-6.828	7.7091	-17.6896
19	7.7182	-7.2385	7.7441	-1.6948	7.7207	9.5262	7.7597	-3.2644
20	7.7855	37.734	7.7585	8.3572	7.8238	-0.4257	7.8302	20.7661
21	7.8326	-29.771	7.8255	27.6869	7.8636	6.9788	7.8352	16.4301
22	7.8843	60.9712	7.8977	-13.3742	7.8777	49.9897	7.8787	46.5446
23	7.8878	17.3185	7.9072	66.7907	7.9002	-11.5006	7.9051	-26.4222
24	7.9067	-1.386	7.9378	32.9583	7.912	55.9029	7.9306	-30.4191
25	7.9482	-21.7894	7.9652	24.5912	7.946	30.5837	7.9505	36.6752
26	7.9606	-4.263	7.9968	-41.1674	7.9509	-81.974	7.9863	53.1095
27	8.0741	-3.8946	8.0691	-18.0196	8.0862	-1.3368	8.0662	1.765
28	8.1819	3.7182	8.1211	-11.2214	8.1025	2.5587	8.0933	1.7612
29	8.2265	-6.92	8.1525	-6.4105	8.2386	2.9682	8.2435	8.0102
30	8.2518	0.0482	8.2211	-4.739	8.2953	-8.8088	8.2823	-3.3476

* R(velocity) 10**-40 erg-esu-cm

S2. Spectroscopic data listed in Tables or shown in Figures

Table S2.1 ^{13}C (100 MHz) NMR Data for Compounds **7–10** (δ in ppm)

position	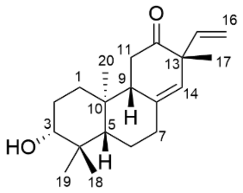 **7**	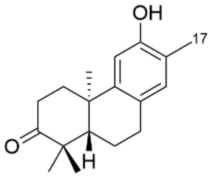 **8**	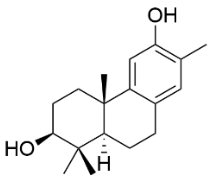 **9**	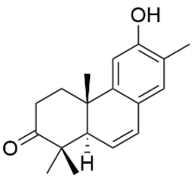 **10**
	δ_C[a], Type	δ_C[a], Type	δ_C[b], Type	δ_C[a], Type
1	36.8, CH_2	37.7, CH_2	38.5, CH_2	35.3, CH_2
2	27.5, CH_2	34.8, CH_2	28.8, CH_2	34.7, CH_2
3	78.9, CH	218.2, C	79.5, CH	215.9, C
4	39.1, C	47.5, C	40.1, C	47.3, C
5	53.4, CH	50.7, CH	51.6, CH	51.7, CH
6	22.2, CH_2	20.5, CH_2	20.3, CH_2	125.6, CH
7	34.9, CH_2	30.0, CH_2	31.0, CH_2	128.6, CH
8	138.4, C	126.8, C	126.7, C	125.8, C
9	54.2, CH	146.3, C	122.8, C	145.3, C
10	38.8, C	37.2, C	38.5, C	37.5, C
11	35.3, CH_2	111.6, CH	111.3, CH	109.5, CH
12	213.1, C	152.4, C	149.1, C	153.9, C
13	52.3, C	122.1, C	154.3, C	121.2, C
14	126.5, CH	131.4, CH	131.9, CH	129.6, CH
15	142.9, CH			
16	115.6, CH_2			
17	23.5, CH_3	15.5, CH_3	16.2, CH_3	15.3, CH_3
18	15.8, CH_3	24.7, CH_3	15.7, CH_3	22.8, CH_3
19	28.5, CH_3	27.0, CH_3	28.8, CH_3	25.0, CH_3
20	14.6, CH_3	21.2, CH_3	25.4, CH_3	20.1, CH_3

[a] Measured in $CDCl_3$; [b] Measured in MeOD-d_4

Table S2.2 ^{13}C (100 MHz) NMR Data for Compounds **11–13** (δ in ppm)

position	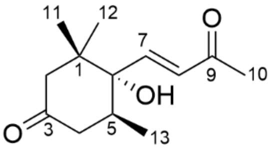 **11**	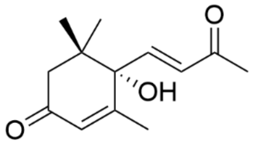 **12**	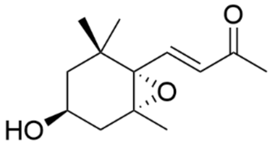 **13**
	δ_C[a], Type	δ_C[b], Type	δ_C[a], Type
1	44.2, C	41.6, C	35.3, C
2	52.4, CH_2	49.7, CH_2	40.7, CH_2
3	213.8, C	197.6, C	64.1, CH
4	45.9, CH_2	127.9, CH	46.8, CH_2
5	37.6, CH	160.5, C	67.4, C
6	78.7, C	79.4, C	69.6, C
7	152.3, CH	145.1, CH	142.5, CH
8	132.4, CH	130.5, CH	132.7, CH
9	200.6, C	197.1, C	197.6, C
10	27.4, CH_3	28.6, CH_3	28.4, CH_3
11	25.1, CH_3	23.1, CH_3	25.1, CH_3
12	25.1, CH_3	24.5, CH_3	29.5, CH_3
13	16.4, CH_3	18.8, CH_3	20.0, CH_3

[a] Measured in $CDCl_3$; [b] Measured in MeOD-d_4

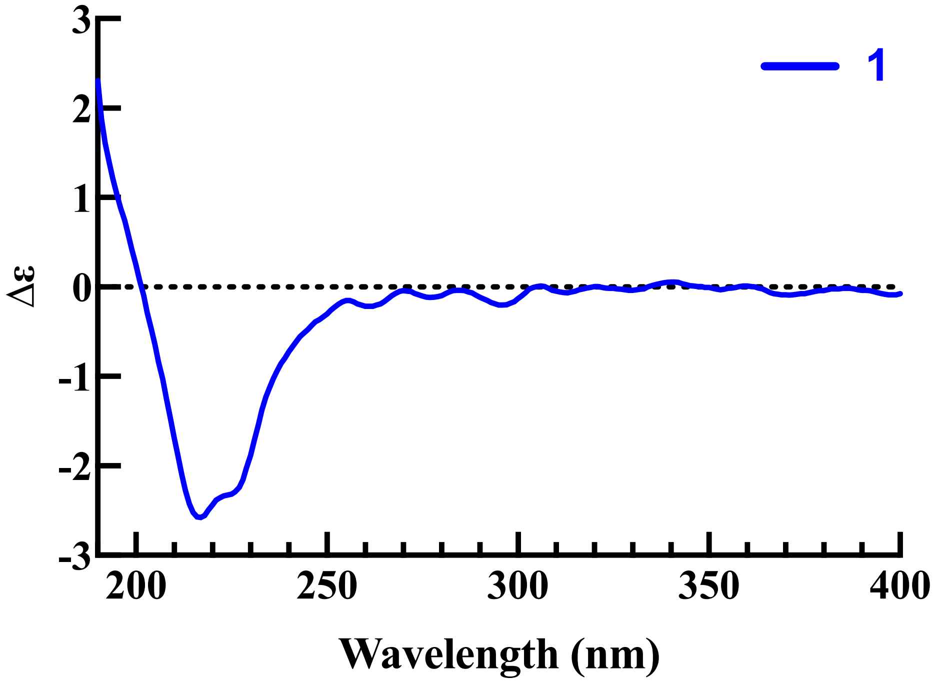

Fig. S2.1 ECD spectrum of compound **1**.

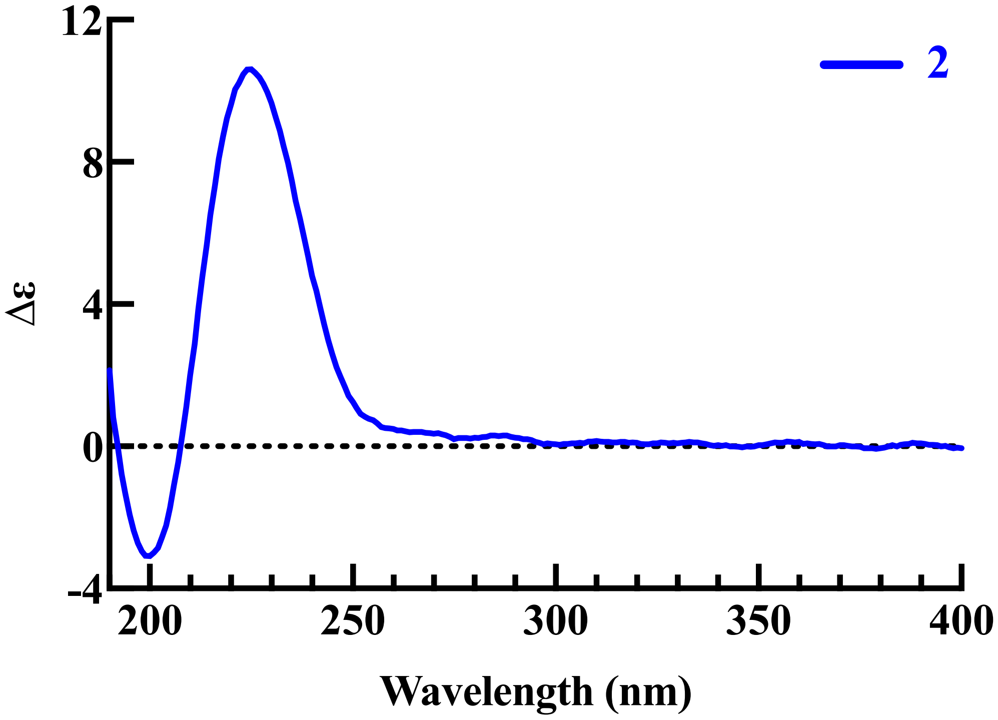

Fig. S2.2 ECD spectrum of compound **2**.

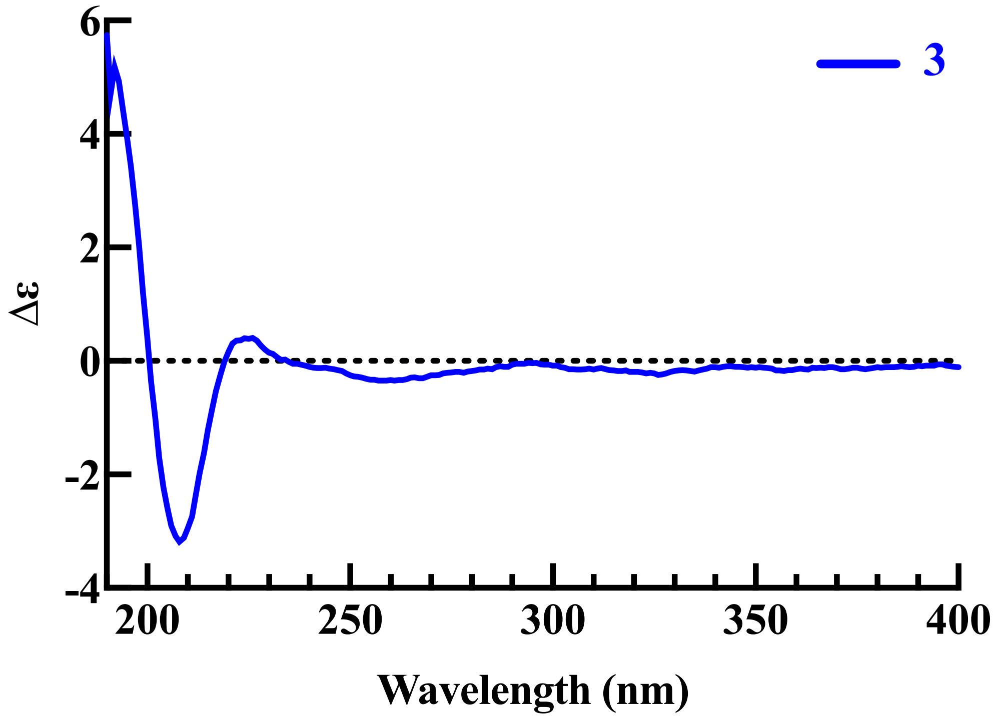

Fig. S2.3 ECD spectrum of compound **3**.

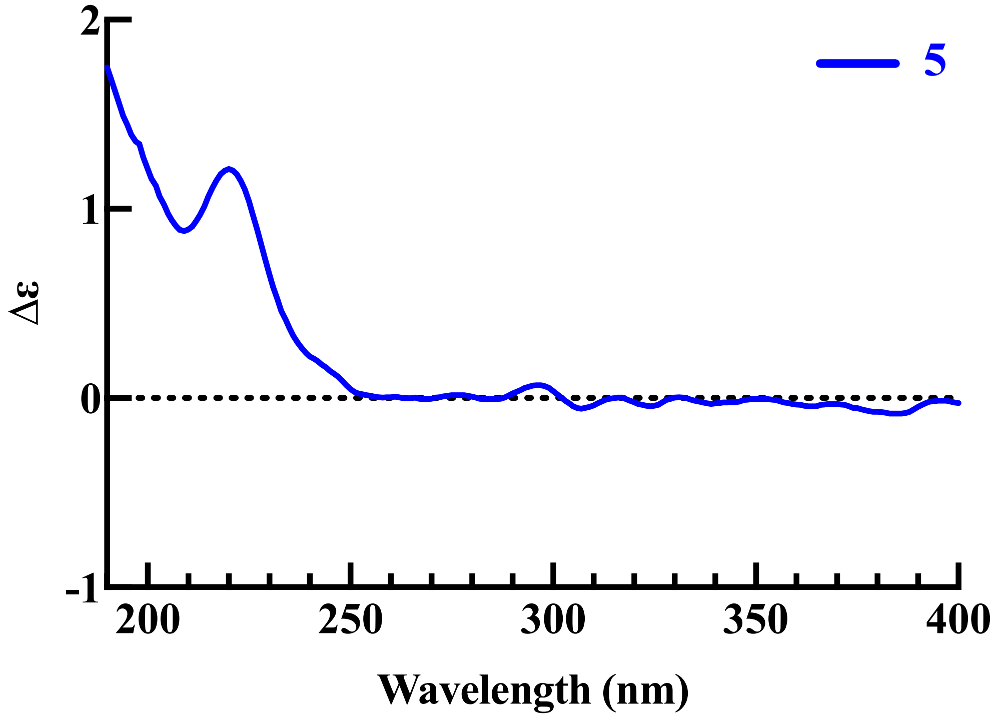

Fig. S2.4 ECD spectrum of compound **5**.

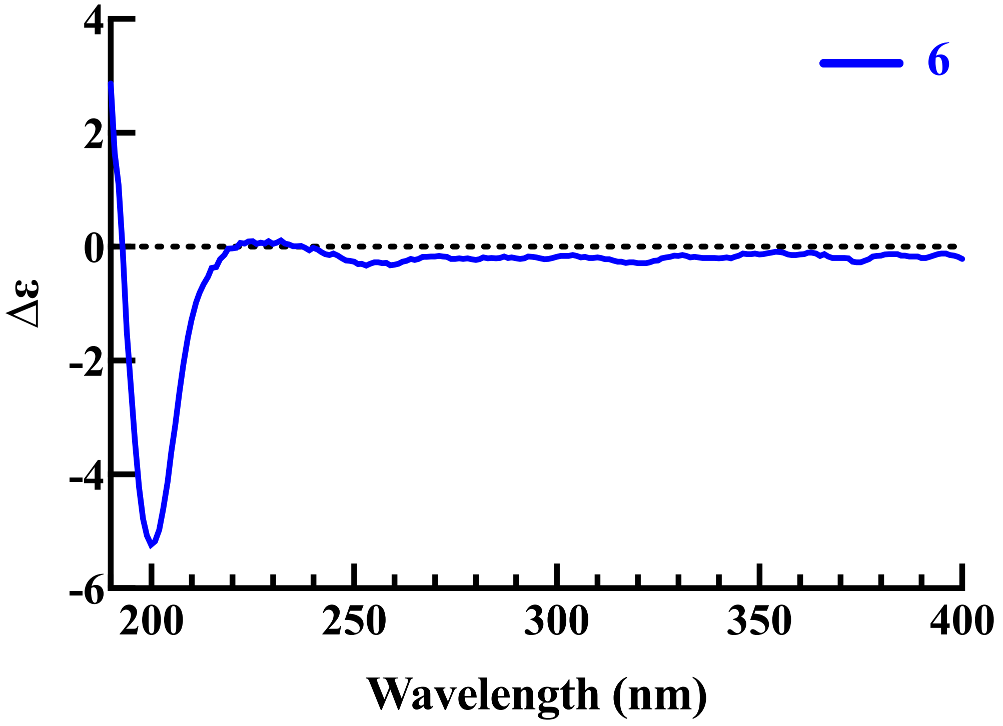

Fig S2.5 ECD spectrum of compound **6**.

S3. 1D and 2D NMR spectra of **1–6**.

S3.1. ^{1}H NMR spectrum of compound **1**.

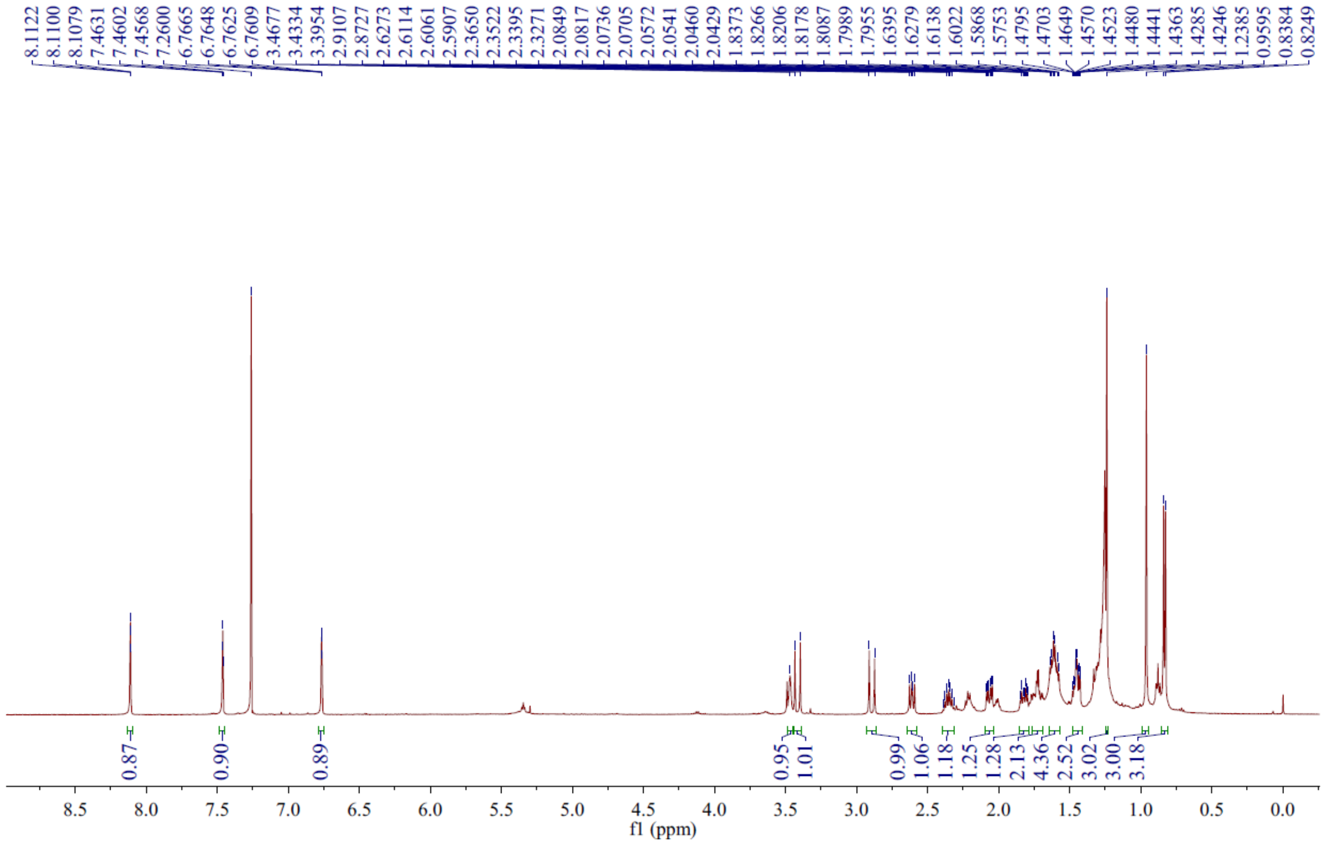

S3.2. ^{13}C NMR and DEPT spectra of compound **1**.

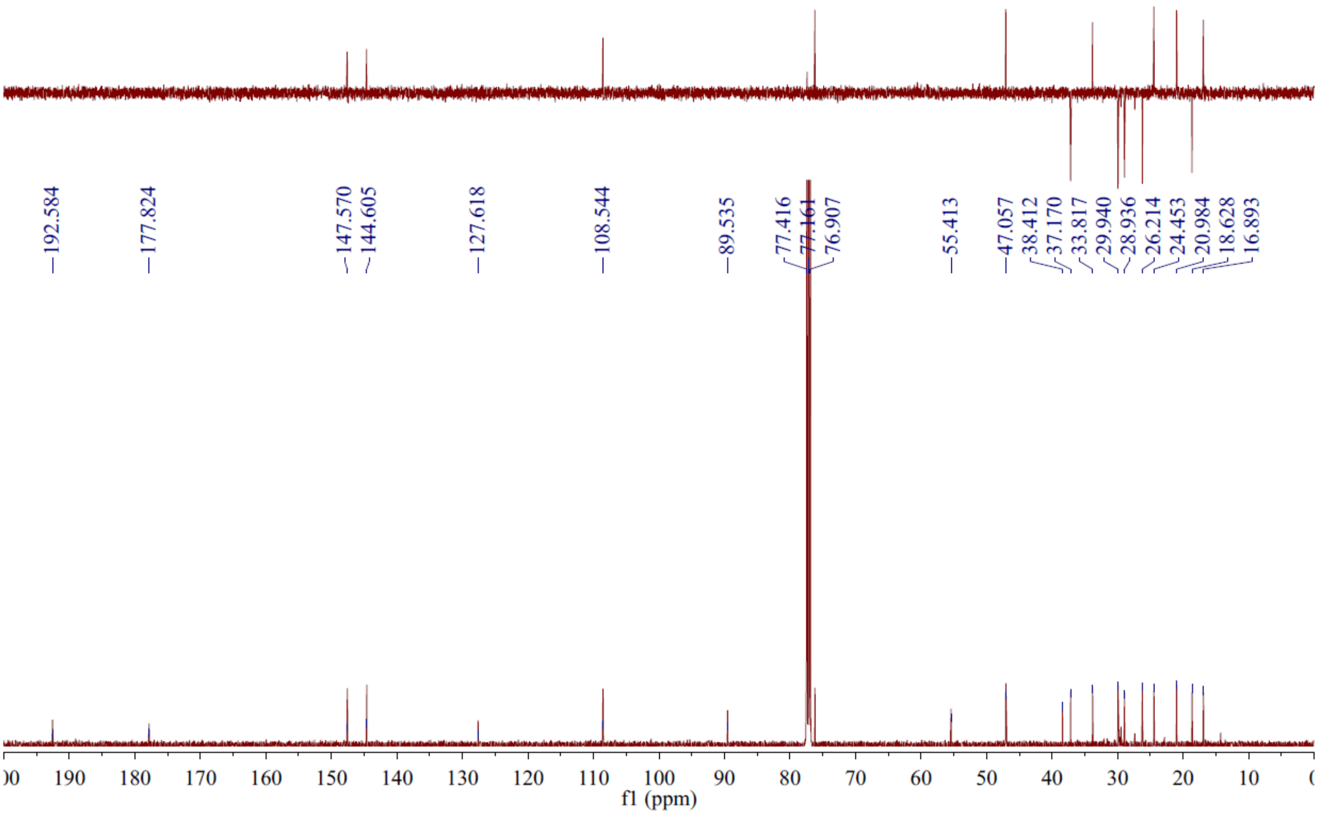

S3.3. HSQC spectrum of compound **1**.

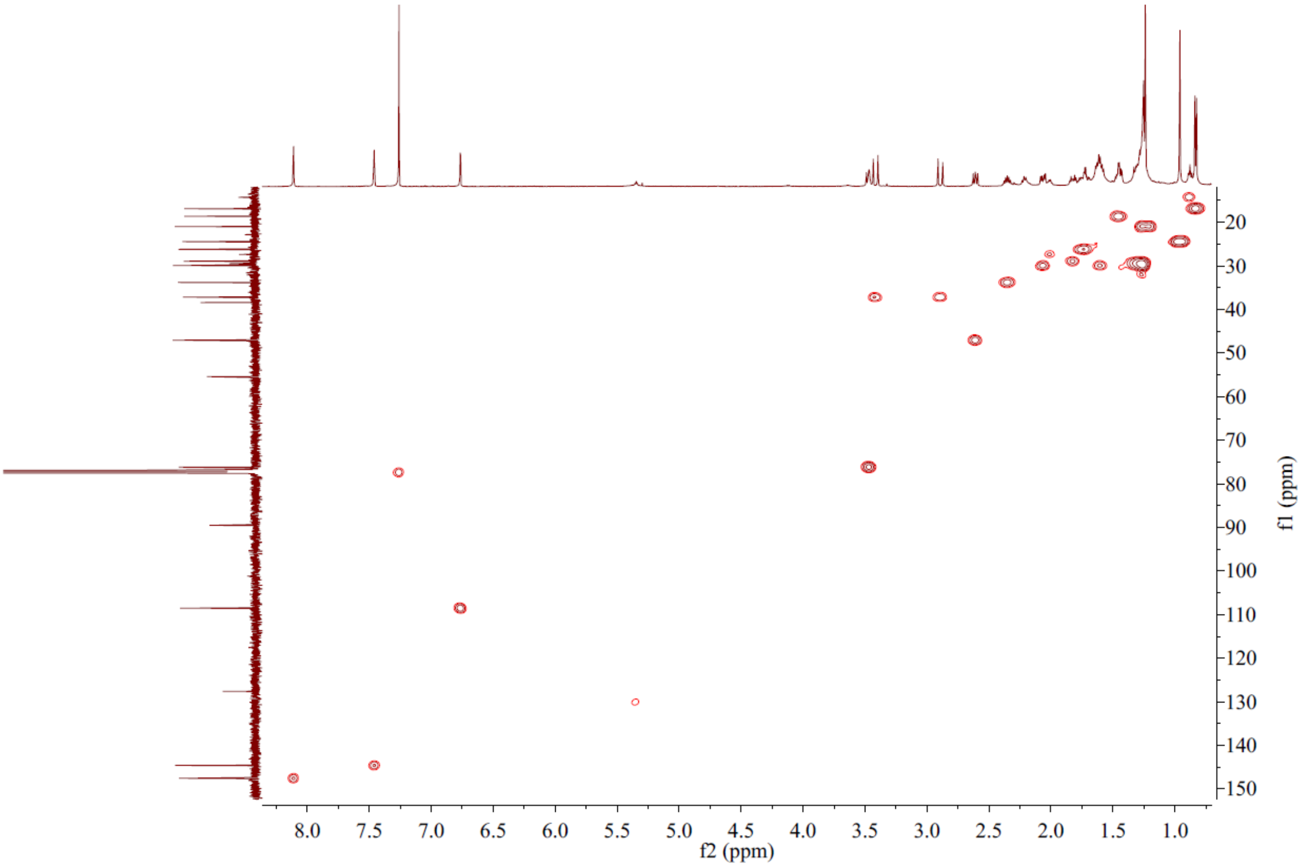

S3.4. ^{1}H–^{1}H COSY spectrum of compound **1**.

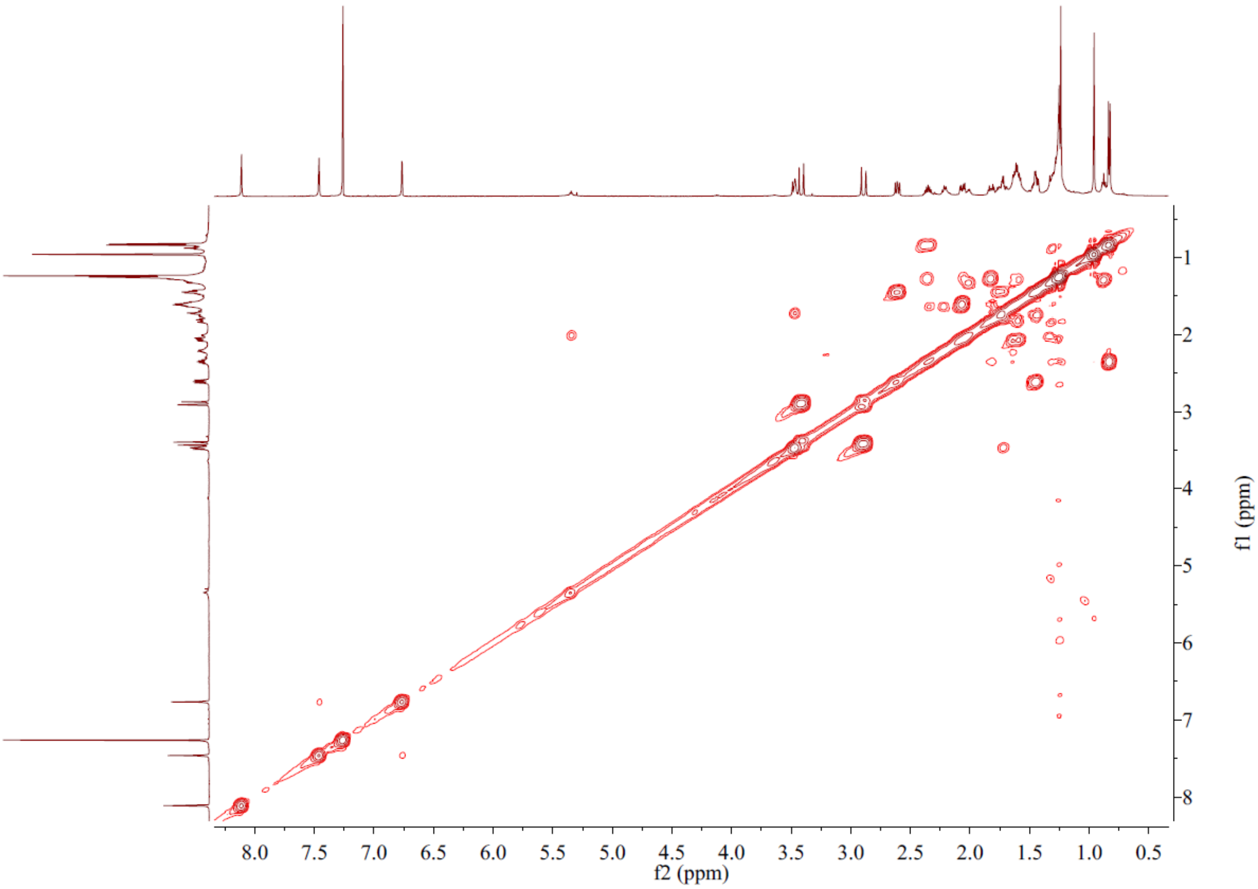

S3.5. HMBC spectrum of compound **1**.

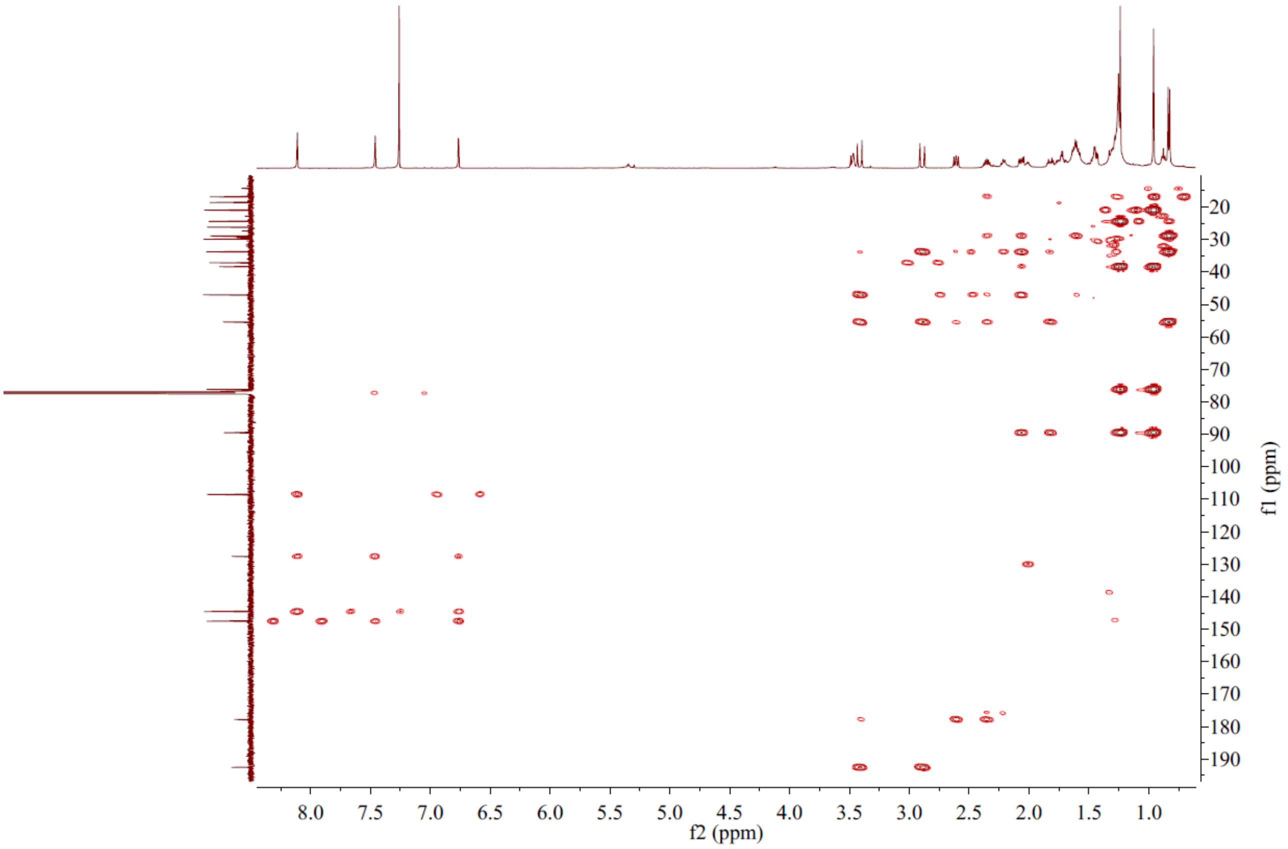

S3.6. NOESY spectrum of compound **1**.

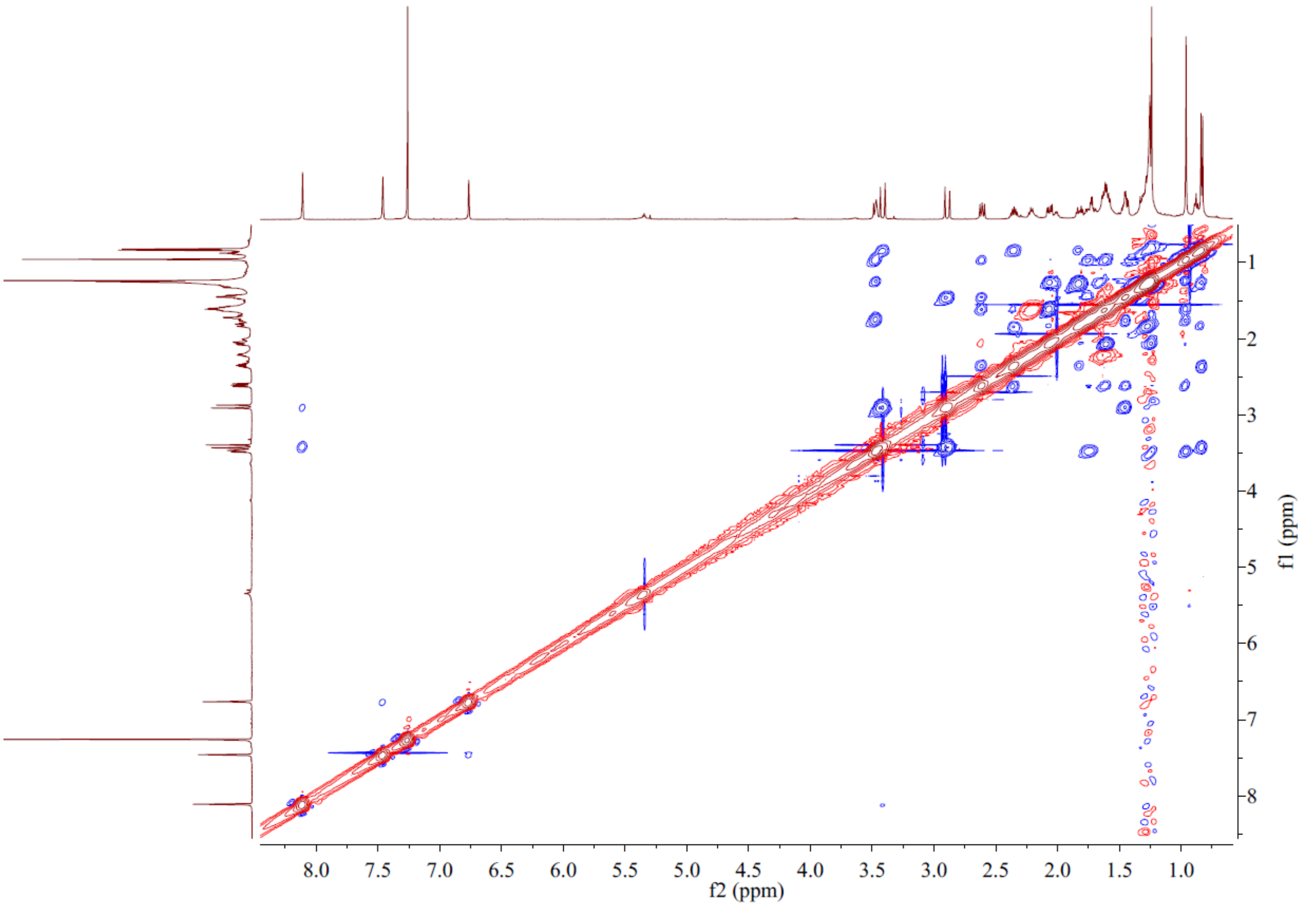

S3.7. ^{1}H NMR spectrum of compound **2**.

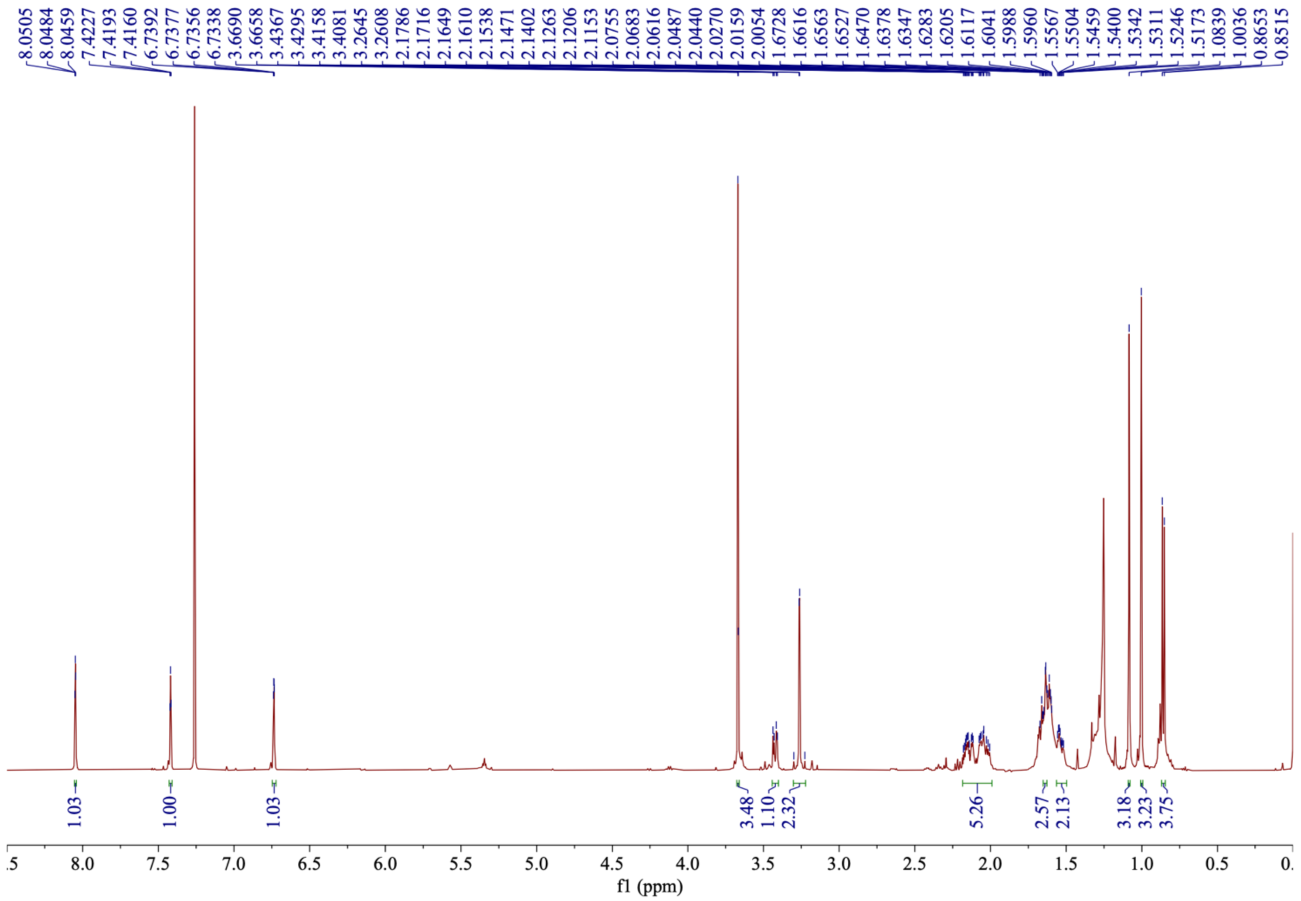

S3.8. ^{13}C NMR and DEPT spectra of compound **2**.

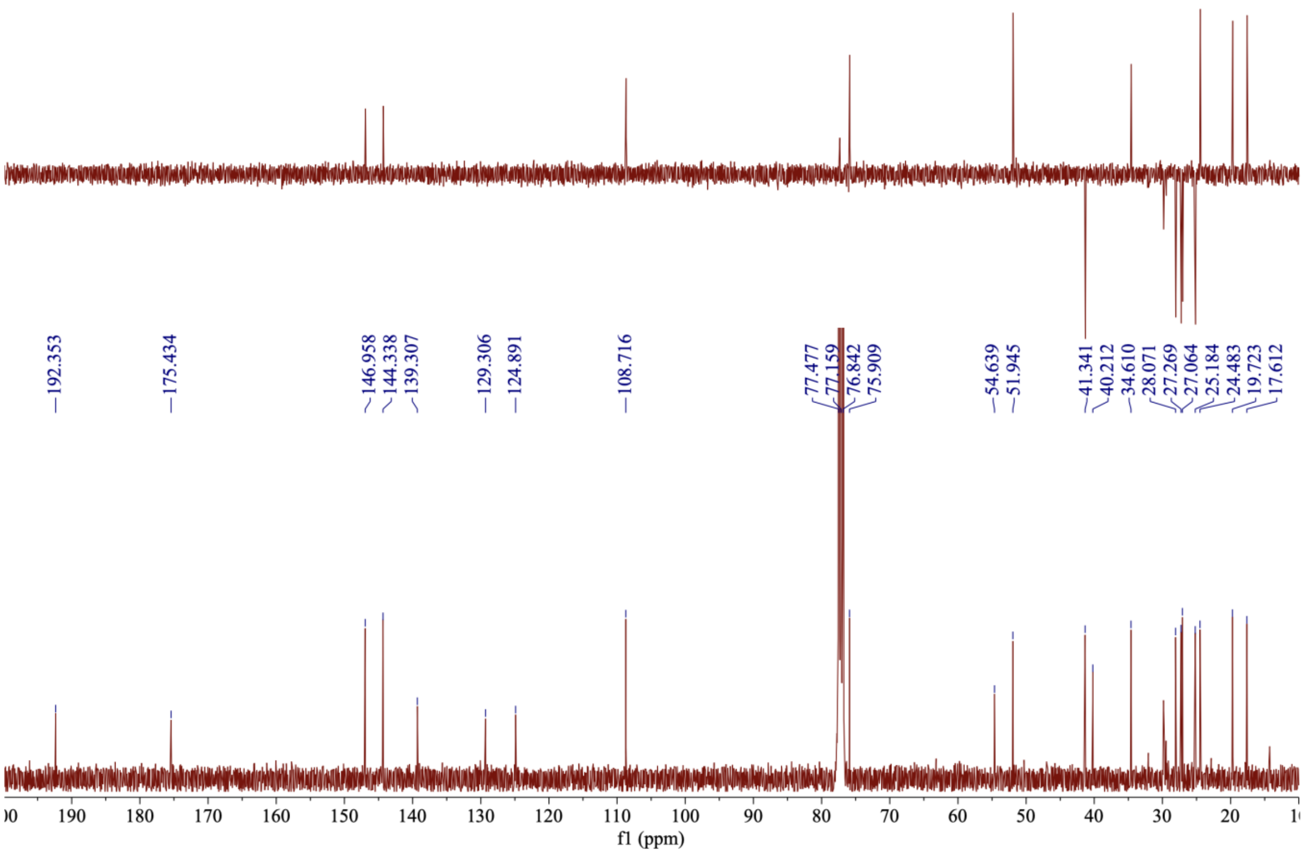

S3.9. HSQC spectrum of compound **2**.

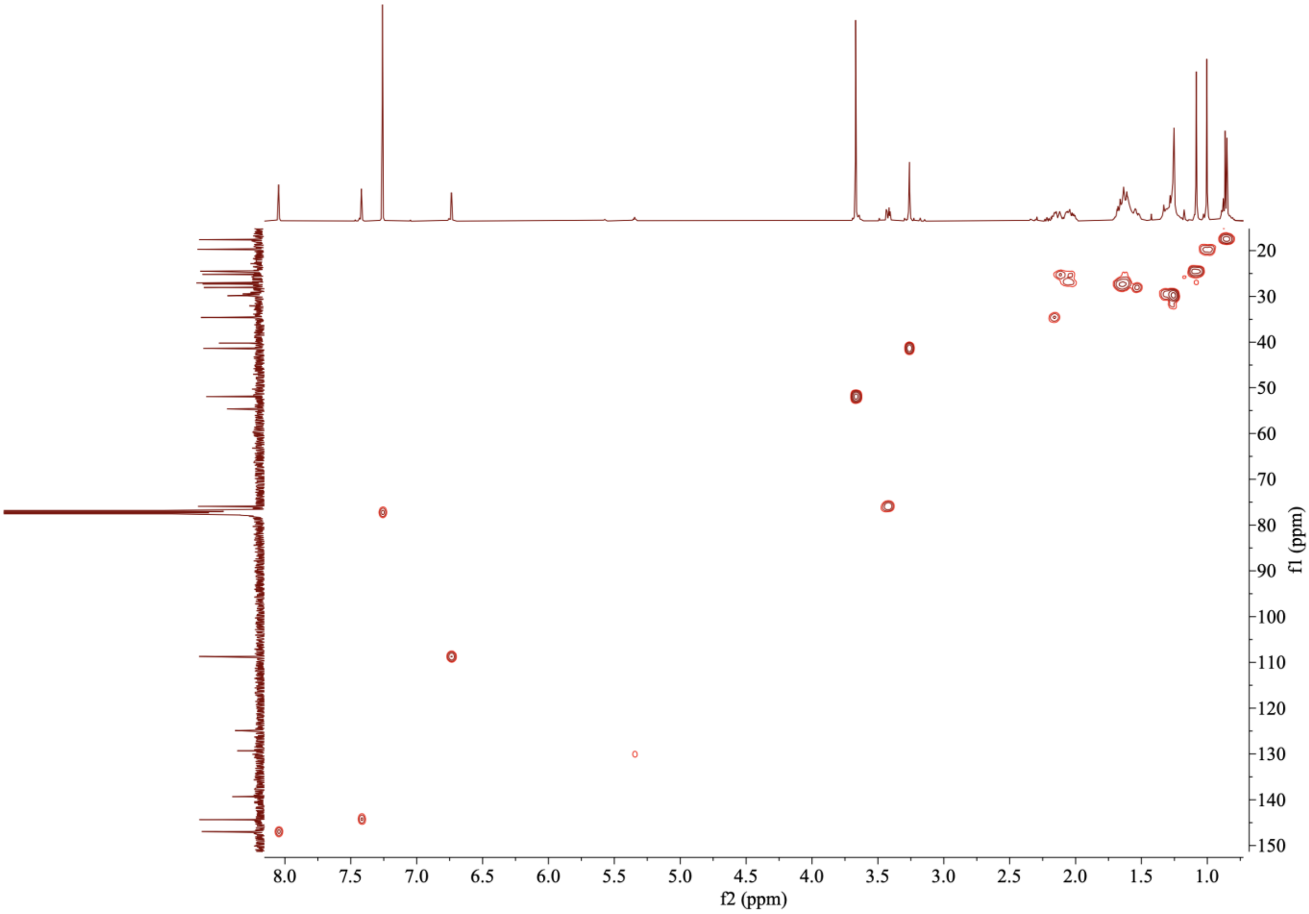

S3.10. ^{1}H–^{1}H COSY spectrum of compound **2**.

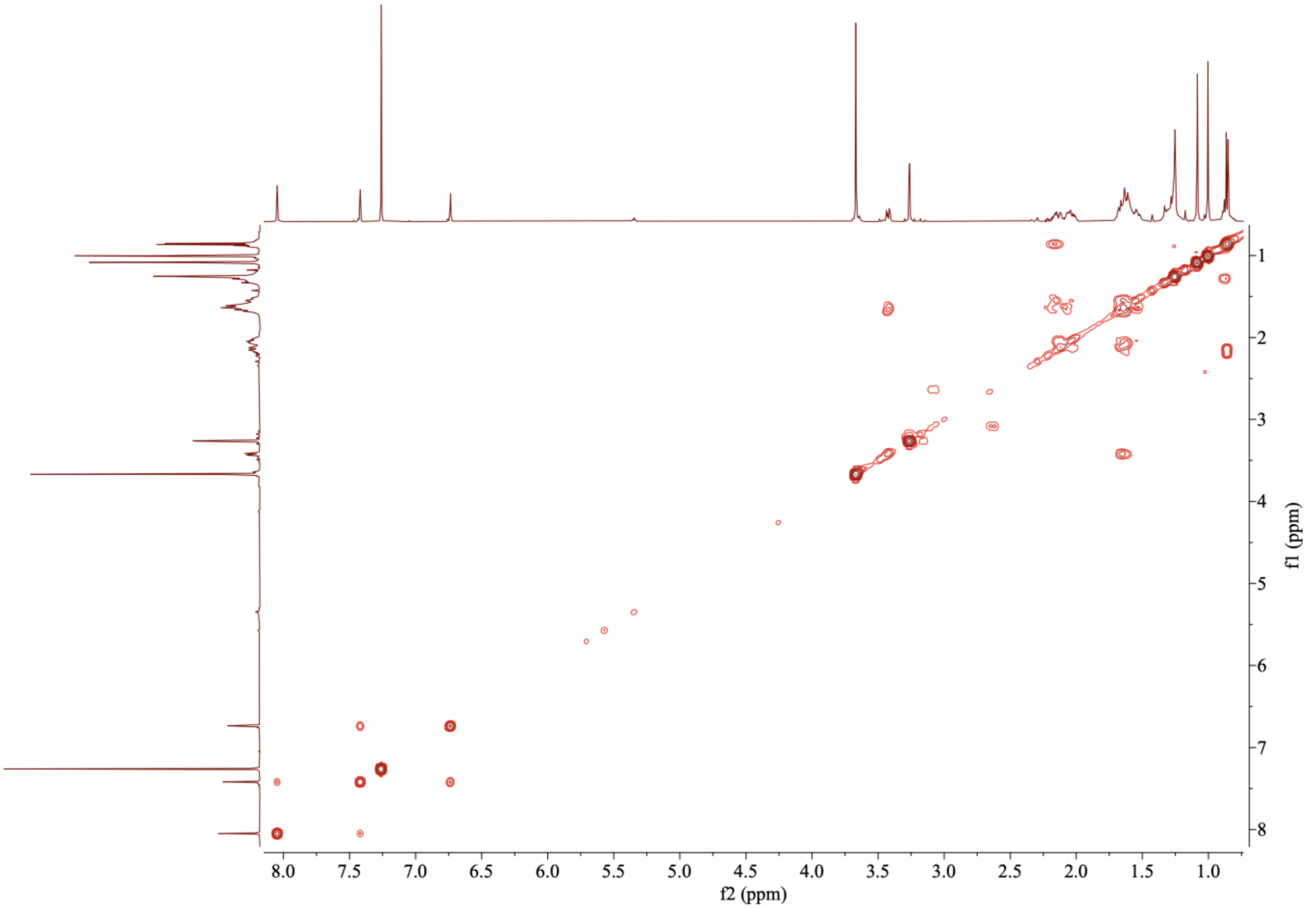

S3.11. HMBC spectrum of compound **2**.

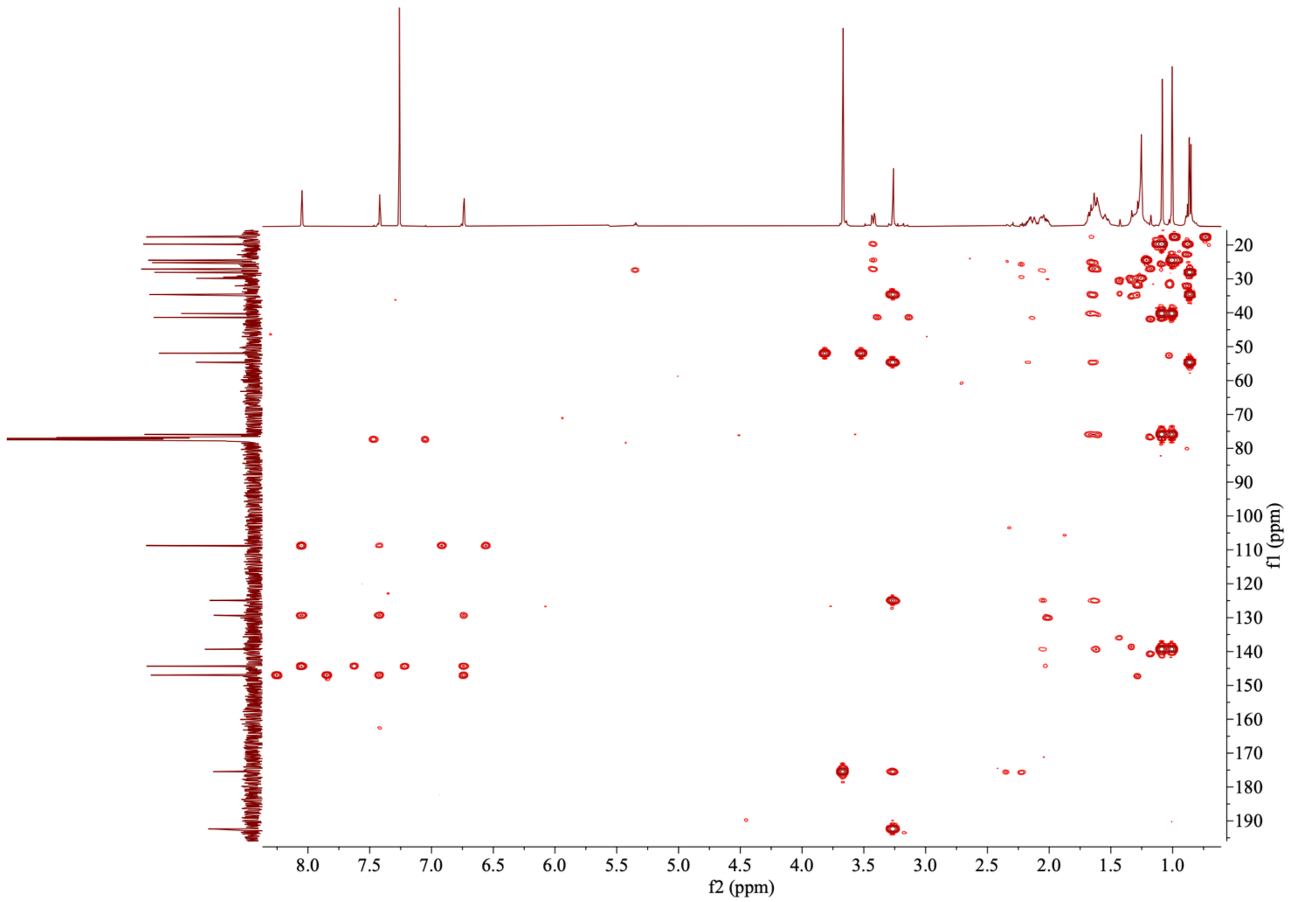

S3.12. NOESY spectrum of compound **2**.

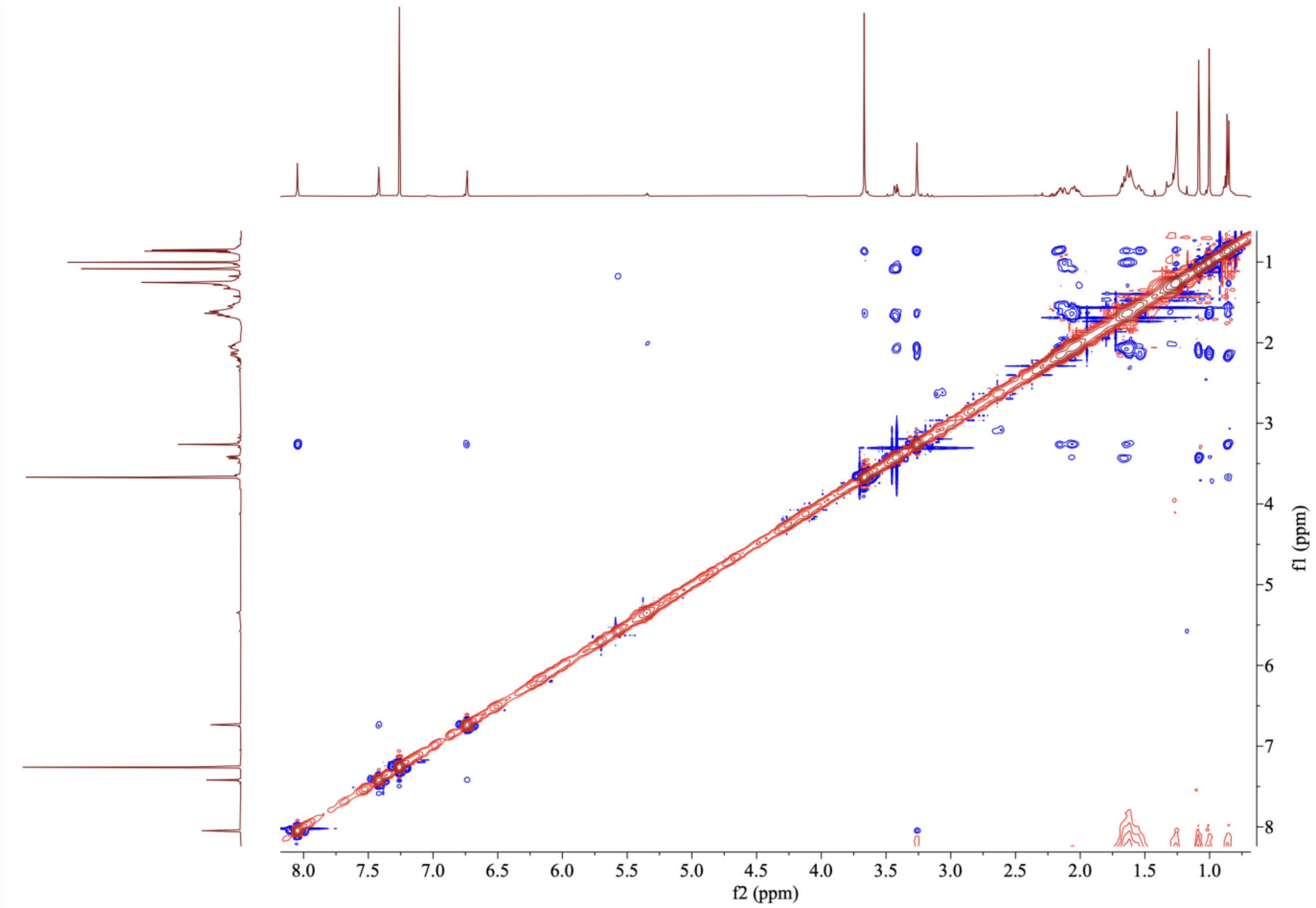

S3.13. ^{1}H NMR spectrum of compound **3**.

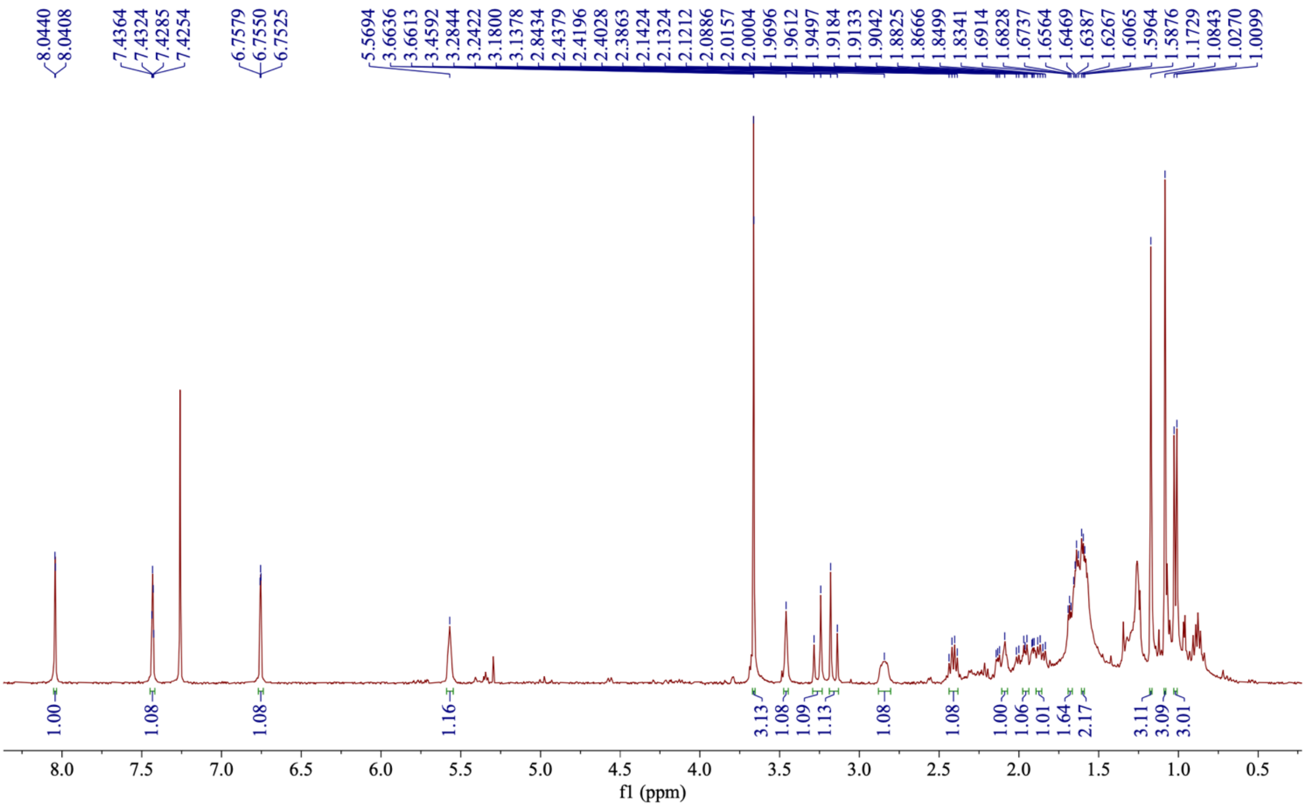

S3.14. ^{13}C NMR spectrum of compound **3**.

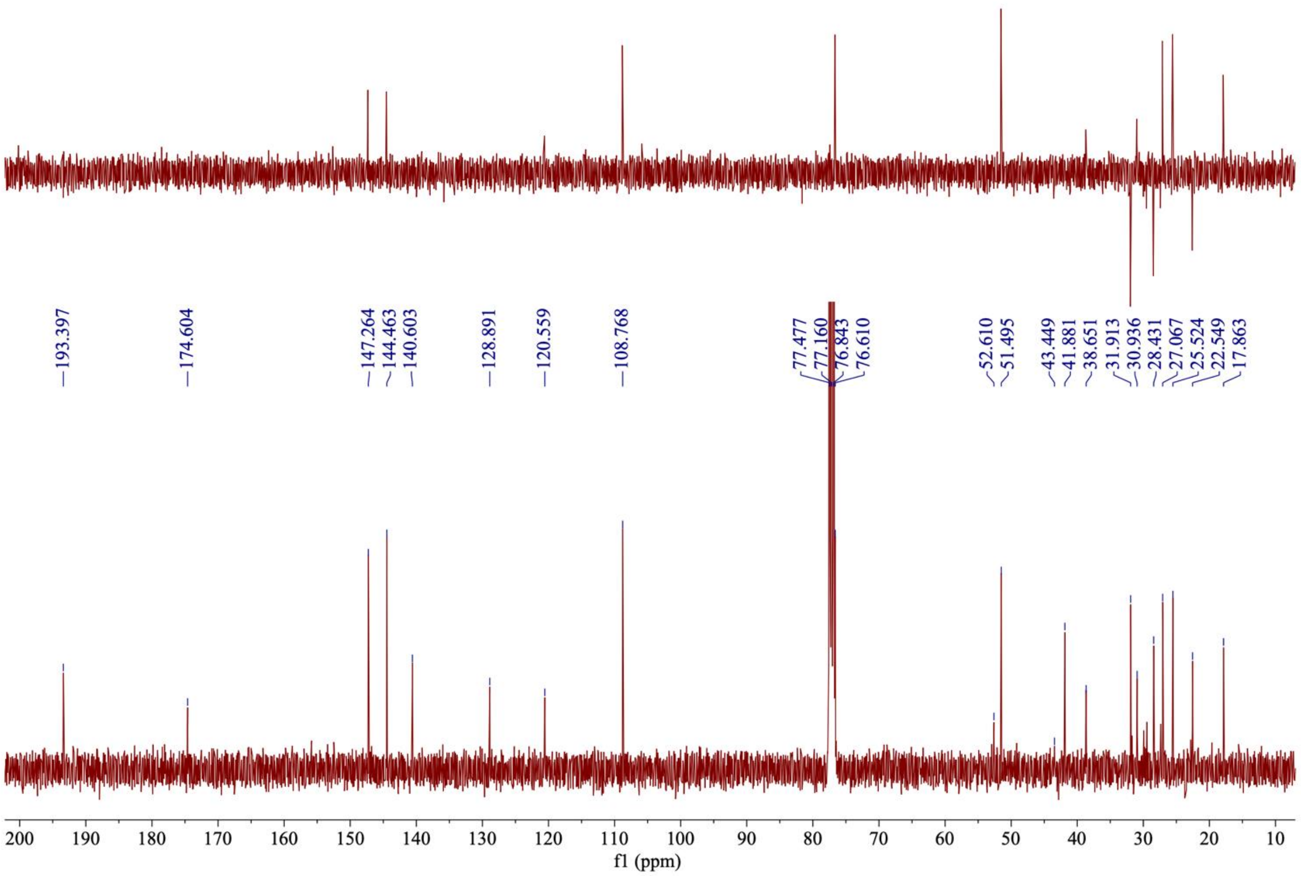

S3.15. HSQC spectrum of compound **3**.

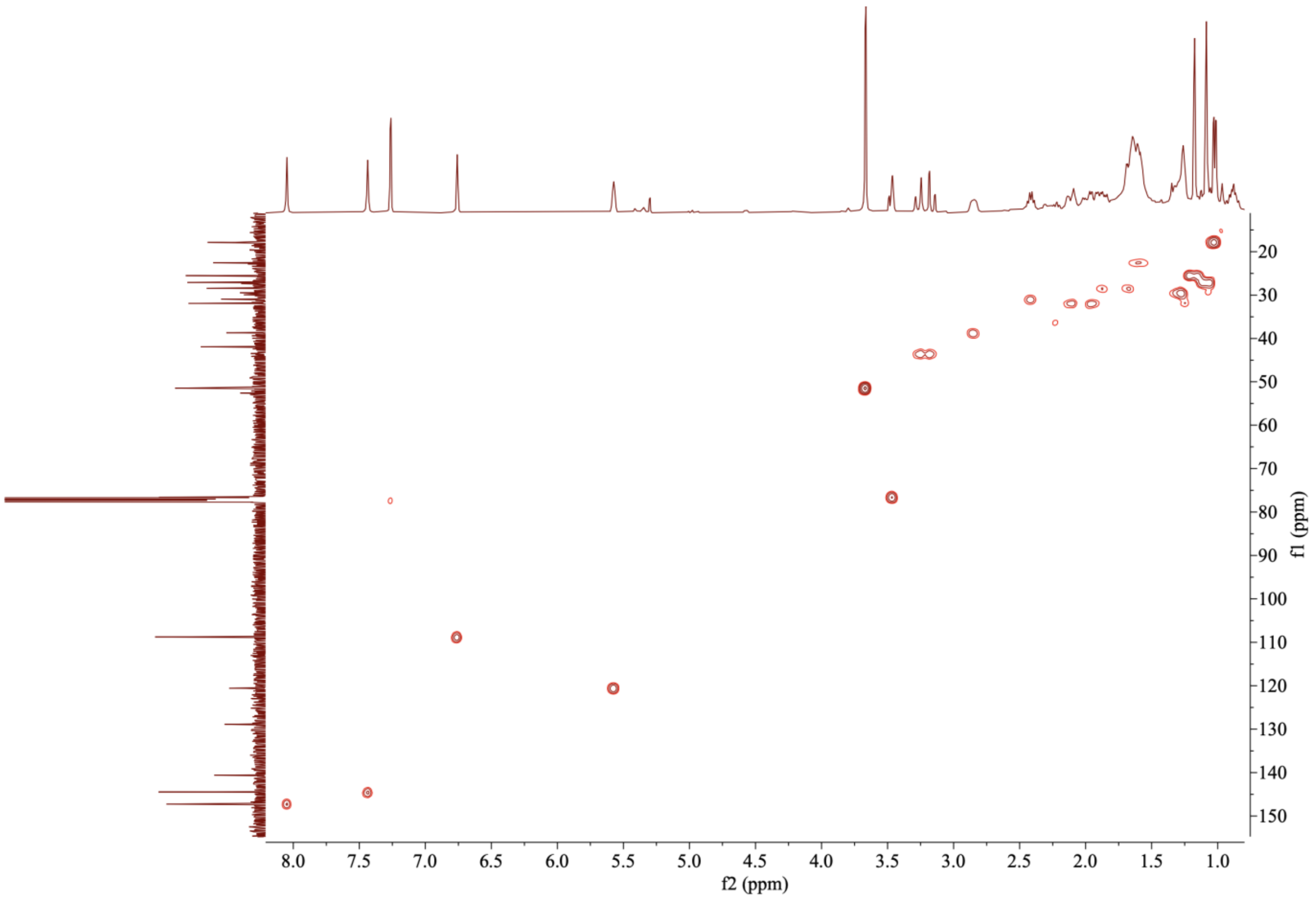

S3.16. ^{1}H–^{1}H COSY spectrum of compound **3**.

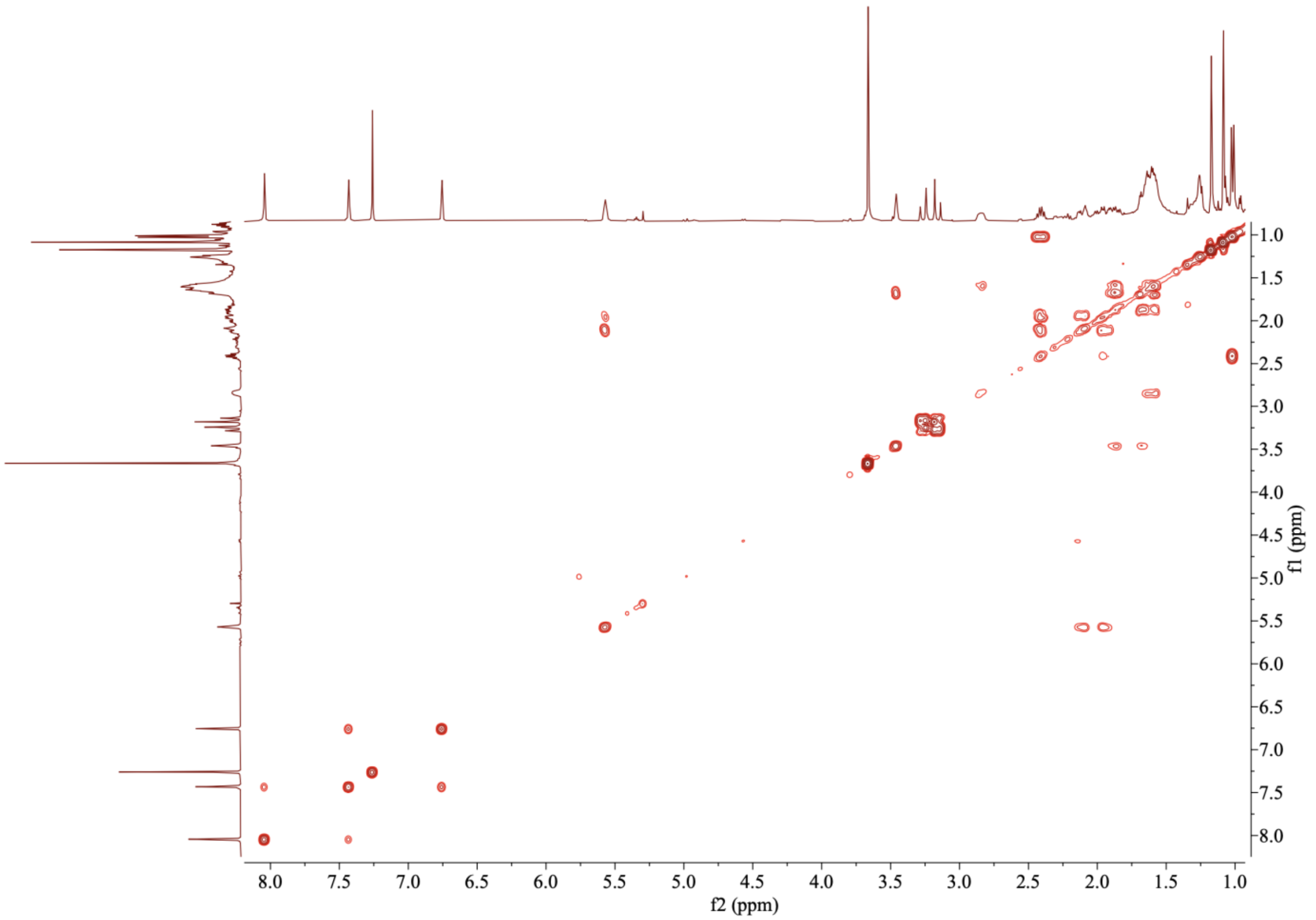

S3.17. HMBC spectrum of compound **3**.

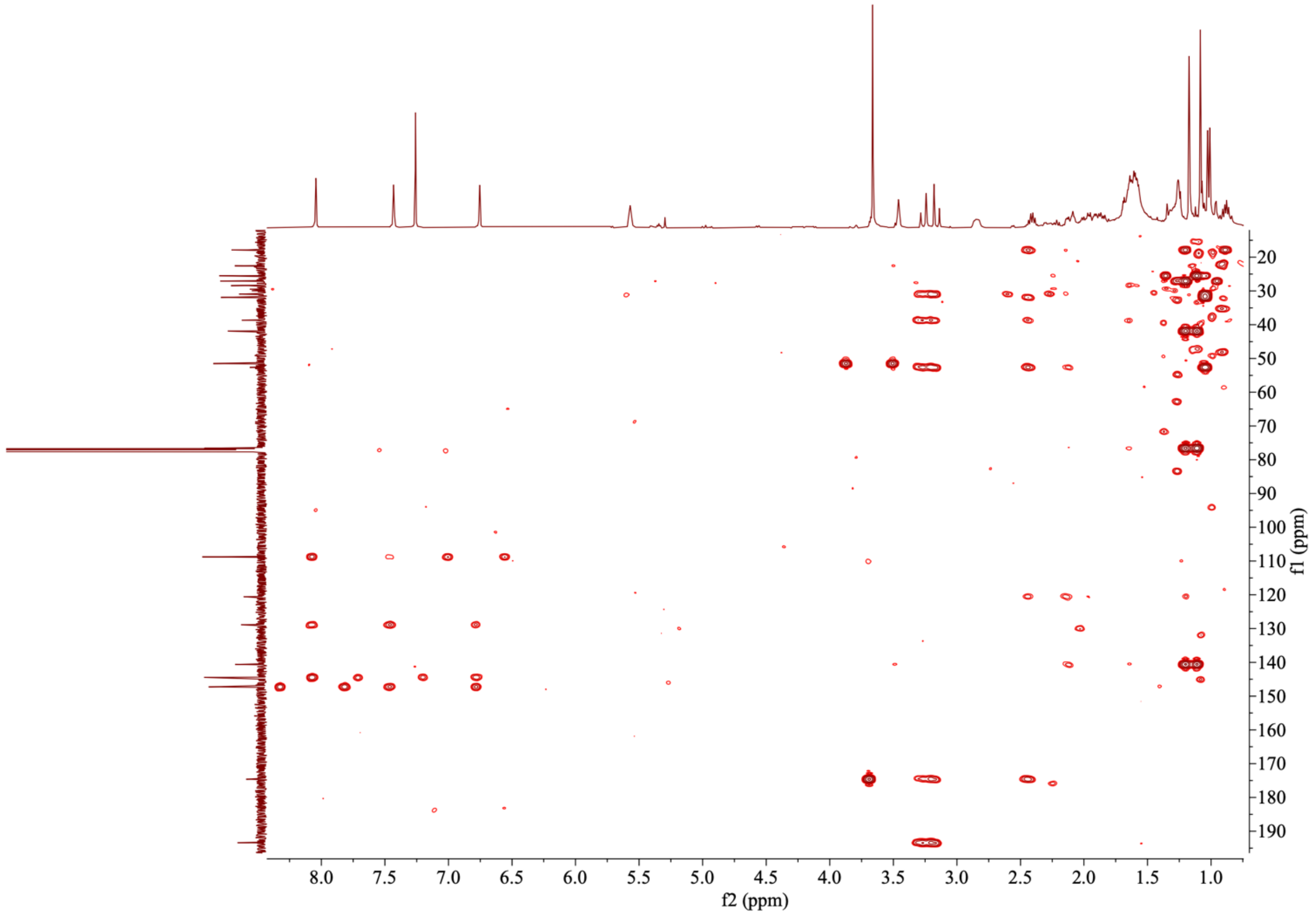

S3.18. NOESY spectrum of compound **3**.

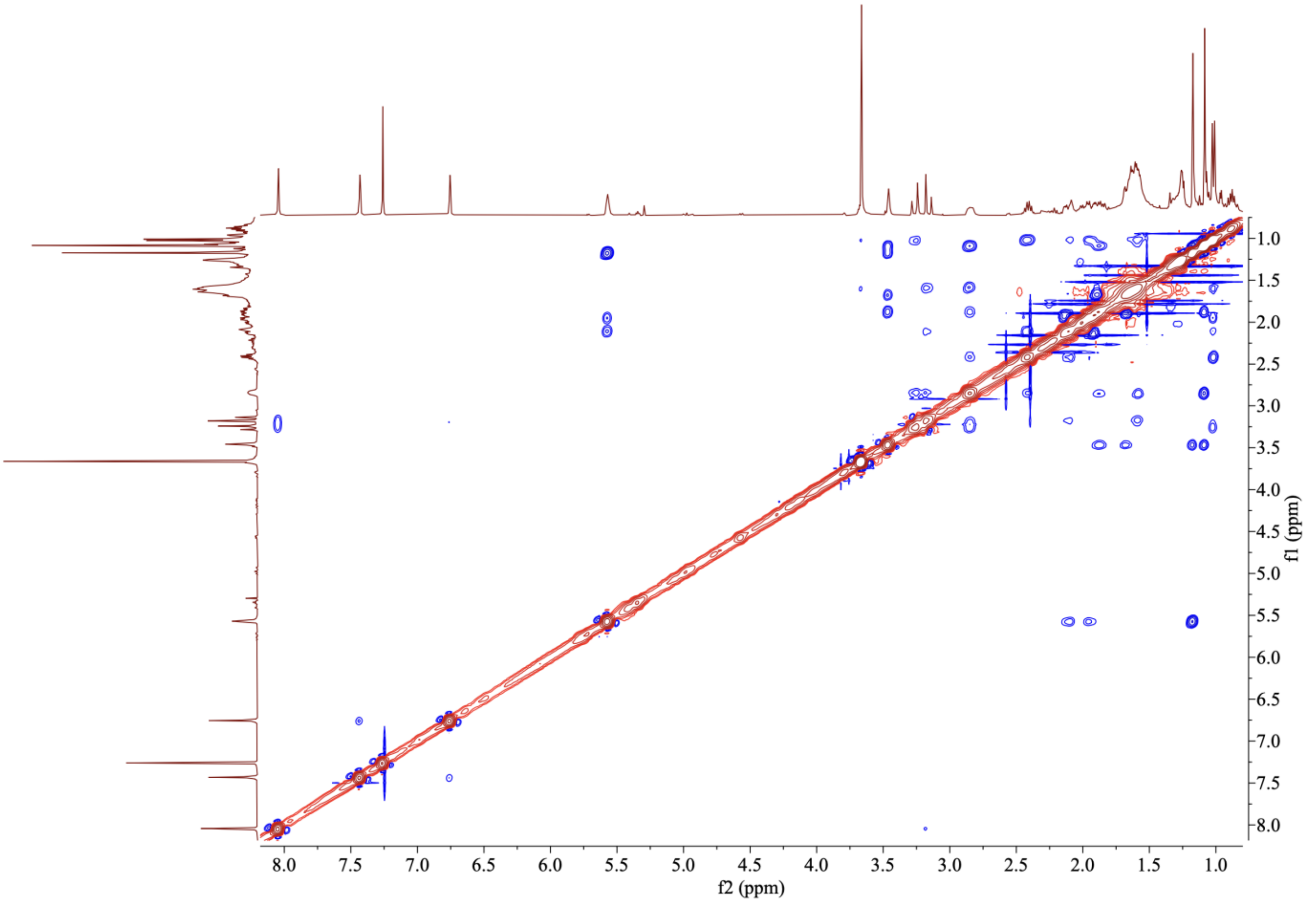

S3.19. ^{1}H NMR spectrum of compound **4**.

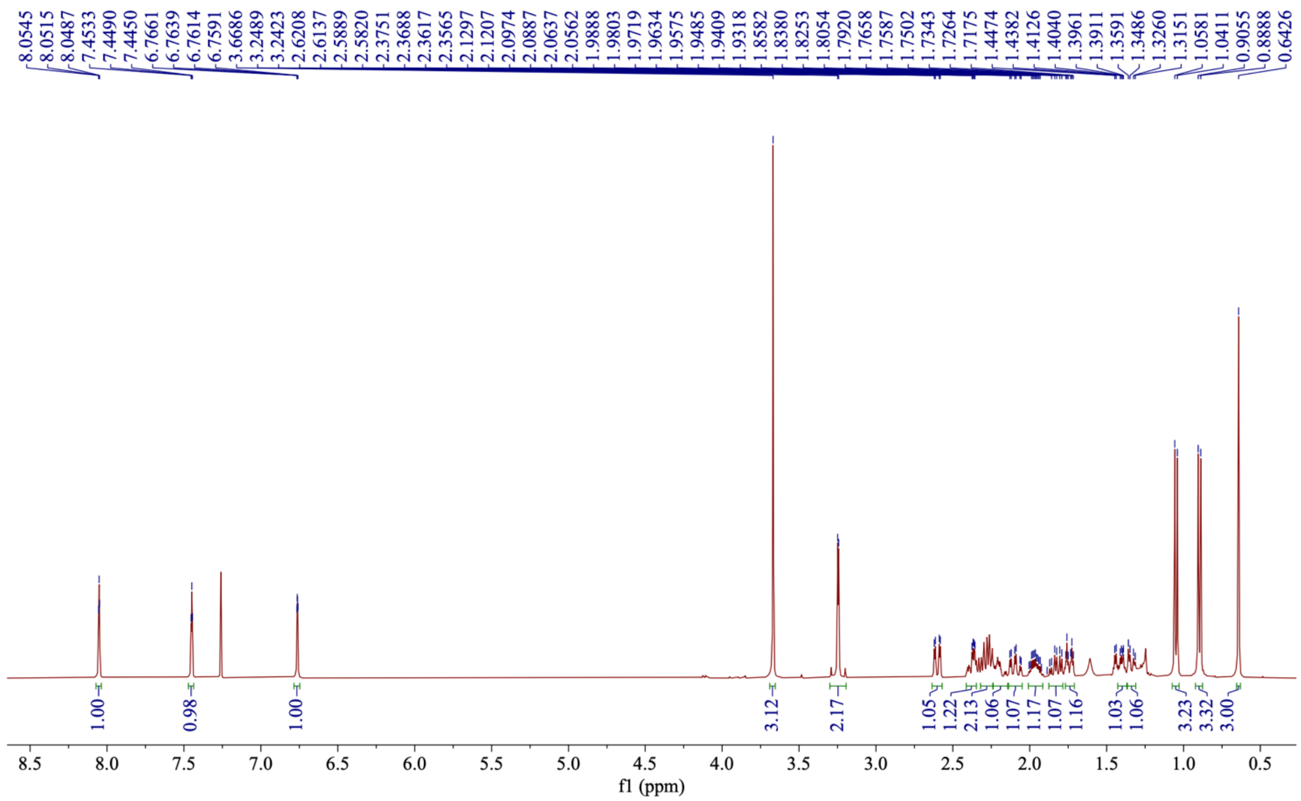

S3.20. ^{13}C NMR and DEPT spectra of compound **4**.

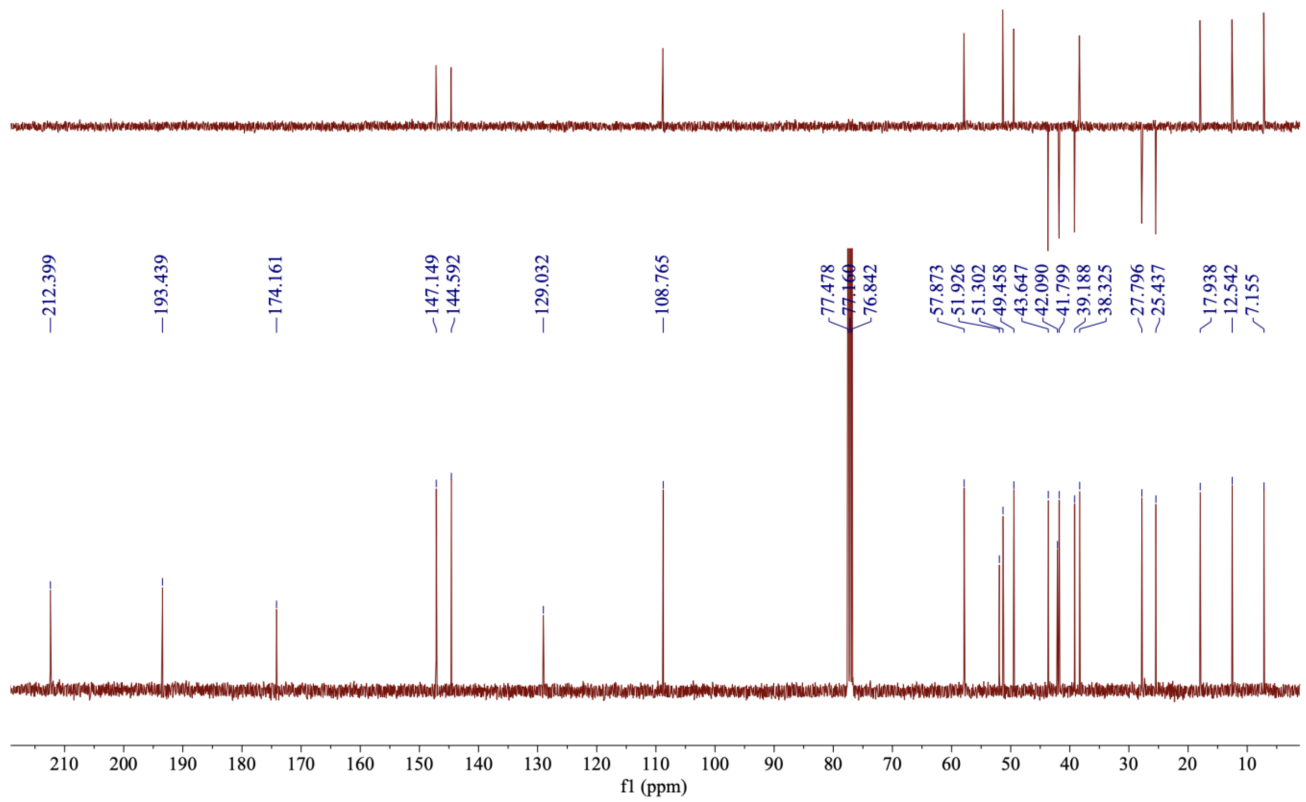

S3.21. HSQC spectrum of compound **4**.

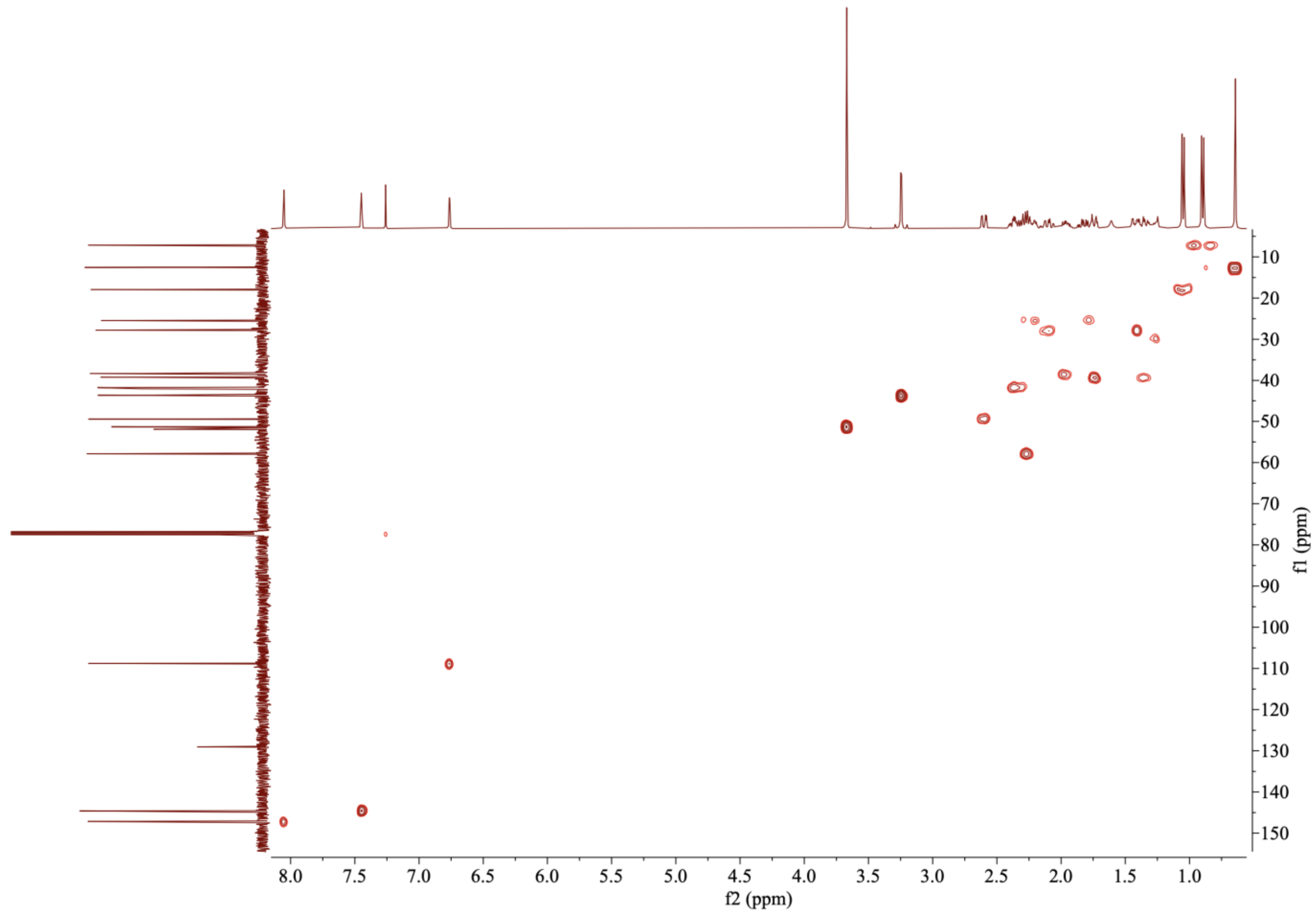

S3.22. ^{1}H–^{1}H COSY spectrum of compound **4**.

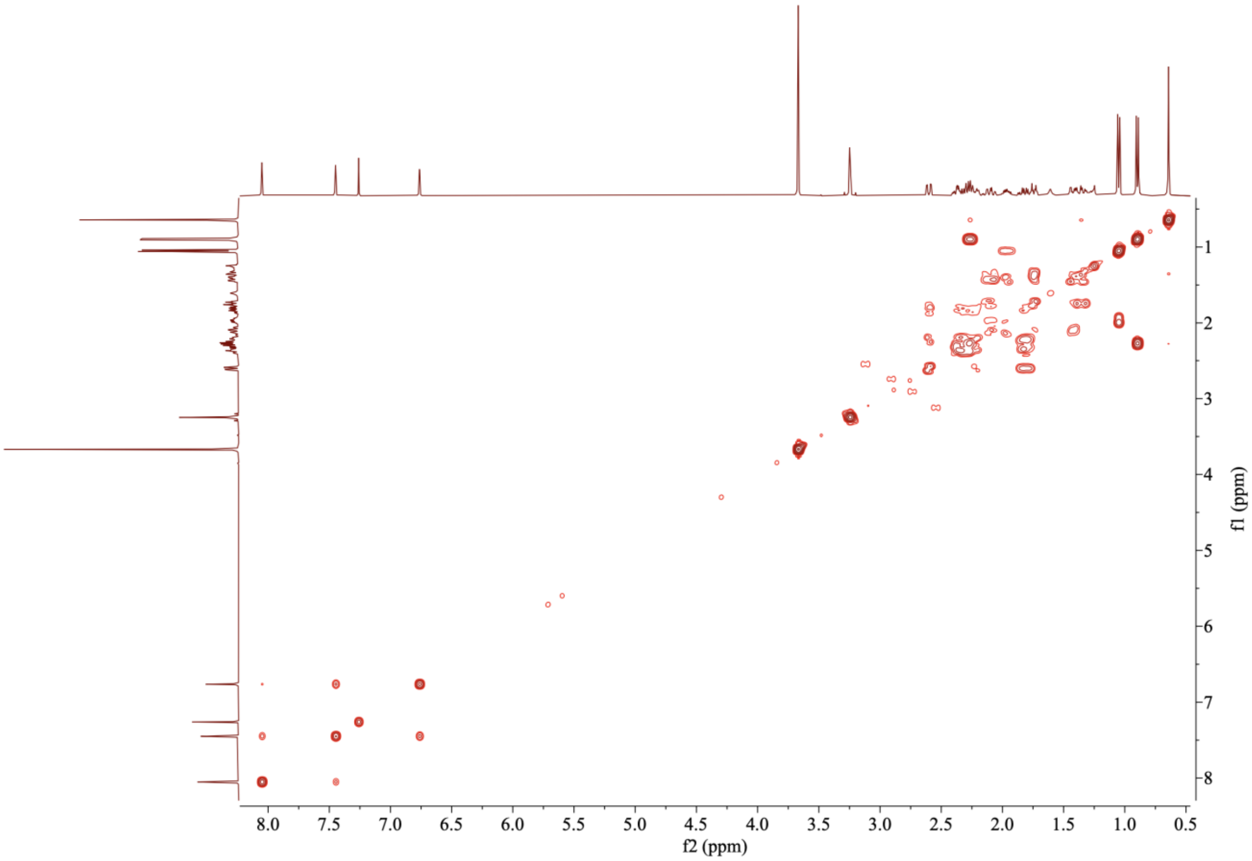

S3.23. HMBC spectrum of compound **4**.

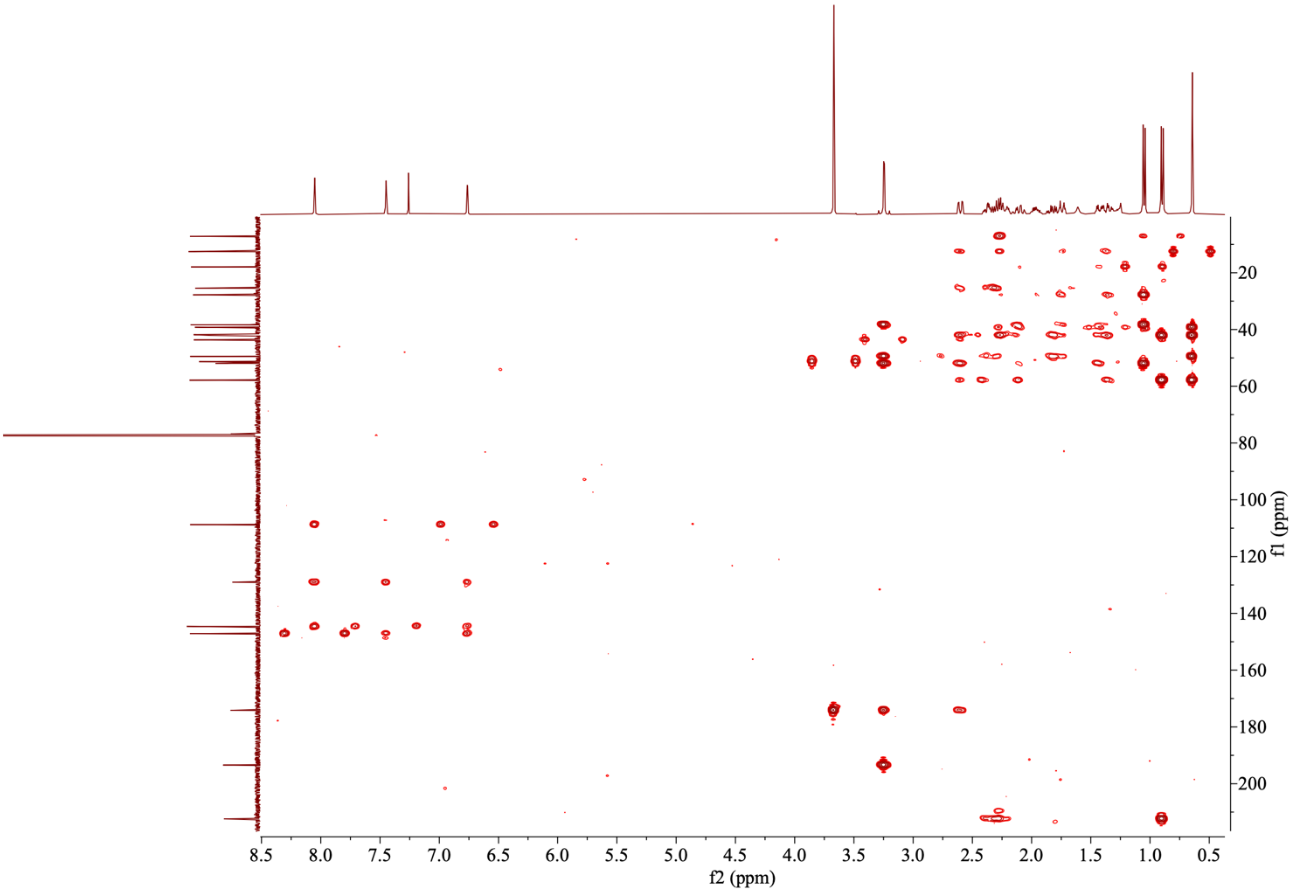

S3.24. NOESY spectrum of compound **4**.

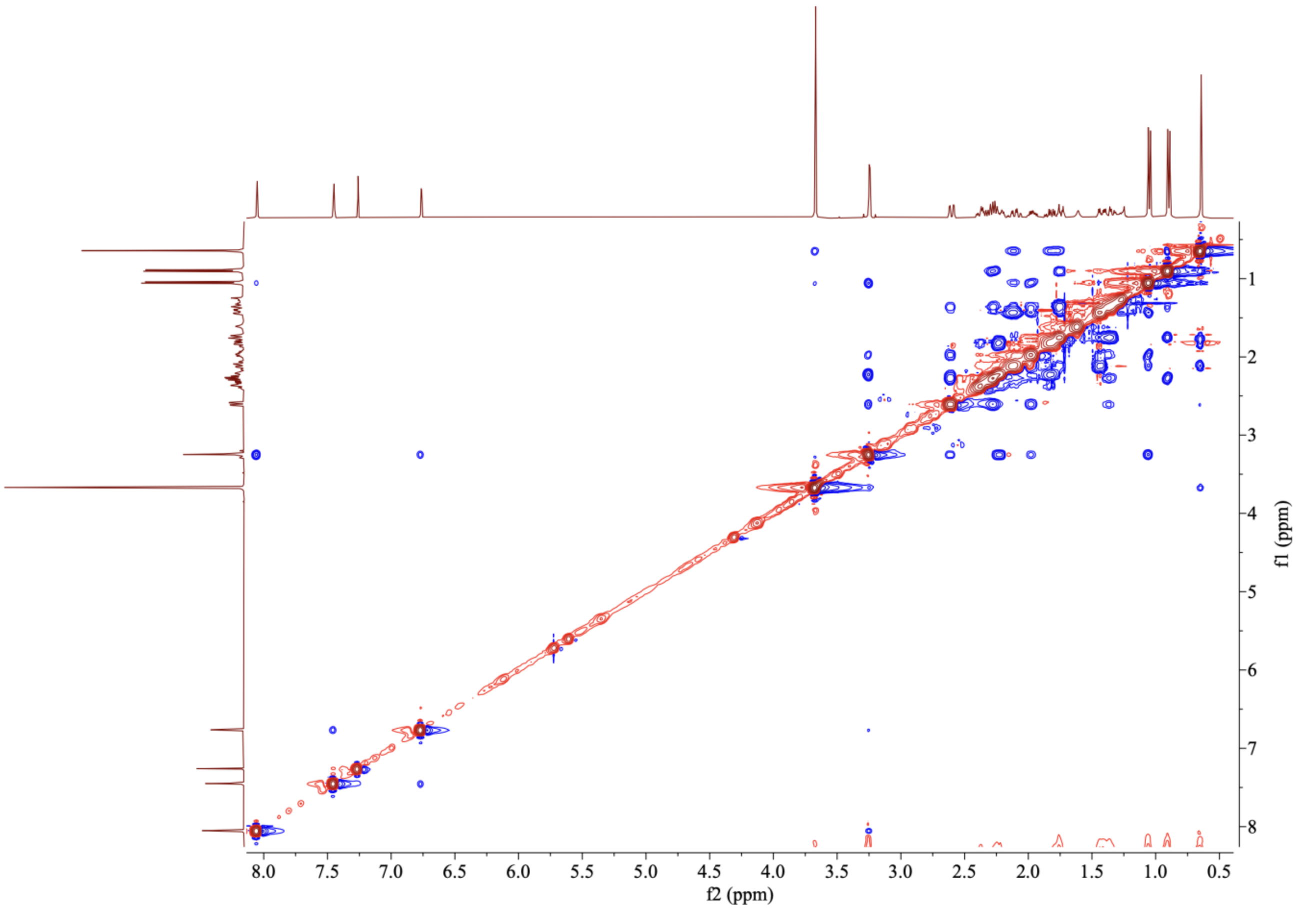

S3.25. ^{1}H NMR spectrum of compound **5**.

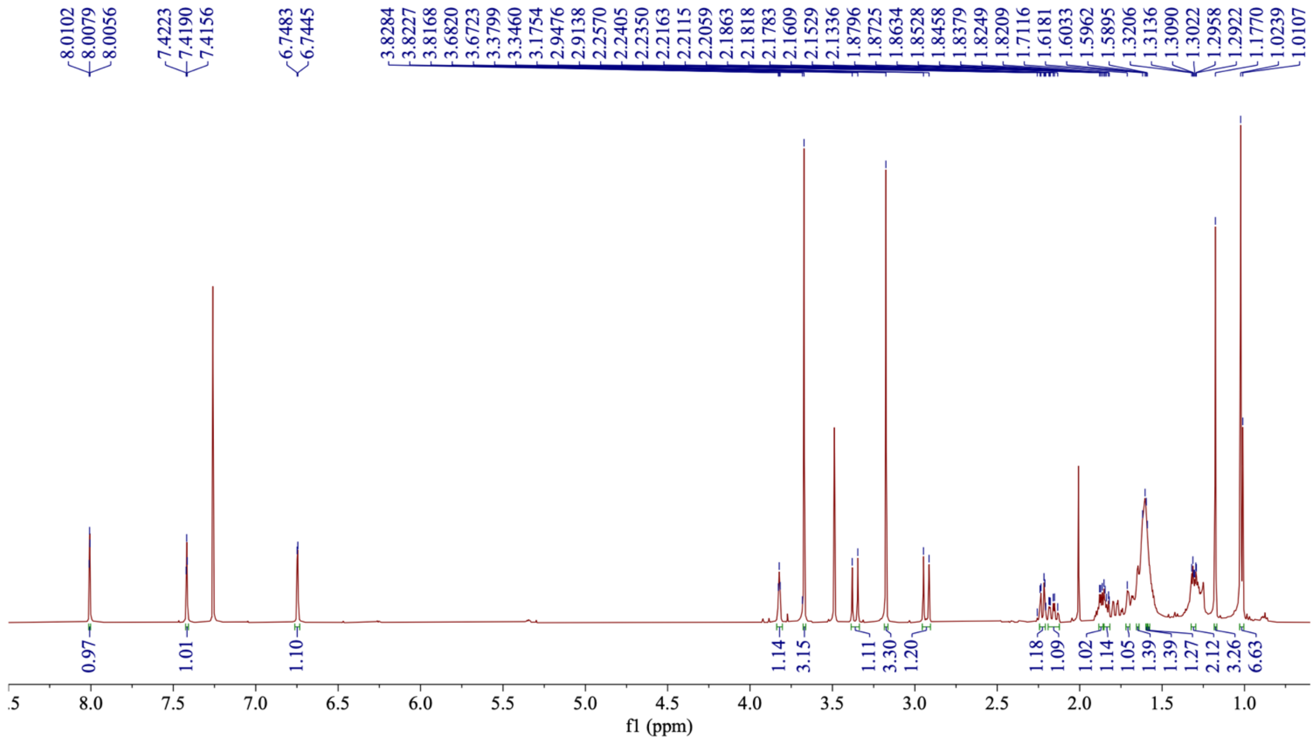

S3.26. ^{13}C NMR and DEPT spectra of compound **5**.

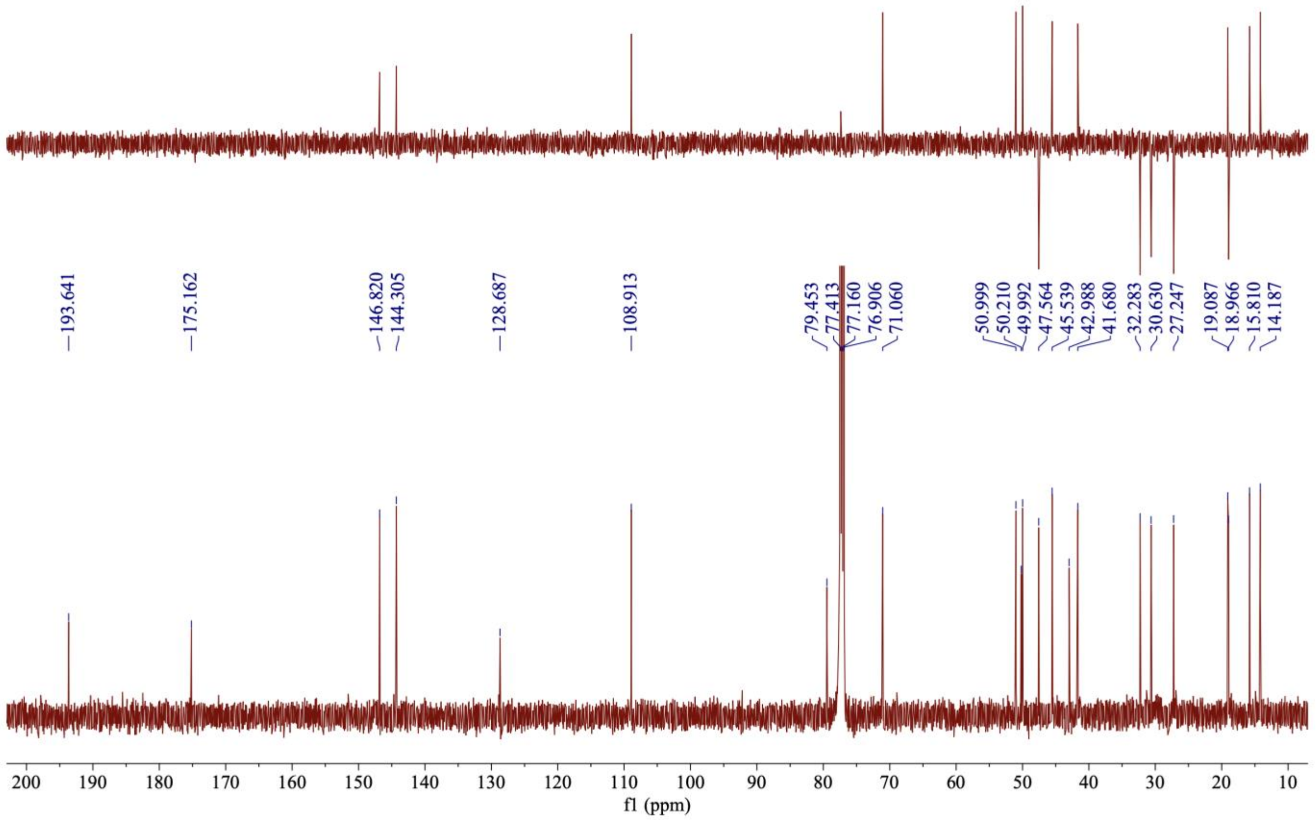

S3.27. HSQC spectrum of compound **5**.

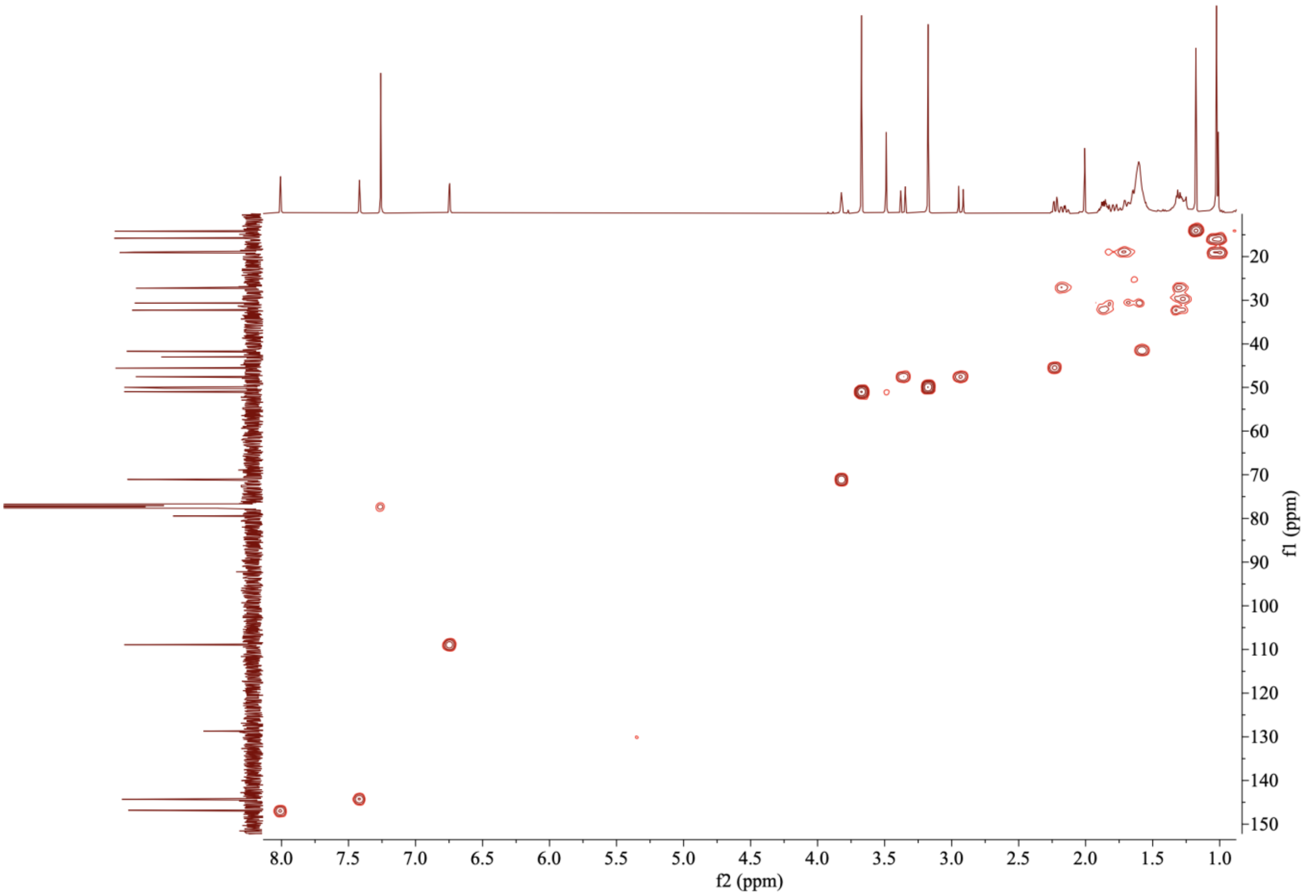

S3.28. ^{1}H–^{1}H COSY spectrum of compound **5**.

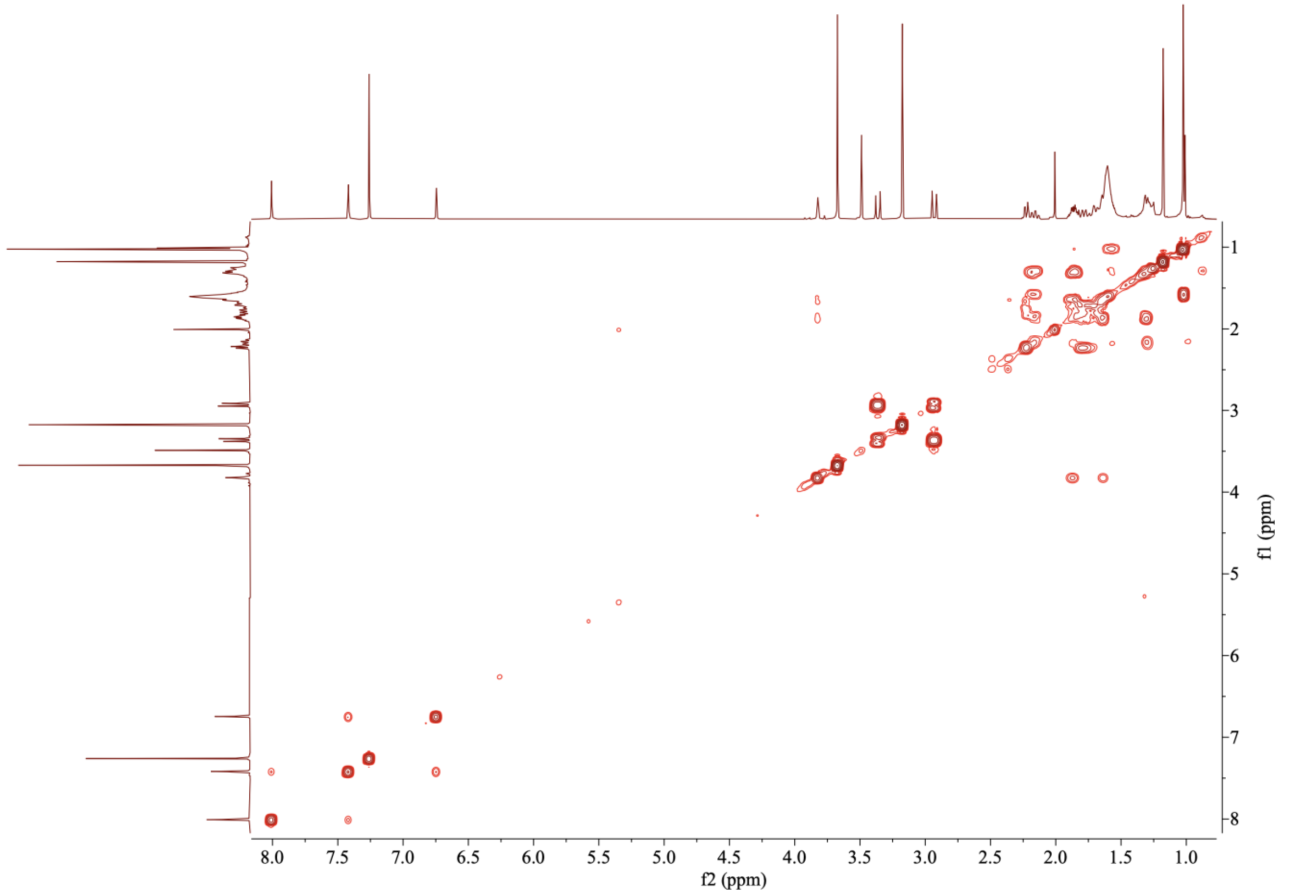

S3.29. HMBC spectrum of compound **5**.

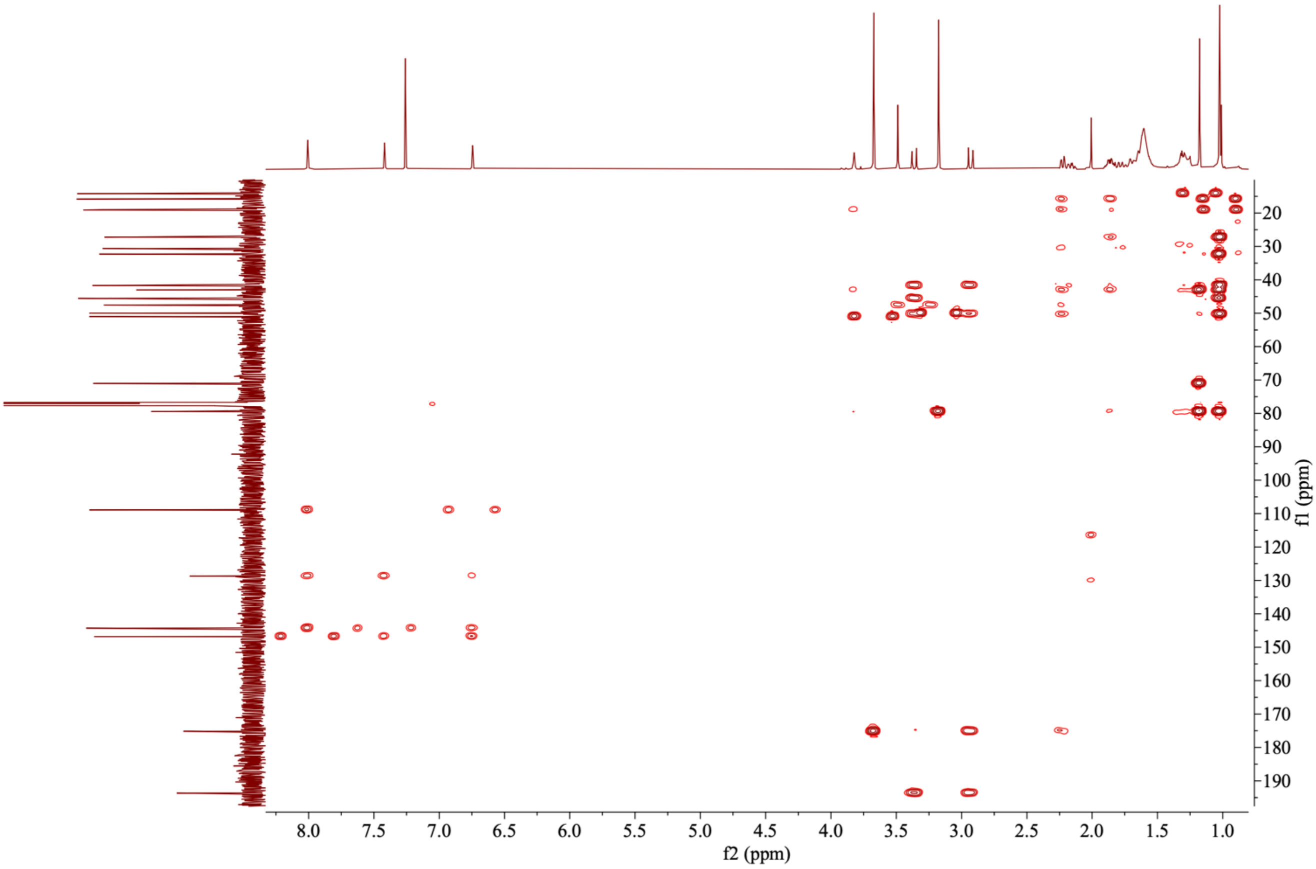

S3.30. NOESY spectrum of compound **5**.

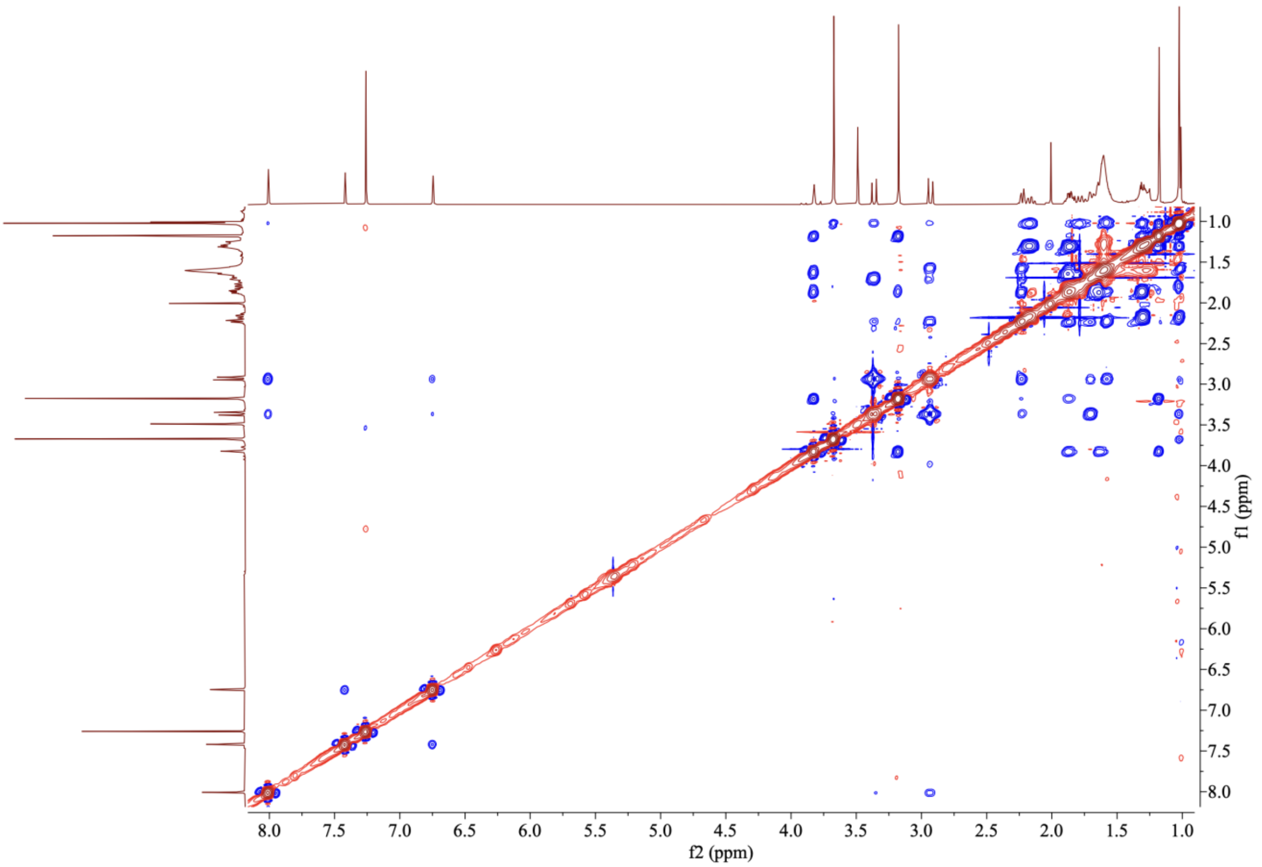

S3.31. ^{1}H NMR spectrum of compound **6**.

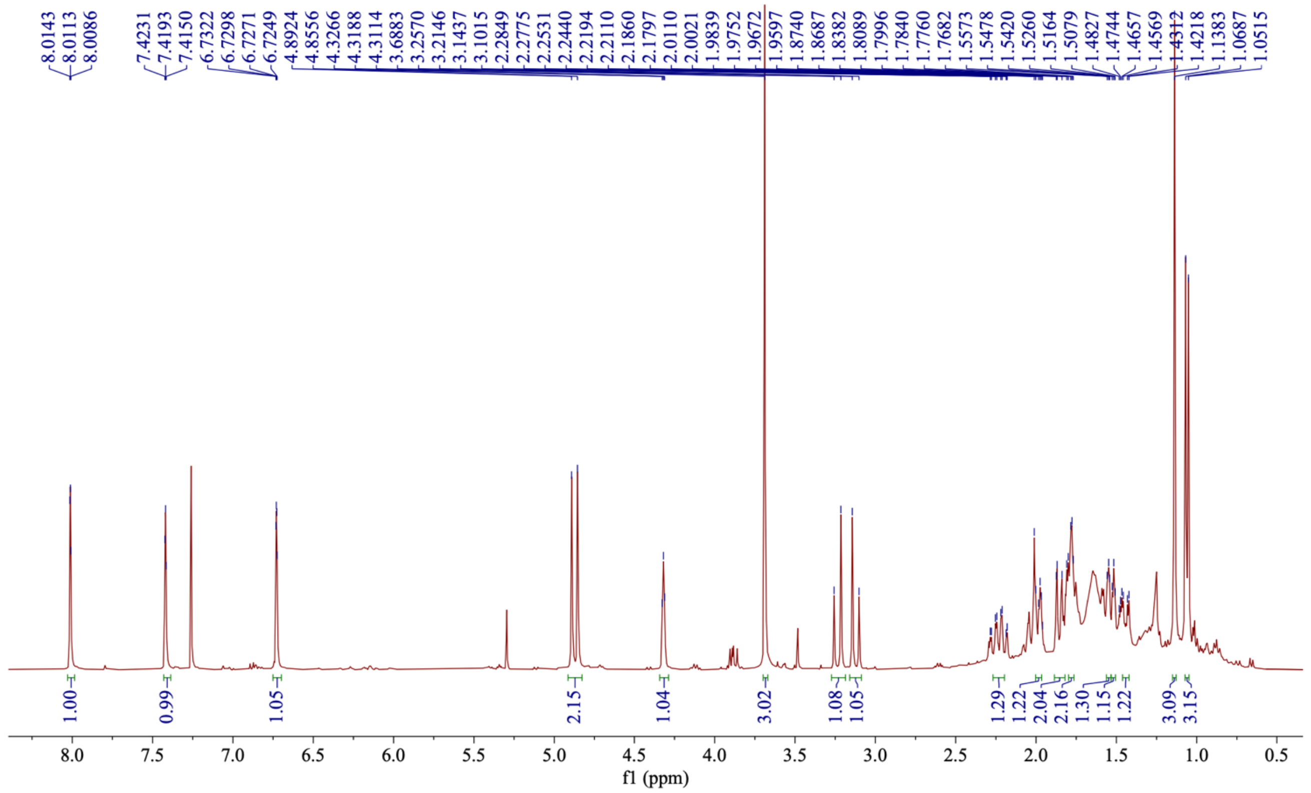

S3.32. ^{13}C NMR and DEPT spectra of compound **6**.

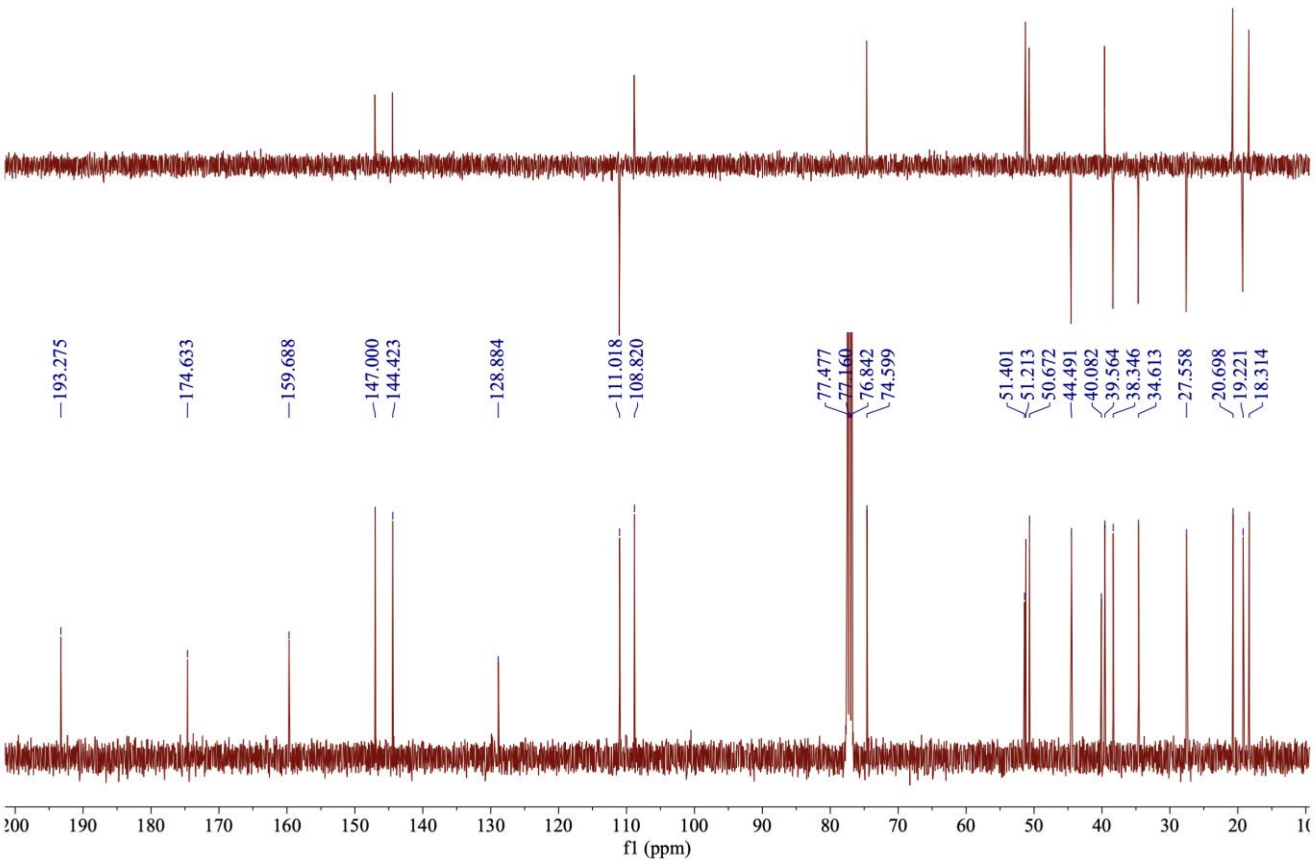

S3.33. HSQC spectrum of compound **6**.

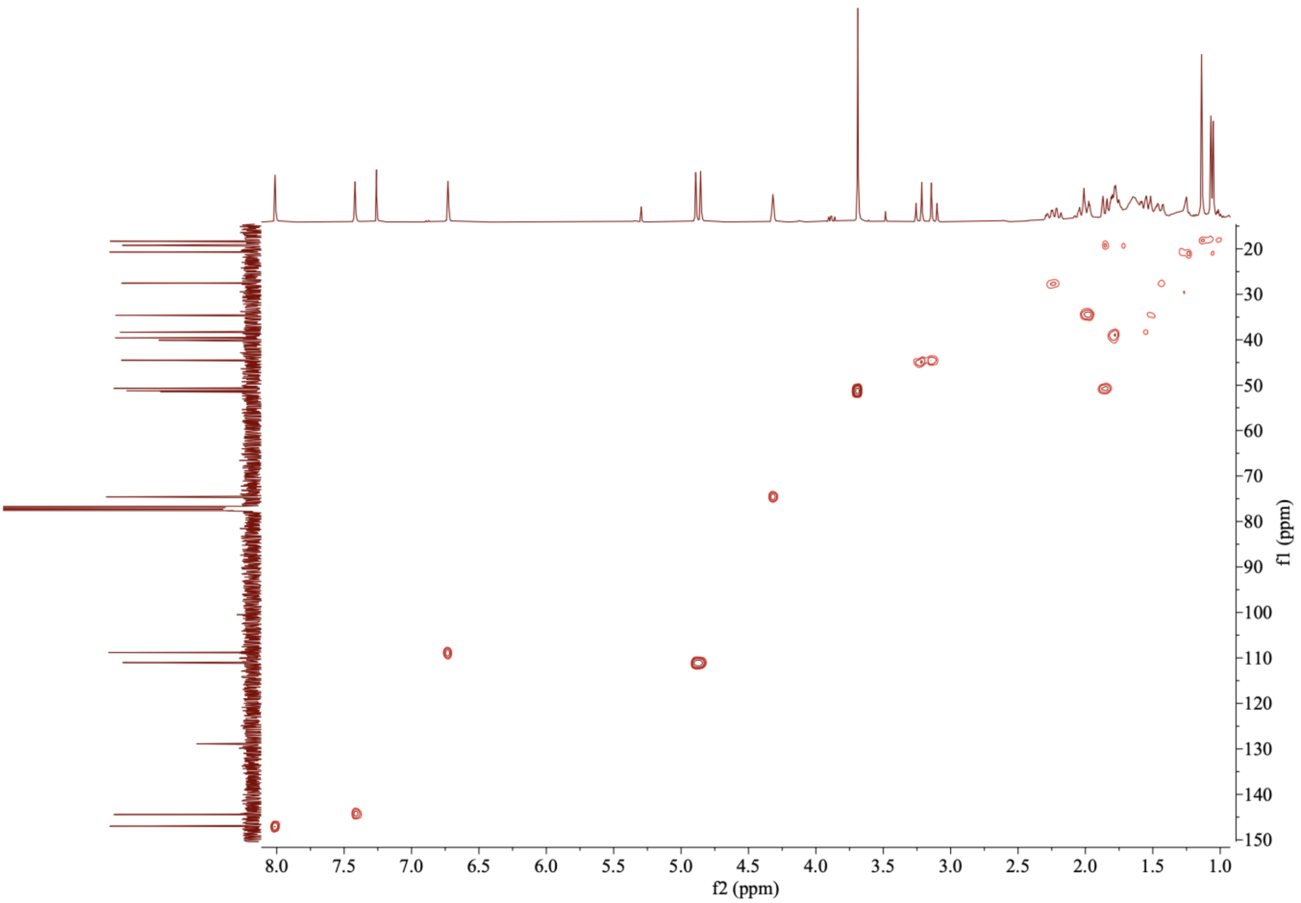

S3.34. ^{1}H–^{1}H COSY spectrum of compound **6**.

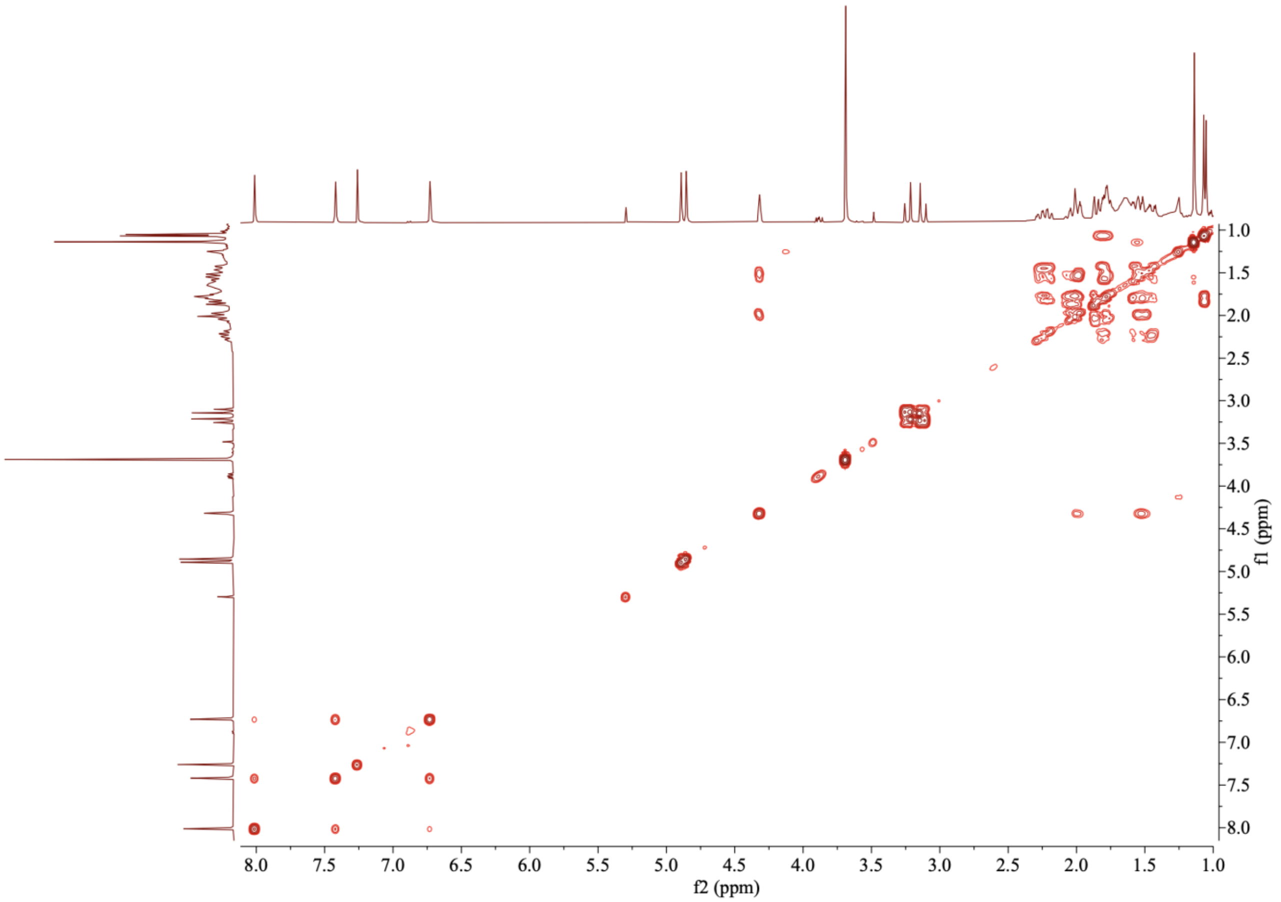

S3.35. HMBC spectrum of compound **6**.

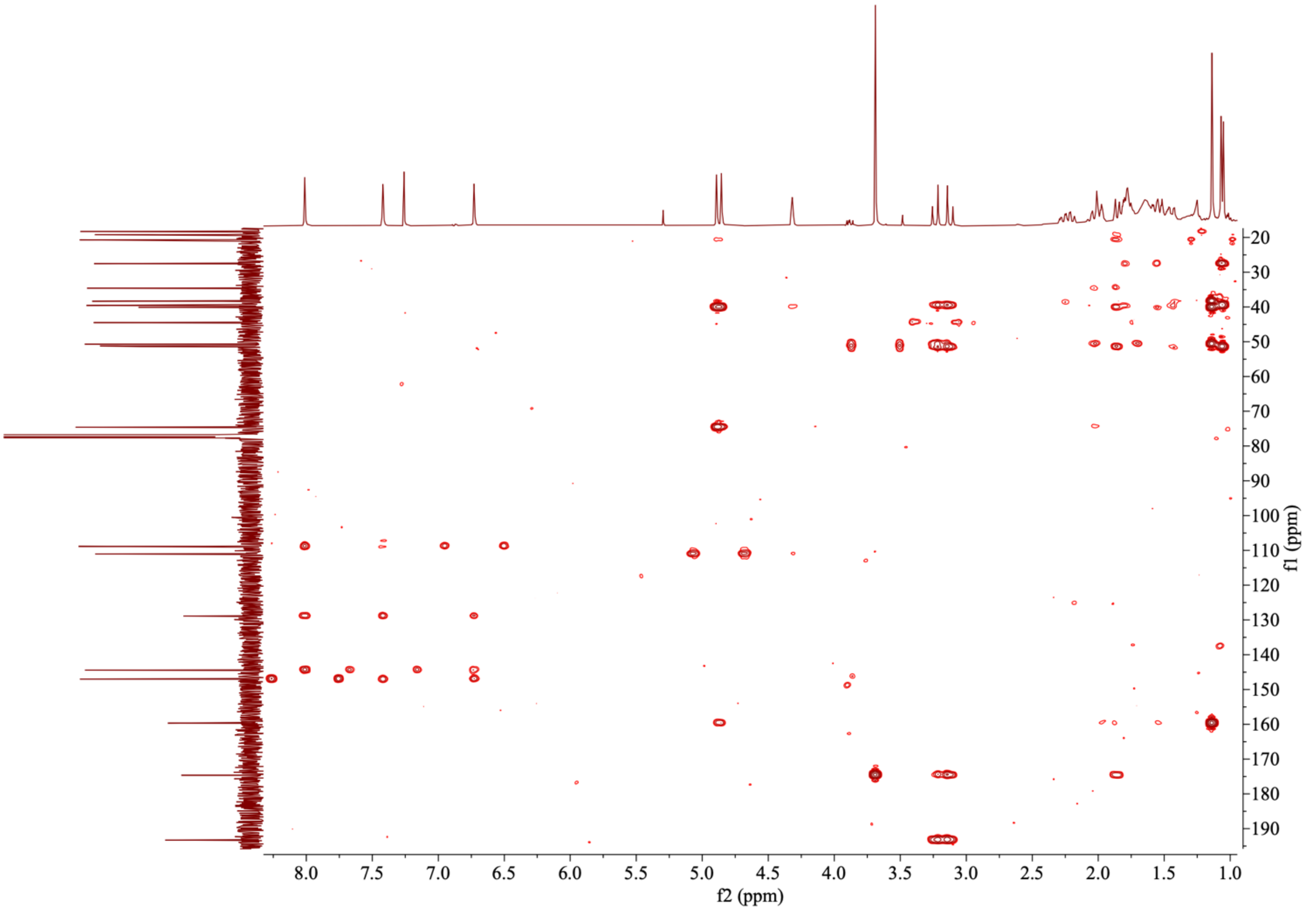

S3.36. NOESY spectrum of compound **6**.

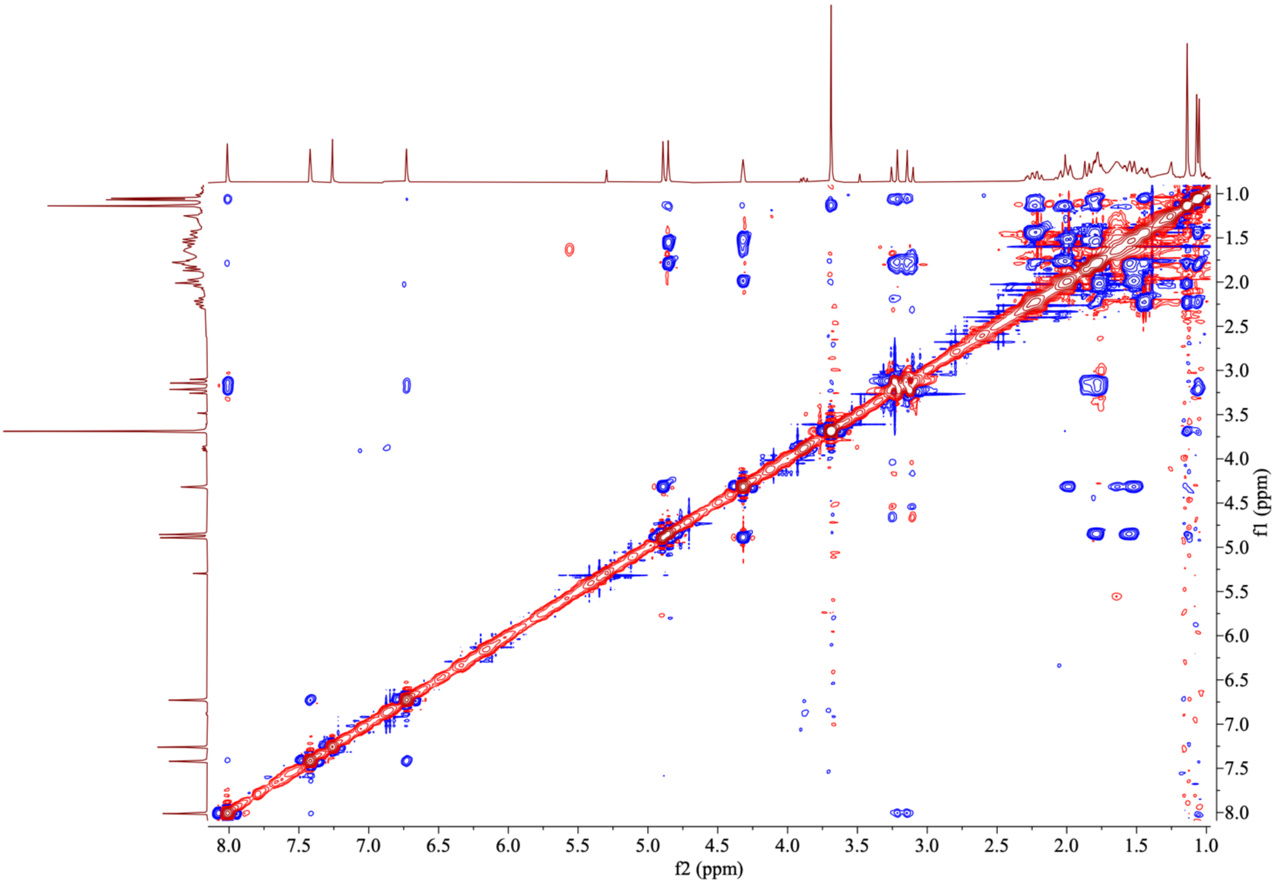

S4. HRMS spectra of new compounds **1–6**.

S4.1. HRESIMS data of compound **1**.

Measured region for 369.1676 m/z

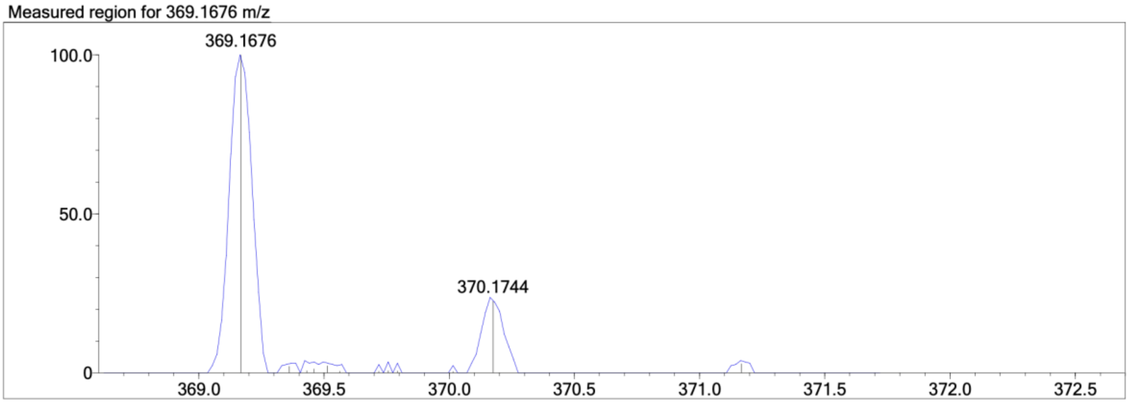

C20 H26 O5 [M+Na]+ : Predicted region for 369.1672 m/z

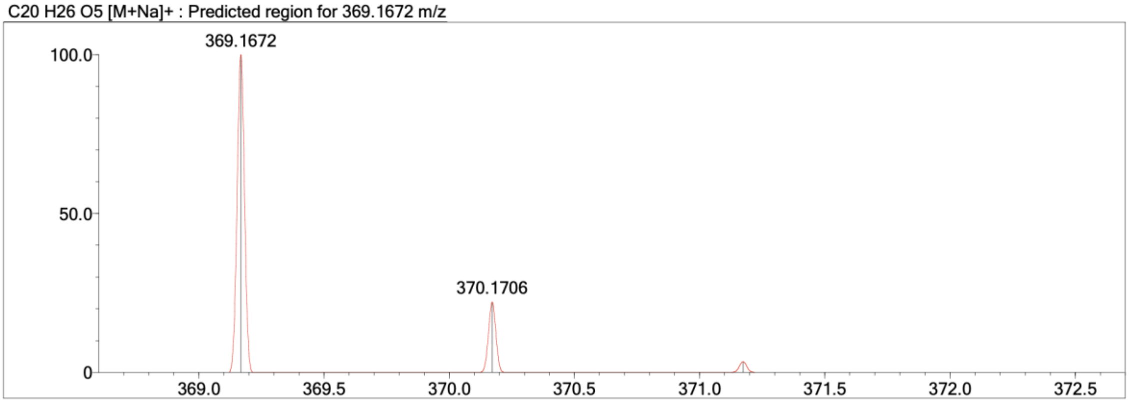

Rank	Score	Formula (M)	Ion	Meas. m/z	Pred. m/z	Df. (mDa)	Df. (ppm)	Iso	DBE
1	80.59	C20 H26 O5	[M+Na]+	369.1676	369.1672	0.4	1.08	80.75	8.0

S4.2. HRESIMS data of compound **2**.

Measured region for 383.1826 m/z

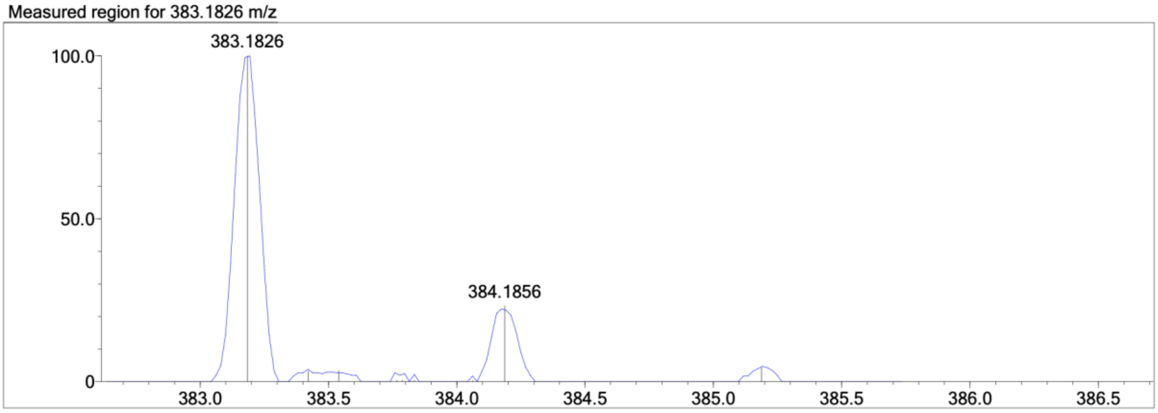

C21 H28 O5 [M+Na]+ : Predicted region for 383.1829 m/z

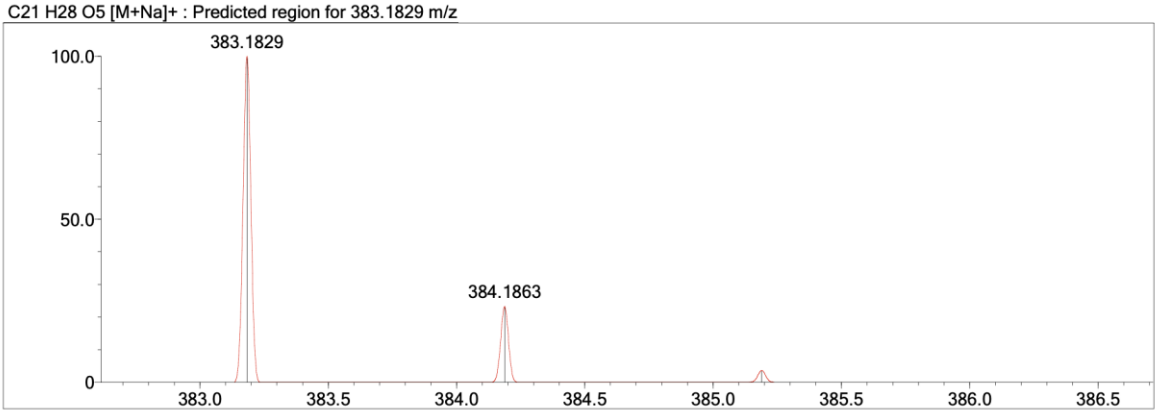

Rank	Score	Formula (M)	Ion	Meas. m/z	Pred. m/z	Df. (mDa)	Df. (ppm)	Iso	DBE
1	80.95	C21 H28 O5	[M+Na]+	383.1826	383.1829	-0.3	-0.78	80.95	8.0

S4.3. HRESIMS data of compound **3**.

Measured region for 383.1829 m/z

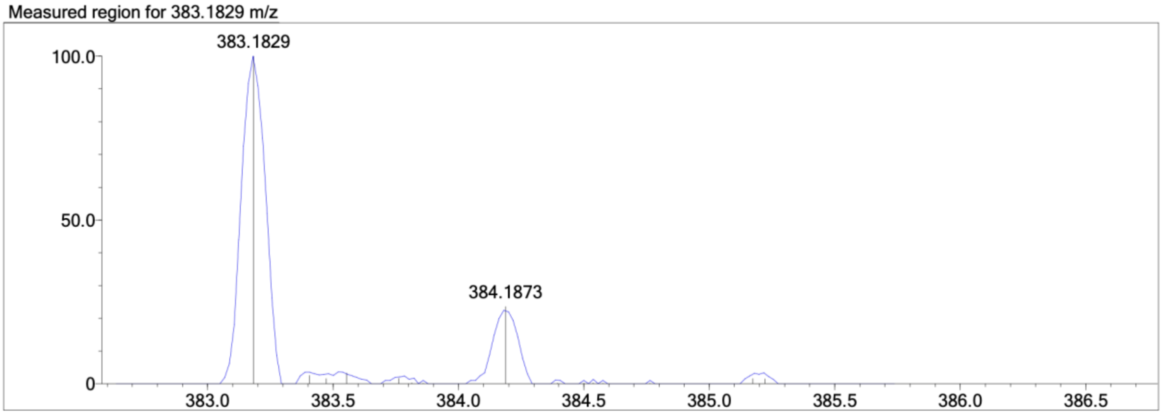

C21 H28 O5 [M+Na]+ : Predicted region for 383.1829 m/z

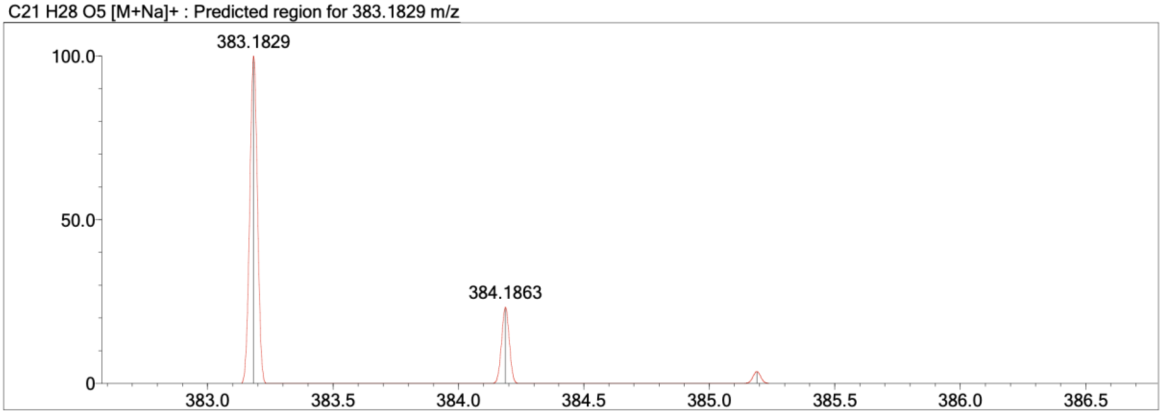

Rank	Score	Formula (M)	Ion	Meas. m/z	Pred. m/z	Df. (mDa)	Df. (ppm)	Iso	DBE
1	82.19	C21 H28 O5	[M+Na]+	383.1829	383.1829	0.0	0.00	82.19	8.0

S4.4. HRESIMS data of compound **4**.

Measured region for 383.1830 m/z

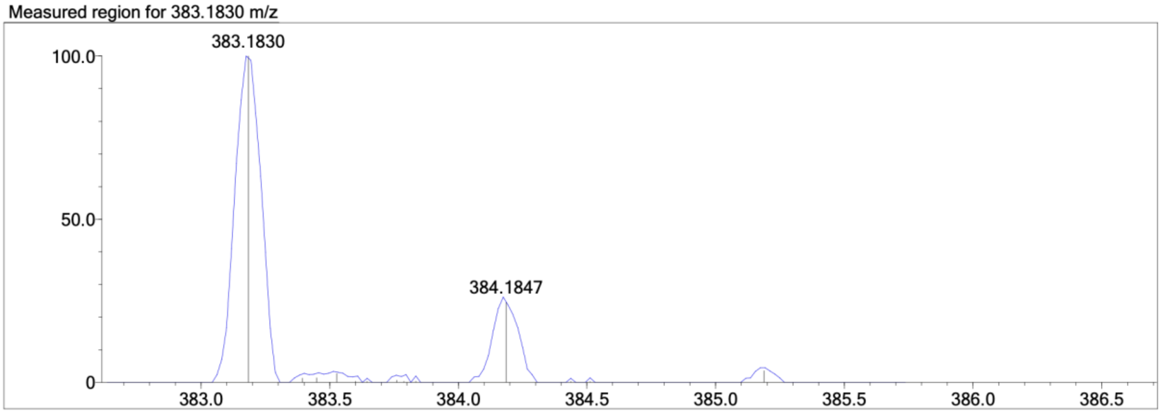

C21 H28 O5 [M+Na]+ : Predicted region for 383.1829 m/z

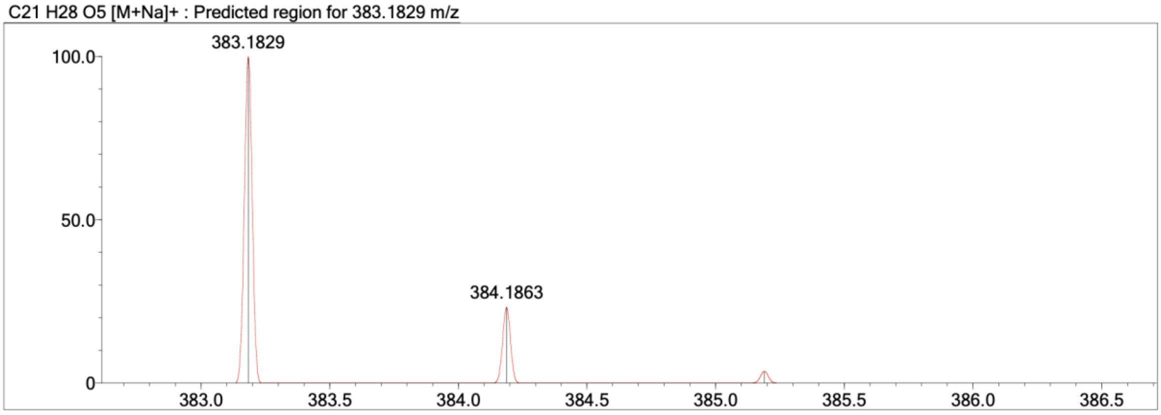

Rank	Score	Formula (M)	Ion	Meas. m/z	Pred. m/z	Df. (mDa)	Df. (ppm)	Iso	DBE
1	74.79	C21 H28 O5	[M+Na]+	383.1830	383.1829	0.1	0.26	74.79	8.0

S4.5. HRESIMS data of compound **5**.

Measured region for 415.2092 m/z

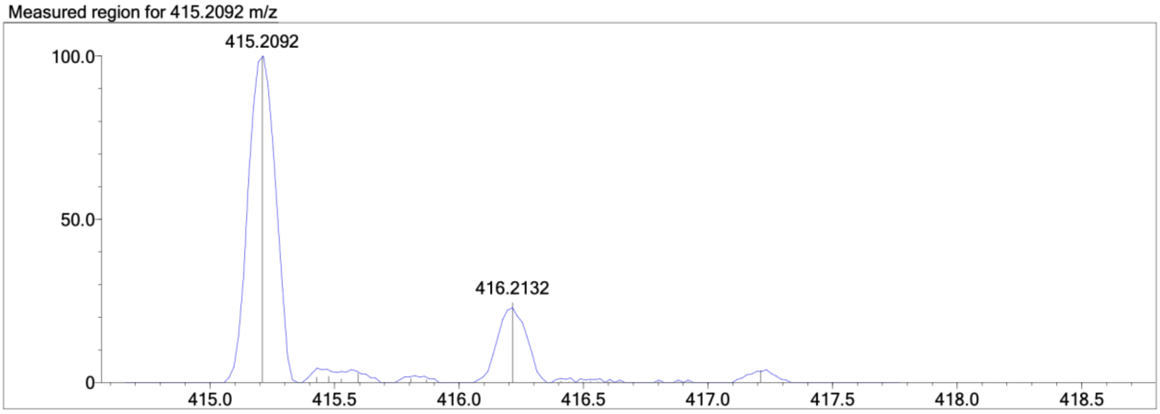

C22 H32 O6 [M+Na]+ : Predicted region for 415.2091 m/z

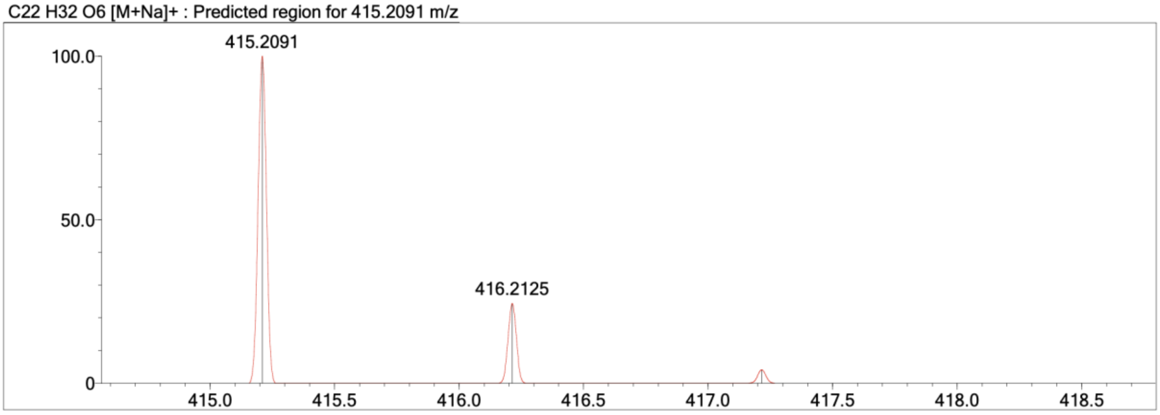

Rank	Score	Formula (M)	Ion	Meas. m/z	Pred. m/z	Df. (mDa)	Df. (ppm)	Iso	DBE
1	81.59	C22 H32 O6	[M+Na]+	415.2092	415.2091	0.1	0.24	81.59	7.0

S4.6. HRESIMS data of compound **6**.

Measured region for 383.1829 m/z

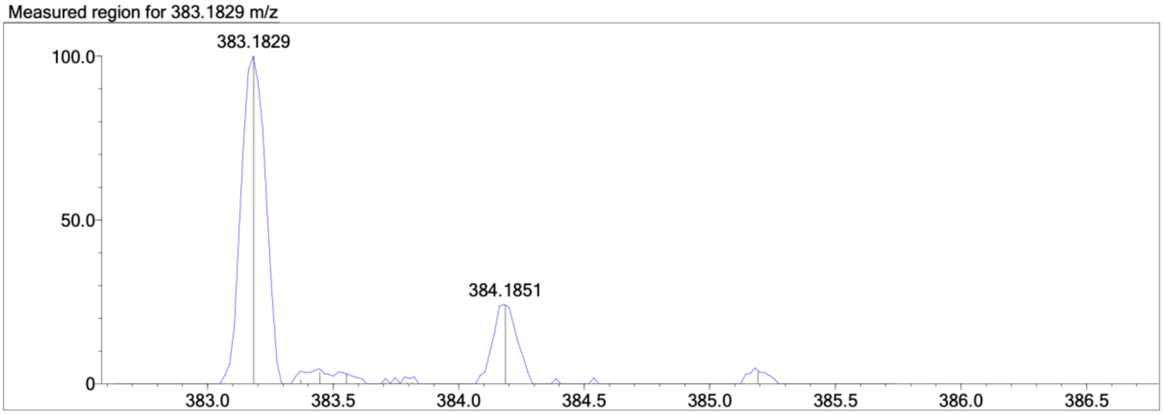

C21 H28 O5 [M+Na]+ : Predicted region for 383.1829 m/z

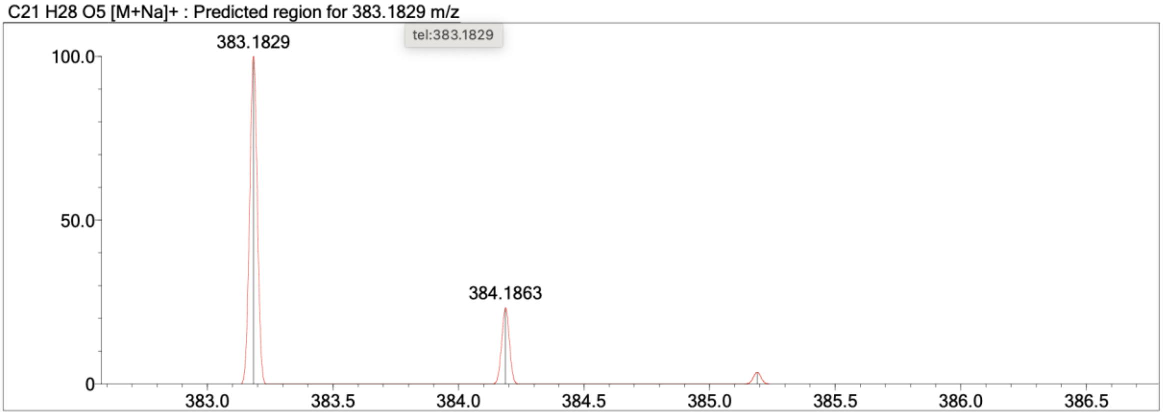

Rank	Score	Formula (M)	Ion	Meas. m/z	Pred. m/z	Df. (mDa)	Df. (ppm)	Iso	DBE
1	81.60	C21 H28 O5	[M+Na]+	383.1829	383.1829	0.0	0.00	81.60	8.0
